# Supplementary material for: N-(Hydroxyalkyl) Derivatives of tris(1H-indol-3-yl)methylium Salts as Promising Antibacterial Agents: Synthesis and Biological Evaluation
Source: Pharmaceuticals (Basel). 2020 Dec 16;13(12):469. doi: 10.3390/ph13120469 (PMC7765952; doi:10.3390/ph13120469)

2-(1H-Indol-1-yl)-ethanol (3a)

^{13}C NMR (101 MHz, dmso) δ 136.33, 129.59, 128.53, 121.26, 120.73, 119.24, 110.32, 100.67, 100.65, 60.76, 48.66, 48.64.

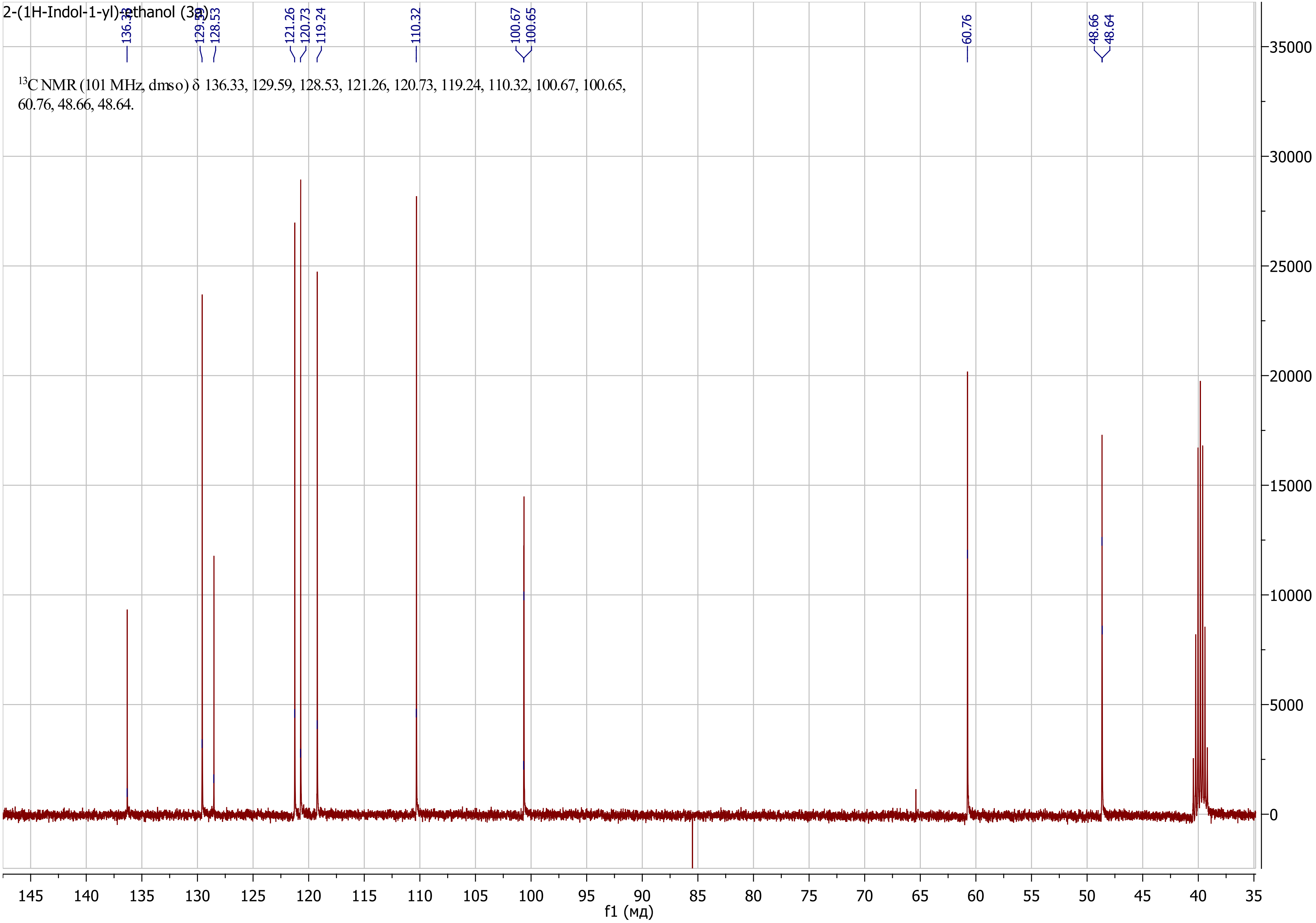

2-(1H-Indol-1-yl)ethanol (3a)

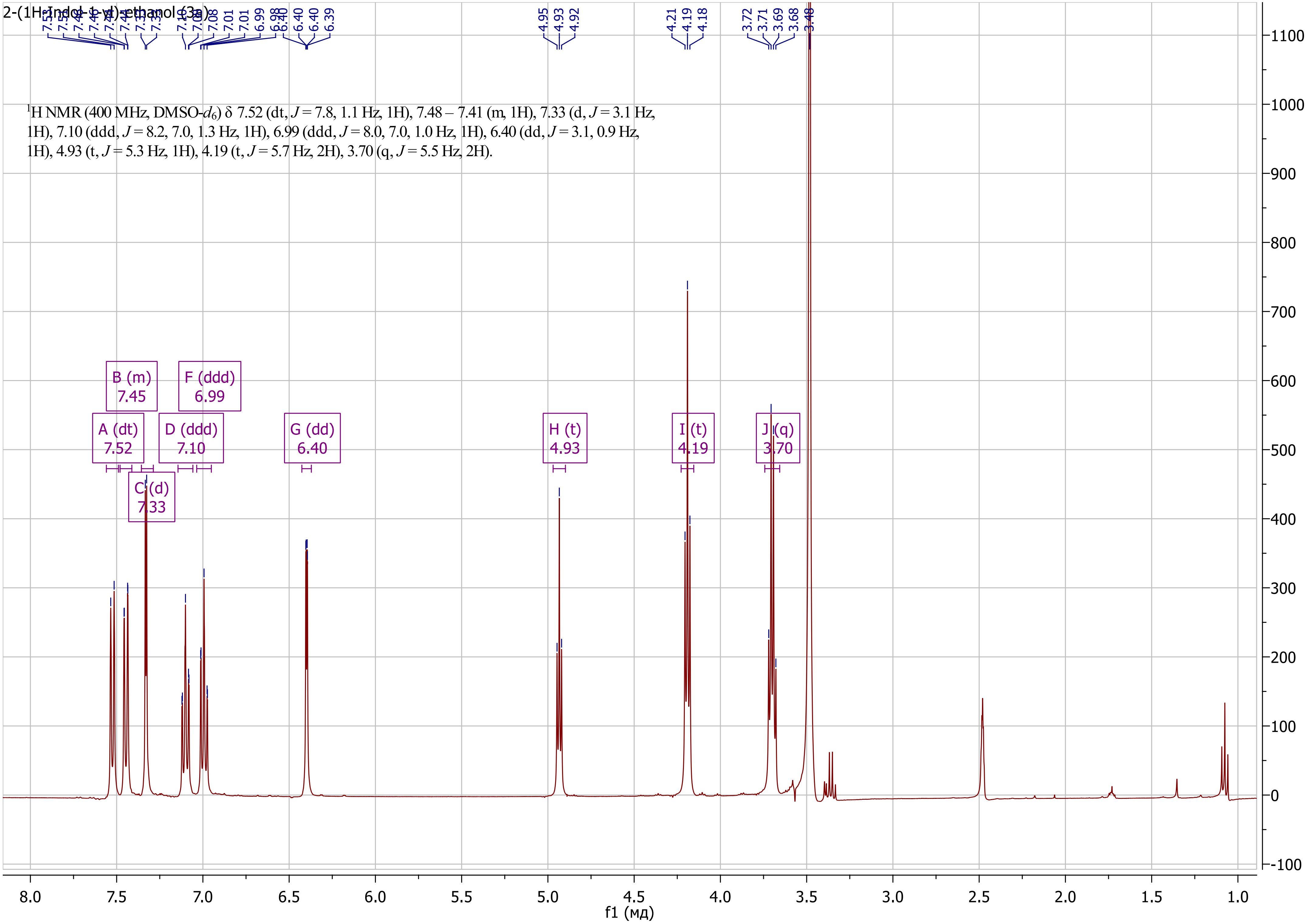

^{1}H NMR (400 MHz, DMSO-d_6) δ 7.52 (dt, J = 7.8, 1.1 Hz, 1H), 7.48 – 7.41 (m, 1H), 7.33 (d, J = 3.1 Hz, 1H), 7.10 (ddd, J = 8.2, 7.0, 1.3 Hz, 1H), 6.99 (ddd, J = 8.0, 7.0, 1.0 Hz, 1H), 6.40 (dd, J = 3.1, 0.9 Hz, 1H), 4.93 (t, J = 5.3 Hz, 1H), 4.19 (t, J = 5.7 Hz, 2H), 3.70 (q, J = 5.5 Hz, 2H).

3-(1H-Indol-1-yl)propan-1-ol (3b)

^{13}C NMR (101 MHz, dmso) δ 136.08, 129.12, 128.53, 121.38, 120.85, 119.27, 110.17, 100.82, 100.80, 58.27, 42.84, 33.43.

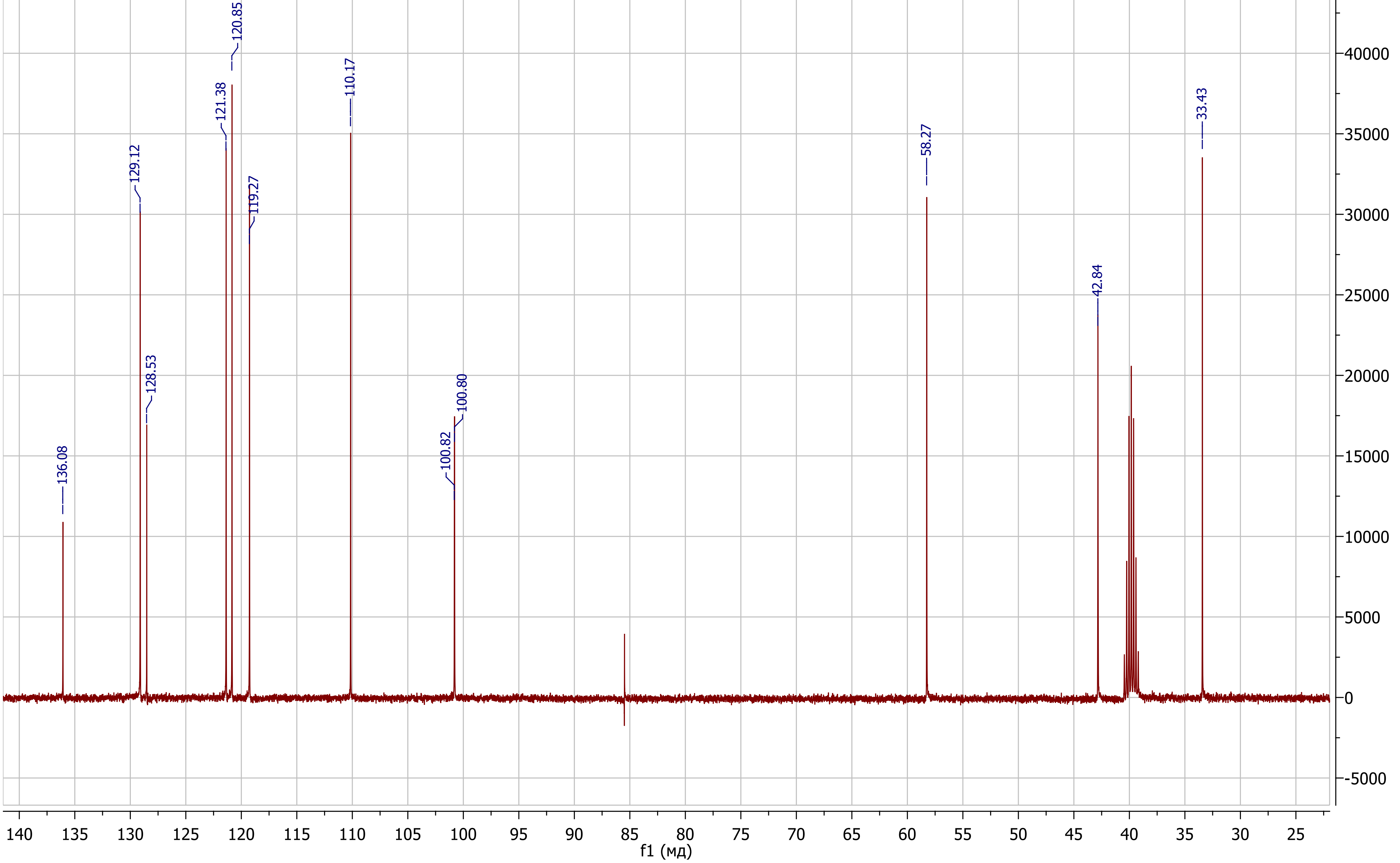

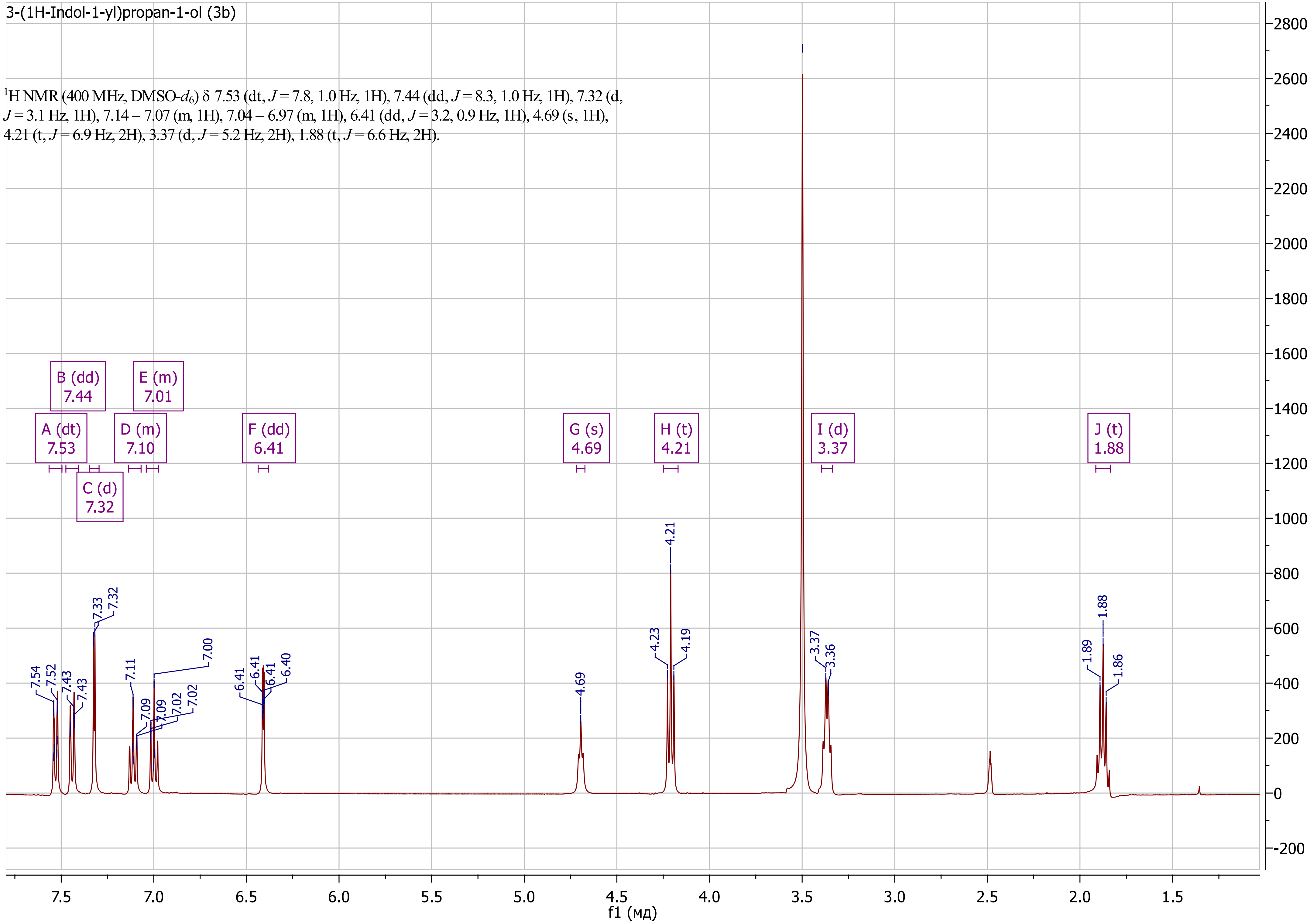
3-(1H-Indol-1-yl)propan-1-ol (3b)
^{1}H NMR (400 MHz, DMSO-d_6) δ 7.53 (dt, J = 7.8, 1.0 Hz, 1H), 7.44 (dd, J = 8.3, 1.0 Hz, 1H), 7.32 (d, J = 3.1 Hz, 1H), 7.14 – 7.07 (m, 1H), 7.04 – 6.97 (m, 1H), 6.41 (dd, J = 3.2, 0.9 Hz, 1H), 4.69 (s, 1H), 4.21 (t, J = 6.9 Hz, 2H), 3.37 (d, J = 5.2 Hz, 2H), 1.88 (t, J = 6.6 Hz, 2H).
A (dt) 7.53
B (dd) 7.44
C (d) 7.32
D (m) 7.10
E (m) 7.01
F (dd) 6.41
G (s) 4.69
H (t) 4.21
I (d) 3.37
J (t) 1.88
f1 (мд)

4-(1H-Indol-1-yl)butan-1-ol (3c)

^{1}H NMR (400 MHz, DMSO-d_6) δ 7.53 (dt, J = 7.8, 1.0 Hz, 1H), 7.44 (dd, J = 8.3, 1.1 Hz, 1H), 7.33 (d, J = 3.1 Hz, 1H), 7.11 (ddd, J = 8.2, 6.9, 1.2 Hz, 1H), 7.00 (ddd, J = 8.0, 7.0, 1.0 Hz, 1H), 6.41 (dd, J = 3.1, 0.9 Hz, 1H), 4.47 (t, J = 5.1 Hz, 1H), 4.15 (t, J = 7.1 Hz, 2H), 3.39 (td, J = 6.4, 4.9 Hz, 2H), 1.77 (dq, J = 9.6, 7.2 Hz, 2H), 1.43 – 1.32 (m, 2H).

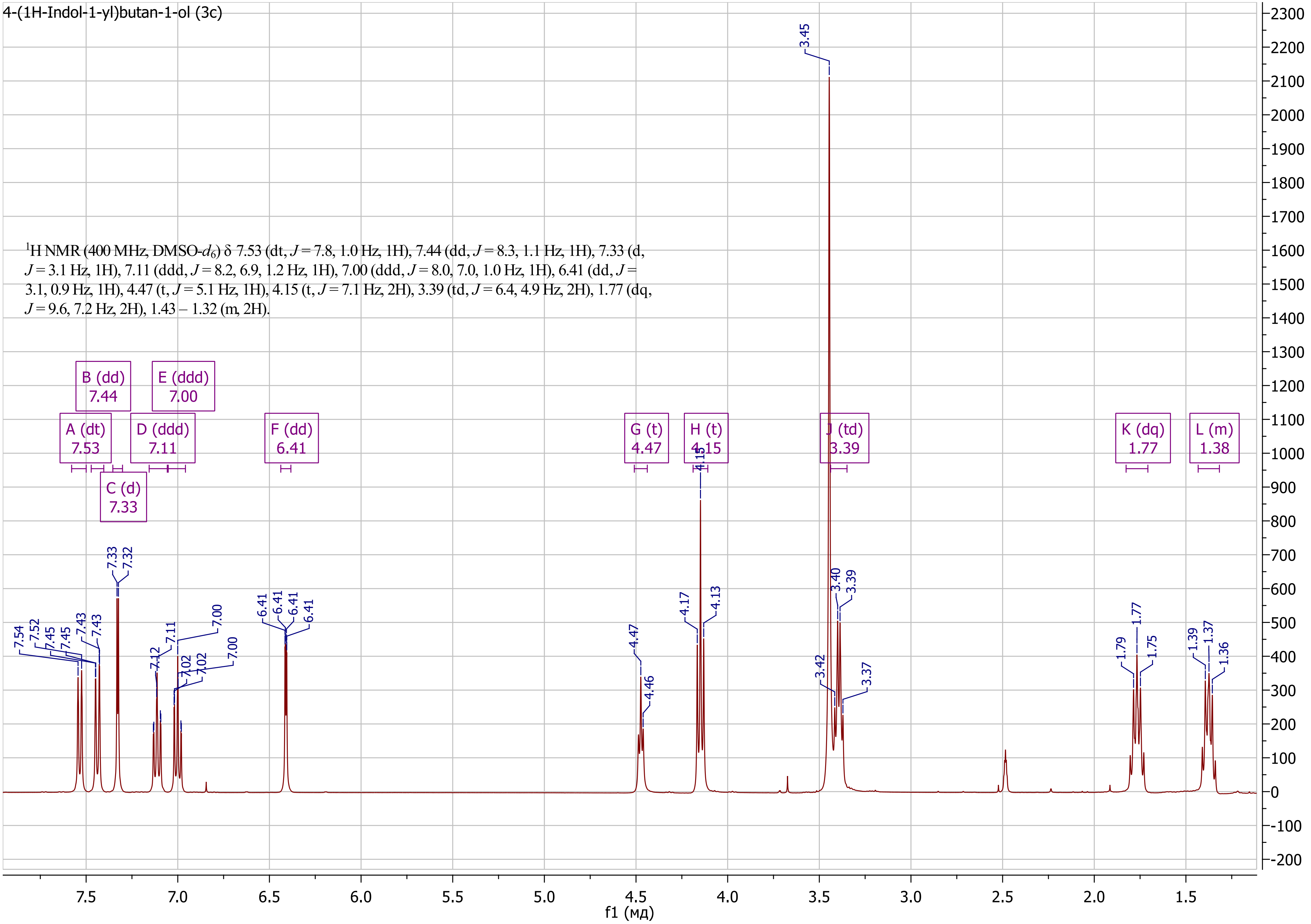

4-(1H-Indol-1-yl)butan-1-ol (3c)

^{13}C NMR (101 MHz, dmso) δ 136.11, 129.00, 128.57, 121.34, 120.85, 119.23, 110.20, 100.79, 100.76, 60.80, 45.84, 40.53, 40.33, 40.12, 39.91, 39.70, 39.49, 39.28, 30.21, 27.09.

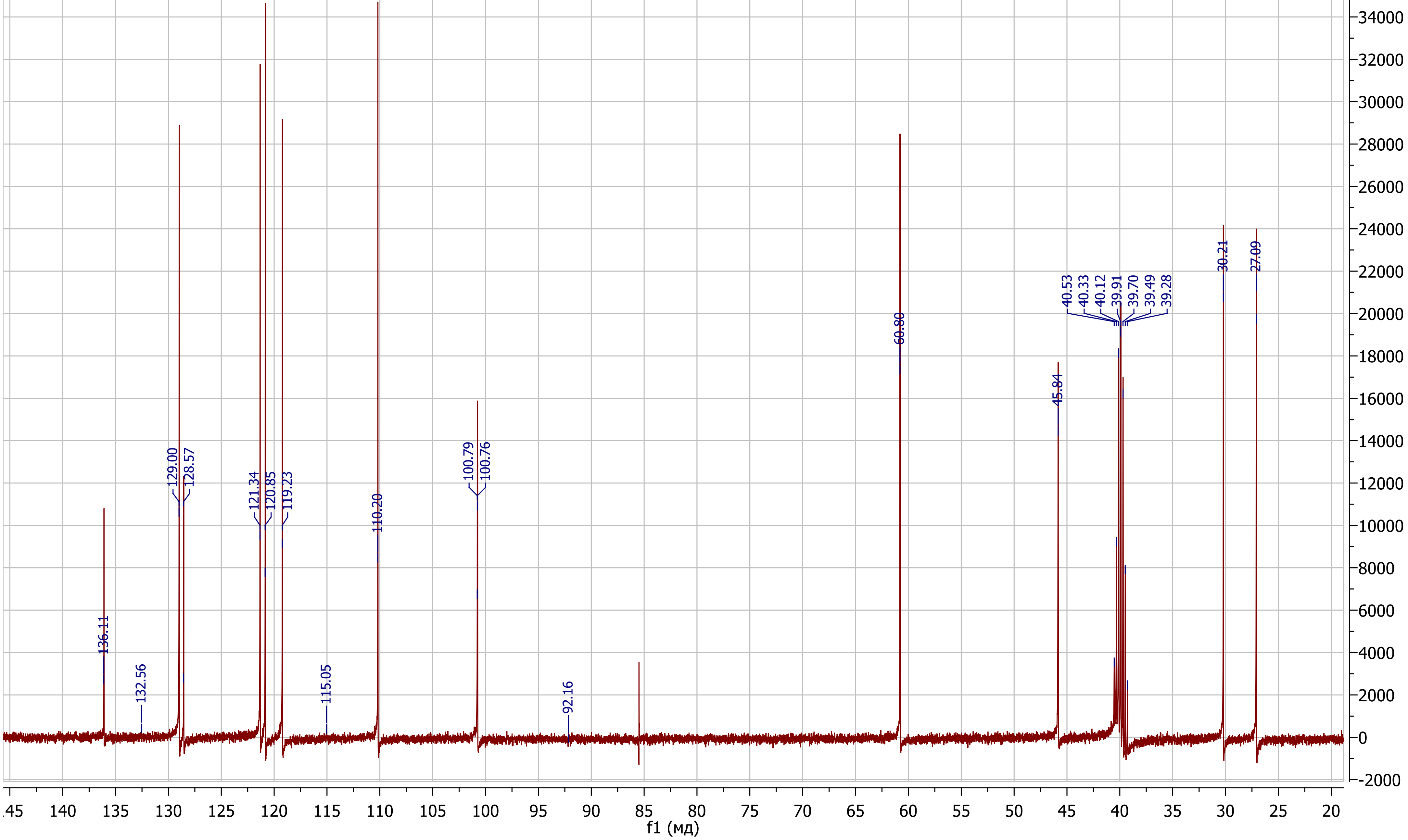

5-(1H-Indol-1-yl)pentan-1-ol (3d)

^{13}C NMR (101 MHz, dmso) δ 136.10, 128.97, 128.57, 121.35, 120.86, 119.23, 110.14, 100.80, 100.77, 85.50, 61.06, 45.95, 45.93, 32.54, 30.23, 23.37.

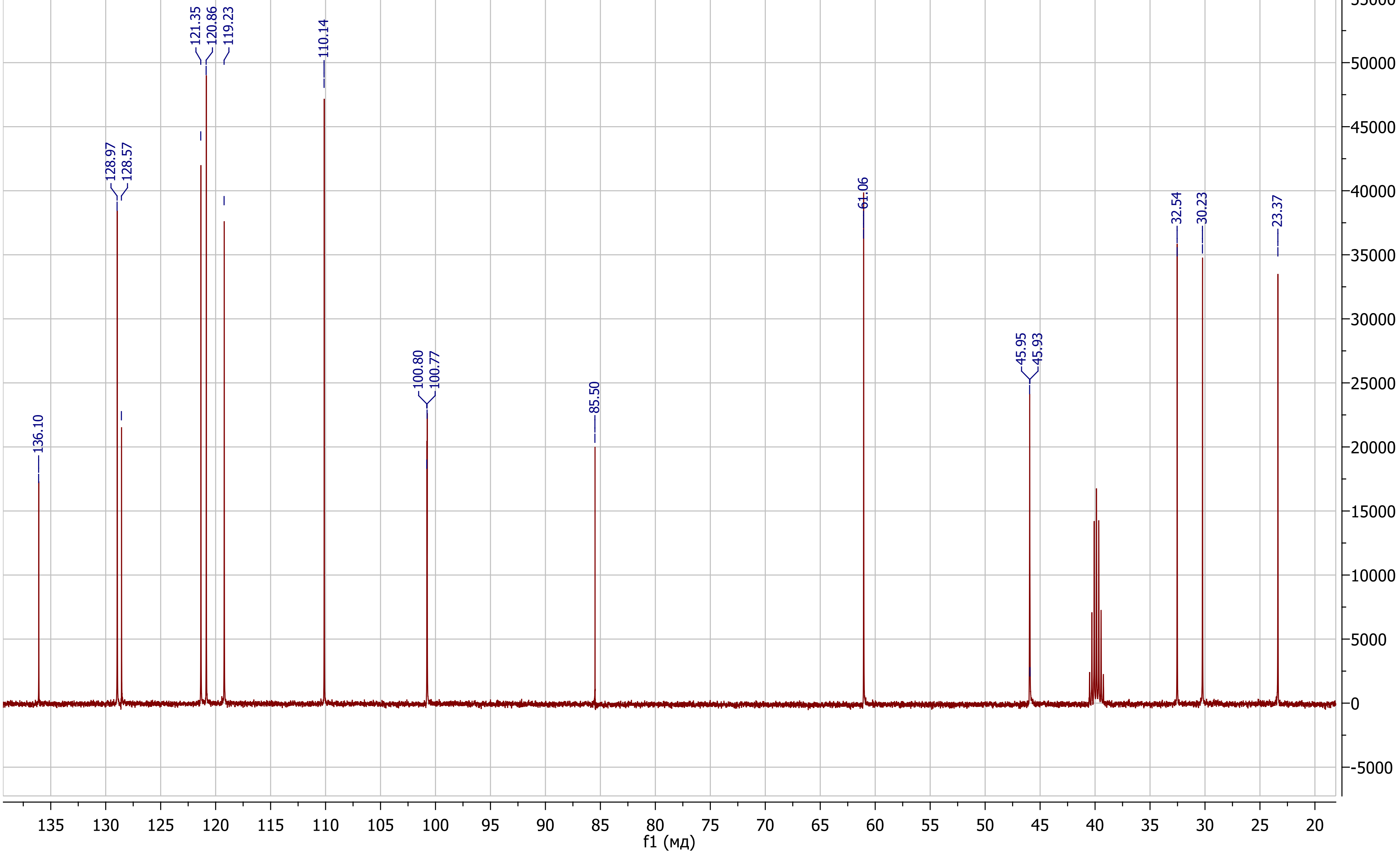

5-(1H-Indol-1-yl)pentan-1-ol (3d)

^{1}H NMR (400 MHz, DMSO-d_6) δ 7.54 (dt, J = 7.9, 1.0 Hz, 1H), 7.42 (dd, J = 8.2, 1.1 Hz, 1H), 7.31 (d, J = 3.2 Hz, 1H), 7.12 (ddd, J = 8.2, 7.0, 1.3 Hz, 1H), 7.01 (ddd, J = 8.0, 6.9, 1.0 Hz, 1H), 6.41 (dd, J = 3.1, 0.9 Hz, 1H), 4.43 (s, 1H), 4.11 (t, J = 7.0 Hz, 2H), 3.37 (t, J = 6.5 Hz, 2H), 1.73 (p, J = 7.2 Hz, 2H), 1.49 – 1.37 (m, 2H), 1.33 – 1.19 (m, 2H).

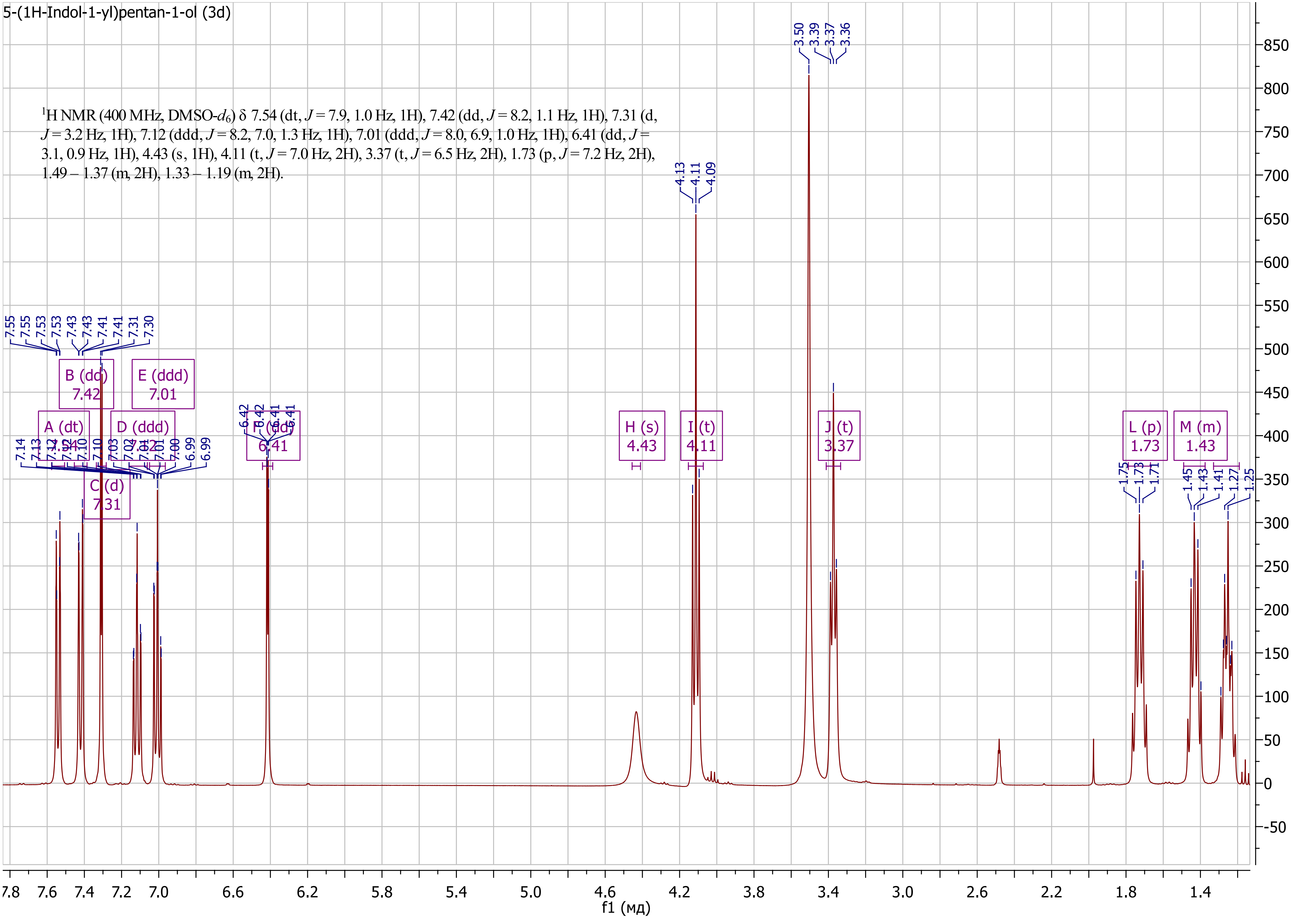

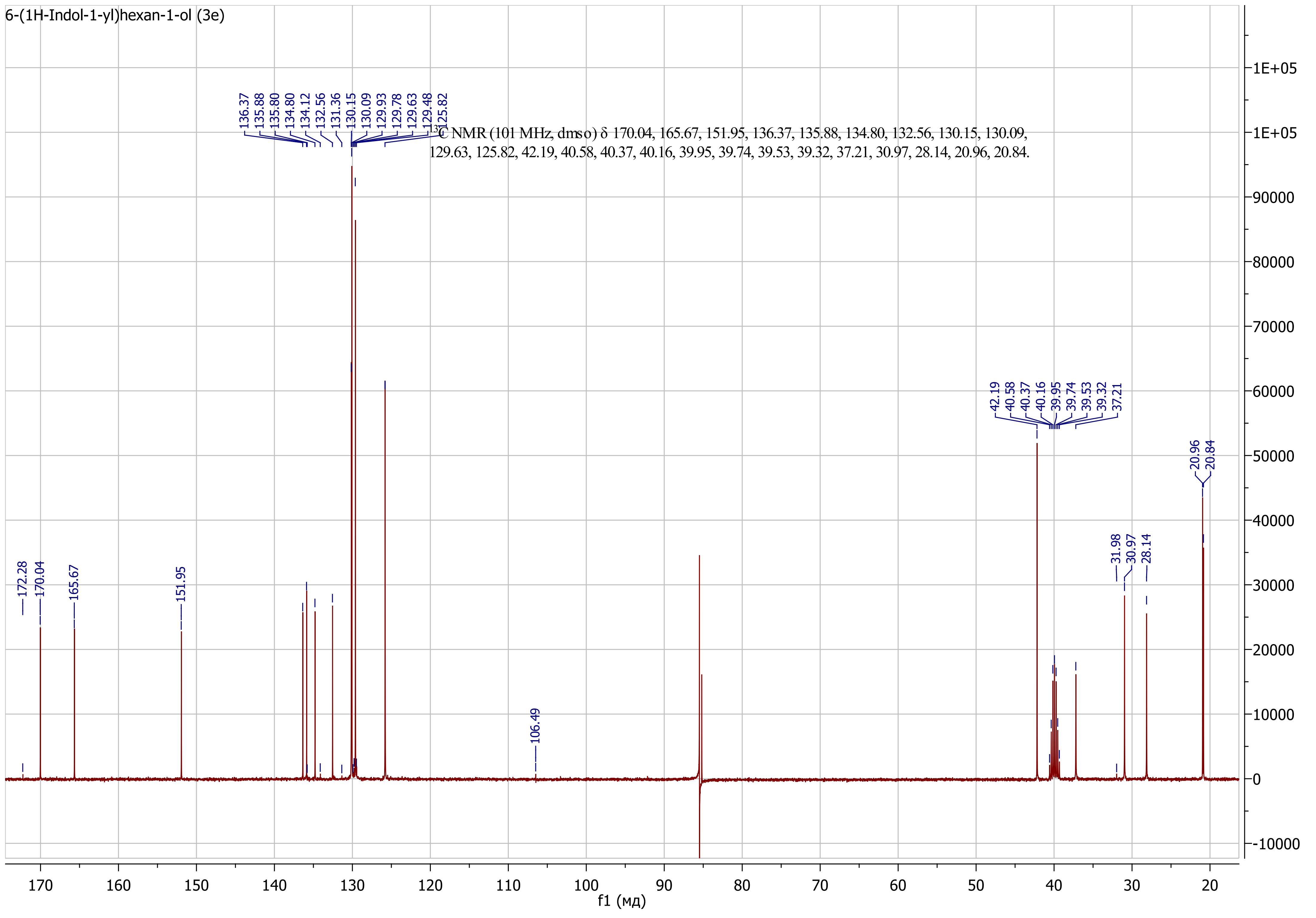
6-(1H-Indol-1-yl)hexan-1-ol (3e)
13C NMR (101 MHz, dmso) δ 170.04, 165.67, 151.95, 136.37, 135.88, 134.80, 132.56, 130.15, 130.09, 129.63, 125.82, 42.19, 40.58, 40.37, 40.16, 39.95, 39.74, 39.53, 39.32, 37.21, 30.97, 28.14, 20.96, 20.84.
136.37
135.88
135.80
134.80
134.12
132.56
131.36
130.15
130.09
129.93
129.78
129.63
129.48
125.82
172.28
170.04
165.67
151.95
106.49
42.19
40.58
40.37
40.16
39.95
39.74
39.53
39.32
37.21
31.98
30.97
28.14
20.96
20.84
f1 (мд)

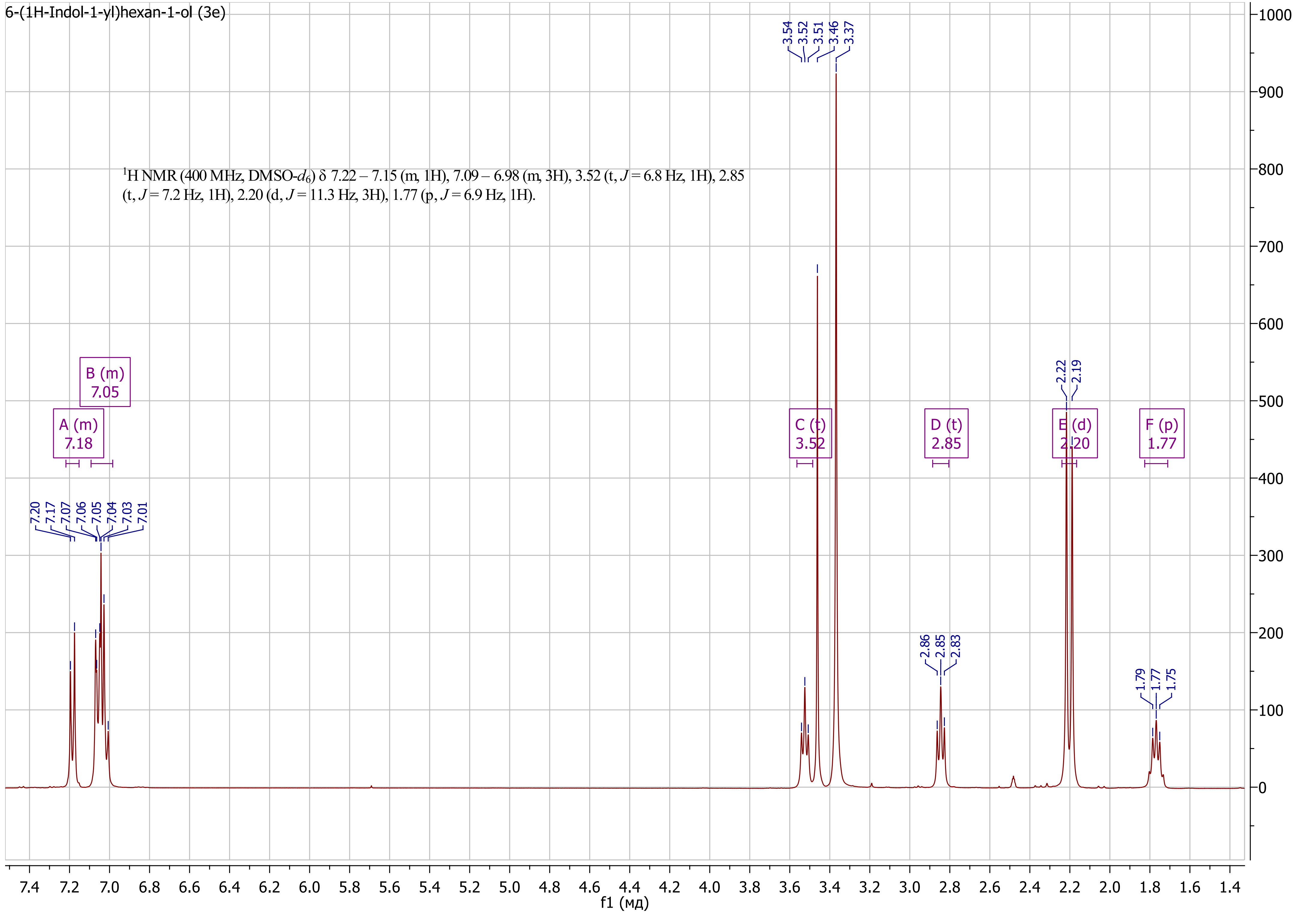
6-(1H-Indol-1-yl)hexan-1-ol (3e)
1H NMR (400 MHz, DMSO-d6) δ 7.22 – 7.15 (m, 1H), 7.09 – 6.98 (m, 3H), 3.52 (t, J = 6.8 Hz, 1H), 2.85 (t, J = 7.2 Hz, 1H), 2.20 (d, J = 11.3 Hz, 3H), 1.77 (p, J = 6.9 Hz, 1H).
A (m) 7.18
B (m) 7.05
C (t) 3.52
D (t) 2.85
E (d) 2.20
F (p) 1.77
f1 (мд)

2-(1H-Indol-1-yl)ethyl acetate (4a)

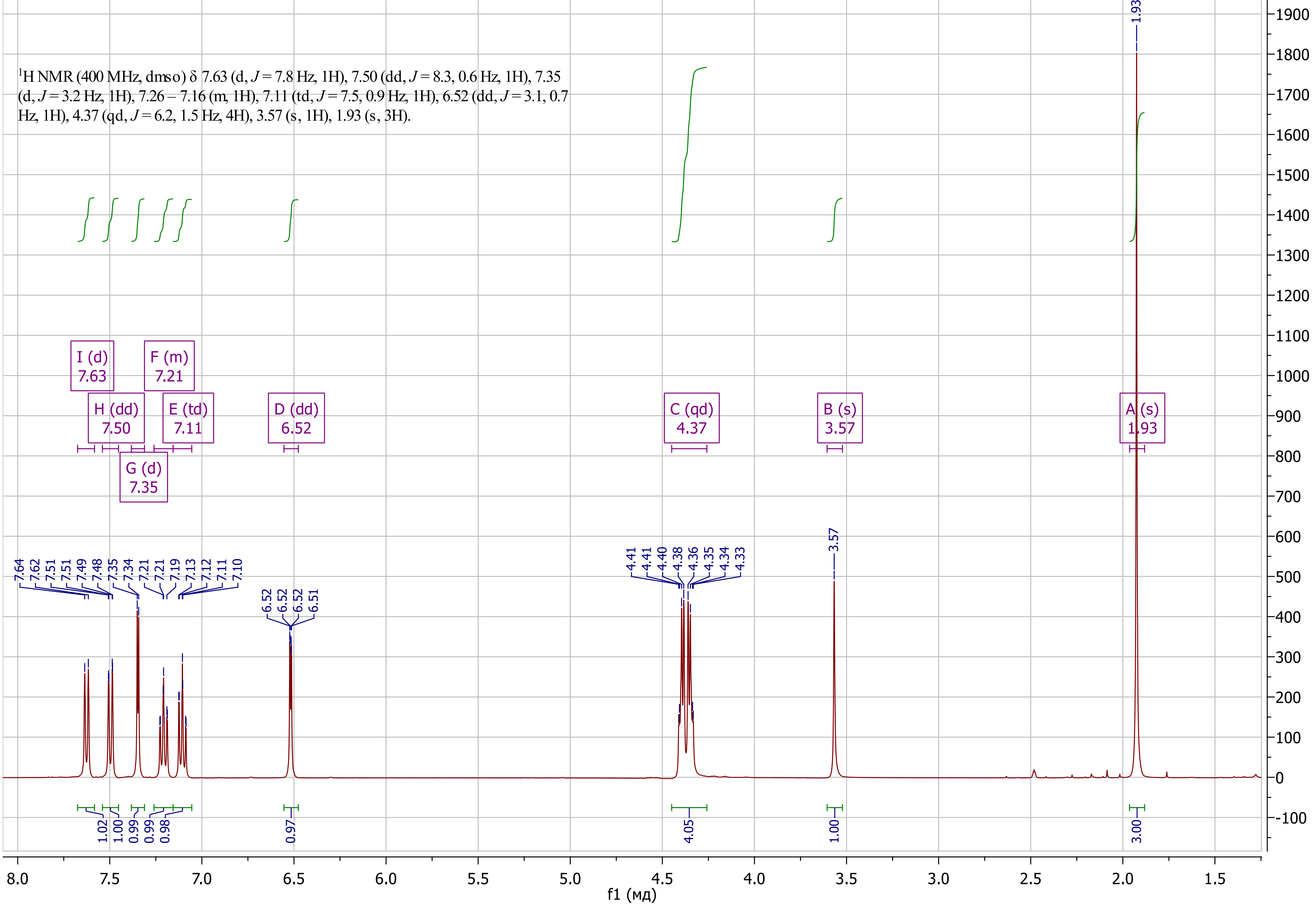

2-(1H-Indol-1-yl)ethyl acetate (4a)

^{13}C NMR (101 MHz, dmso) δ = 170.53, 136.43, 129.16, 128.75, 121.63, 120.96, 119.58, 110.09, 101.50, 63.40, 44.93, 20.86.

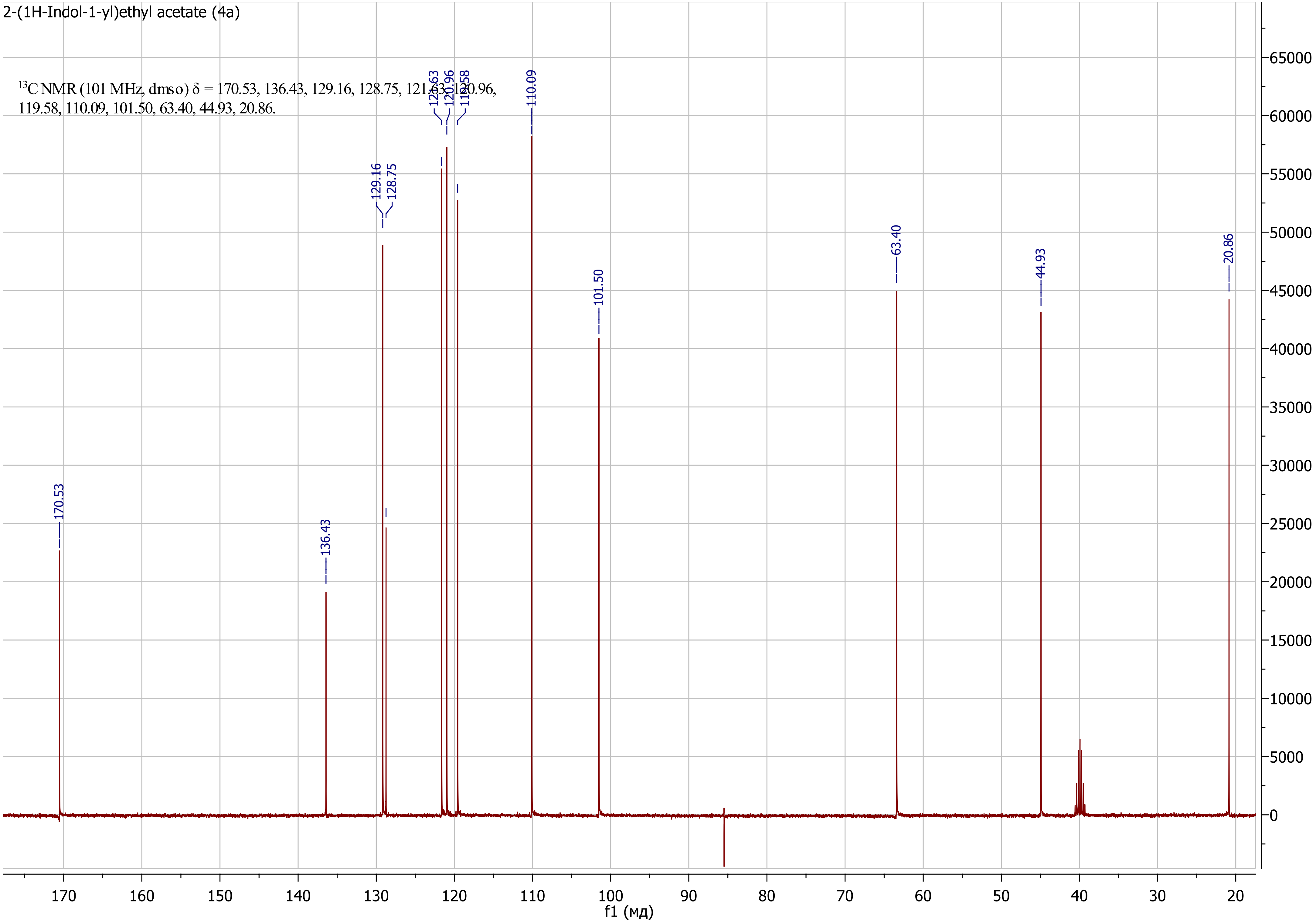

3-(1H-Indol-1-yl)propyl acetate (4b)

^{1}H NMR (400 MHz, dmso) δ 7.62 (d, J = 7.8 Hz, 1H), 7.46 (d, J = 8.2 Hz, 1H), 7.31 (d, J = 3.1 Hz, 1H), 7.24 – 7.15 (m, 1H), 7.15 – 7.06 (m, 1H), 6.55 – 6.47 (m, 1H), 4.22 (t, J = 6.8 Hz, 2H), 3.96 (t, J = 6.4 Hz, 2H), 2.14 – 1.96 (m, 5H).

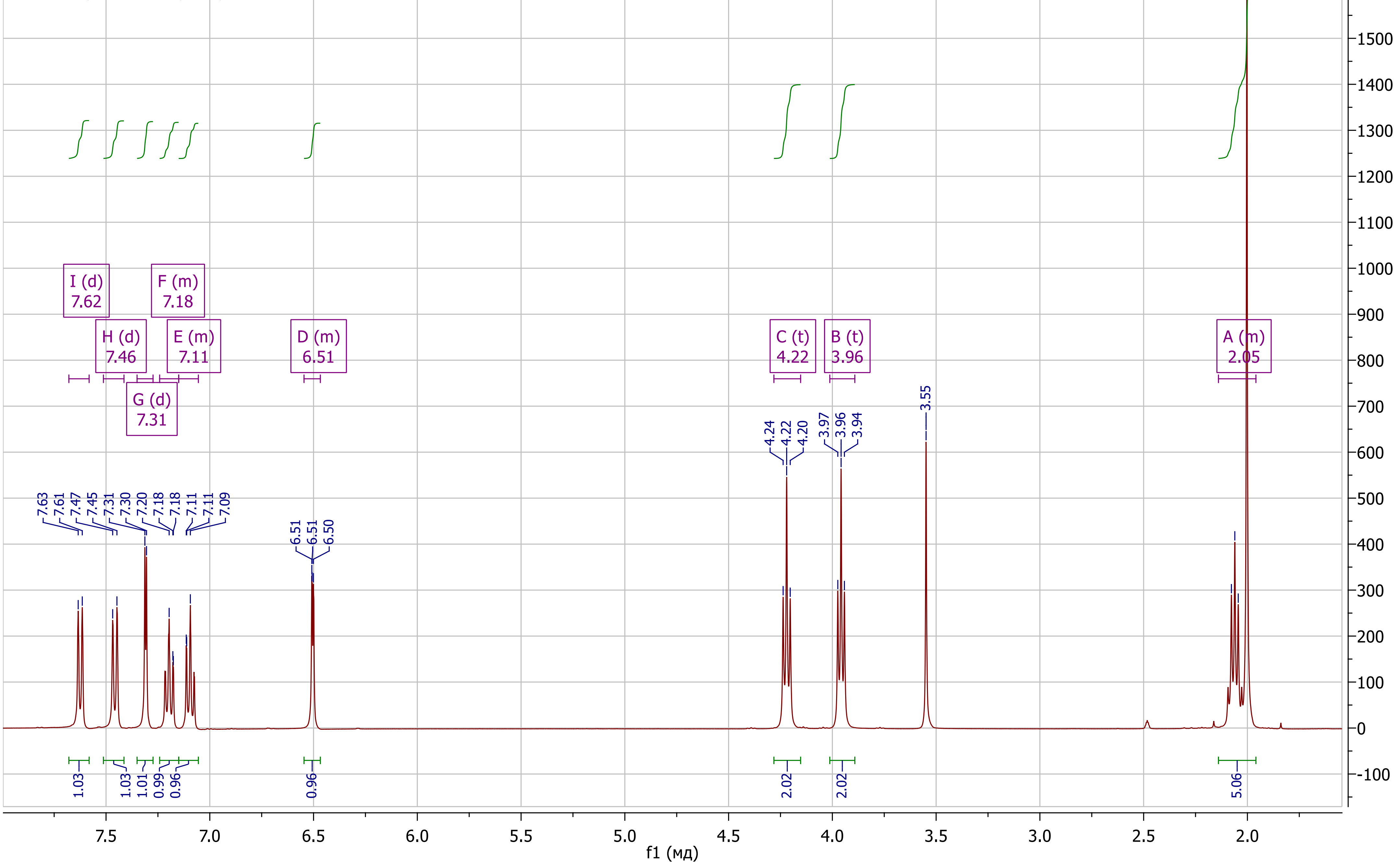

3-(1H-Indol-1-yl)propyl acetate (4b)

^{13}C NMR (101 MHz, dmso) δ = 170.75, 136.20, 128.81, 128.74, 121.54, 120.97, 119.43, 110.00, 101.23, 61.71, 42.73, 29.29, 20.92.

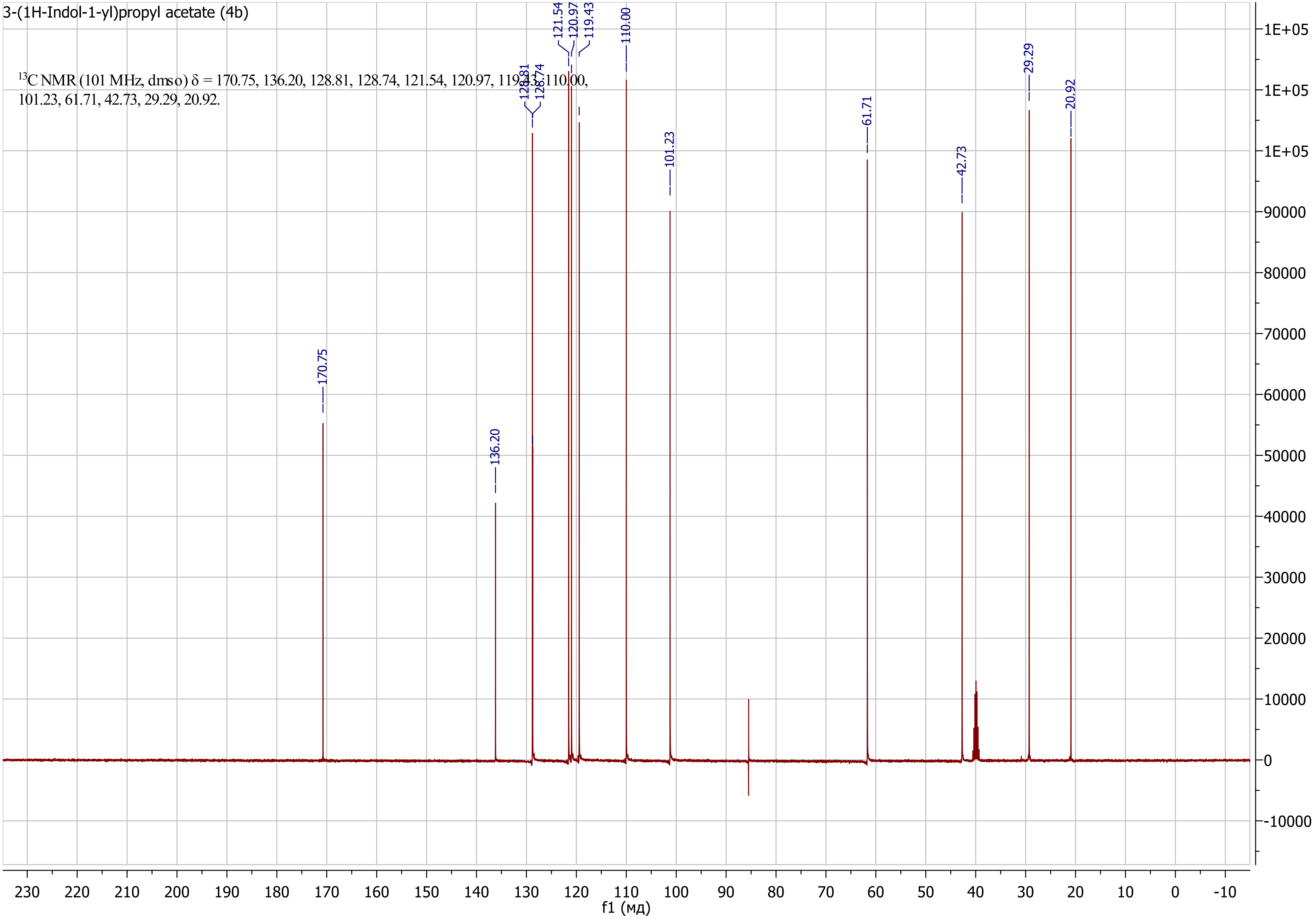

4-(1H-Indol-1-yl)butyl acetate (4c)

^{1}H NMR (400 MHz, dmso) δ 7.53 (d, J = 7.8 Hz, 1H), 7.47 – 7.39 (m, 1H), 7.32 (d, J = 3.1 Hz, 1H), 7.15 – 7.05 (m, 1H), 7.04 – 6.96 (m, 1H), 6.41 (dd, J = 3.1, 0.7 Hz, 1H), 4.15 (t, J = 7.0 Hz, 2H), 3.95 (t, J = 6.6 Hz, 2H), 1.94 (s, 3H), 1.92 – 1.69 (m, 2H), 1.69 – 1.42 (m, 2H).

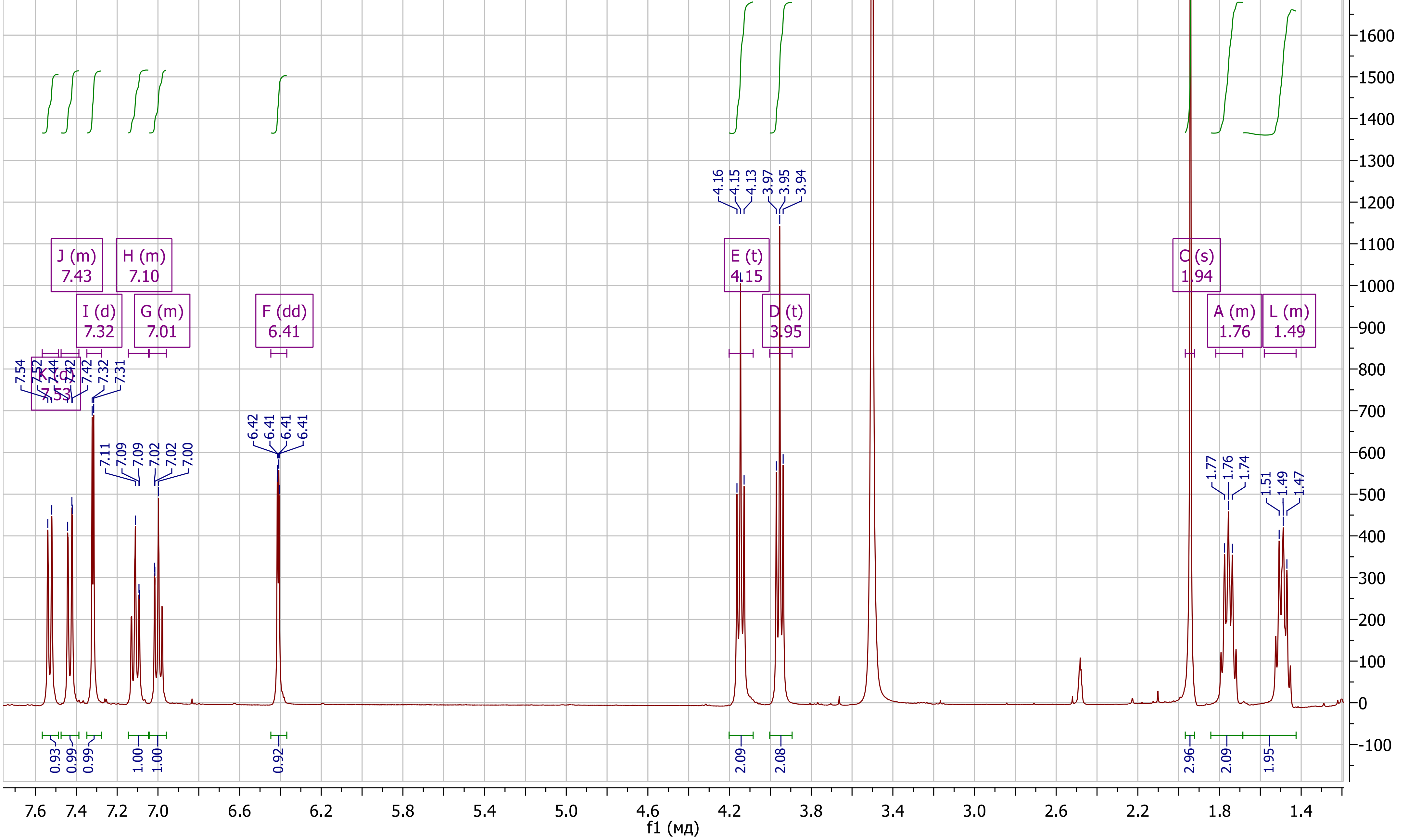

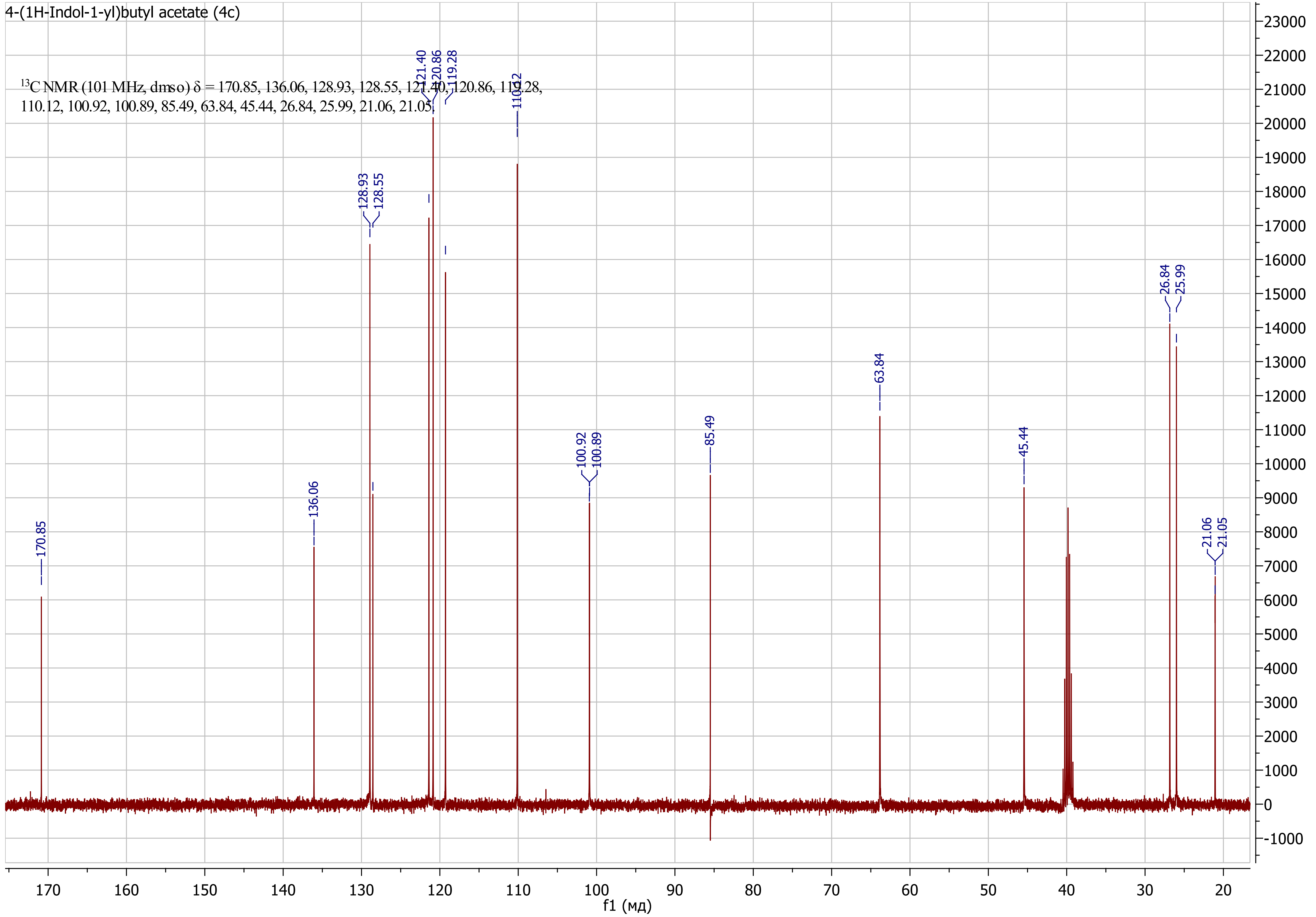
4-(1H-Indol-1-yl)butyl acetate (4c)
13C NMR (101 MHz, dmso) δ = 170.85, 136.06, 128.93, 128.55, 121.40, 120.86, 119.28, 110.12, 100.92, 100.89, 85.49, 63.84, 45.44, 26.84, 25.99, 21.06, 21.05
f1 (мд)

5-(1H-Indol-1-yl)pentyl acetate (4d)

^{1}H NMR (400 MHz, dmso) δ 7.52 (d, J = 7.8 Hz, 1H), 7.47 – 7.38 (m, 1H), 7.32 (d, J = 3.1 Hz, 1H), 7.15 – 7.05 (m, 1H), 7.04 – 6.94 (m, 1H), 6.40 (dd, J = 3.0, 0.6 Hz, 1H), 4.12 (t, J = 7.0 Hz, 2H), 3.92 (t, J = 6.6 Hz, 2H), 1.94 (s, 3H), 1.82 – 1.64 (m, 2H), 1.62 – 1.47 (m, 2H), 1.23 (dd, J = 9.2, 6.2 Hz, 2H).

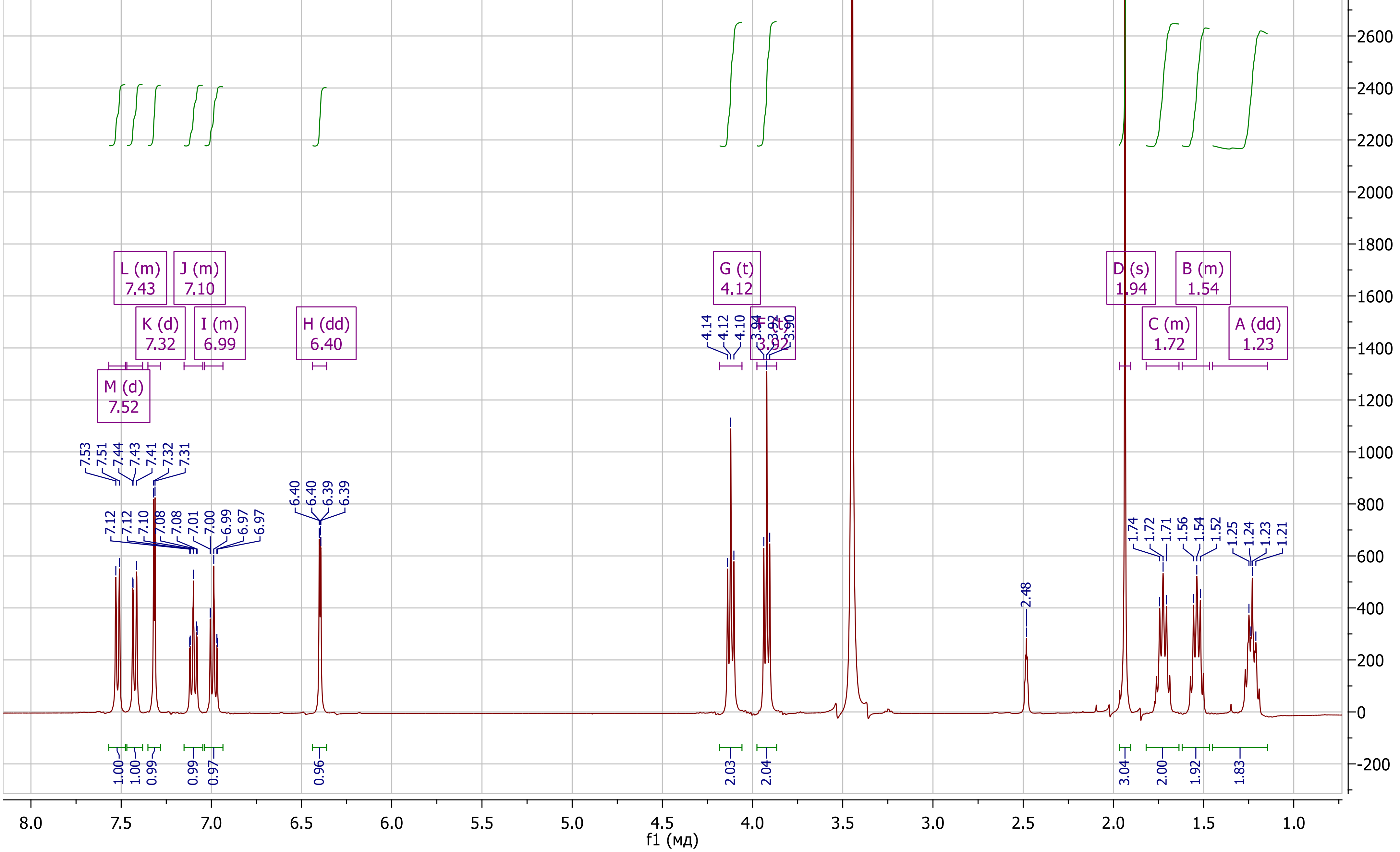

5-(1H-Indol-1-yl)pentyl acetate (4d)

^{13}C NMR (101 MHz, dmso) δ = 170.84, 136.03, 128.97, 128.51, 121.33, 120.83, 119.22, 110.14, 100.77, 64.07, 45.69, 29.86, 28.10, 23.15, 21.10.

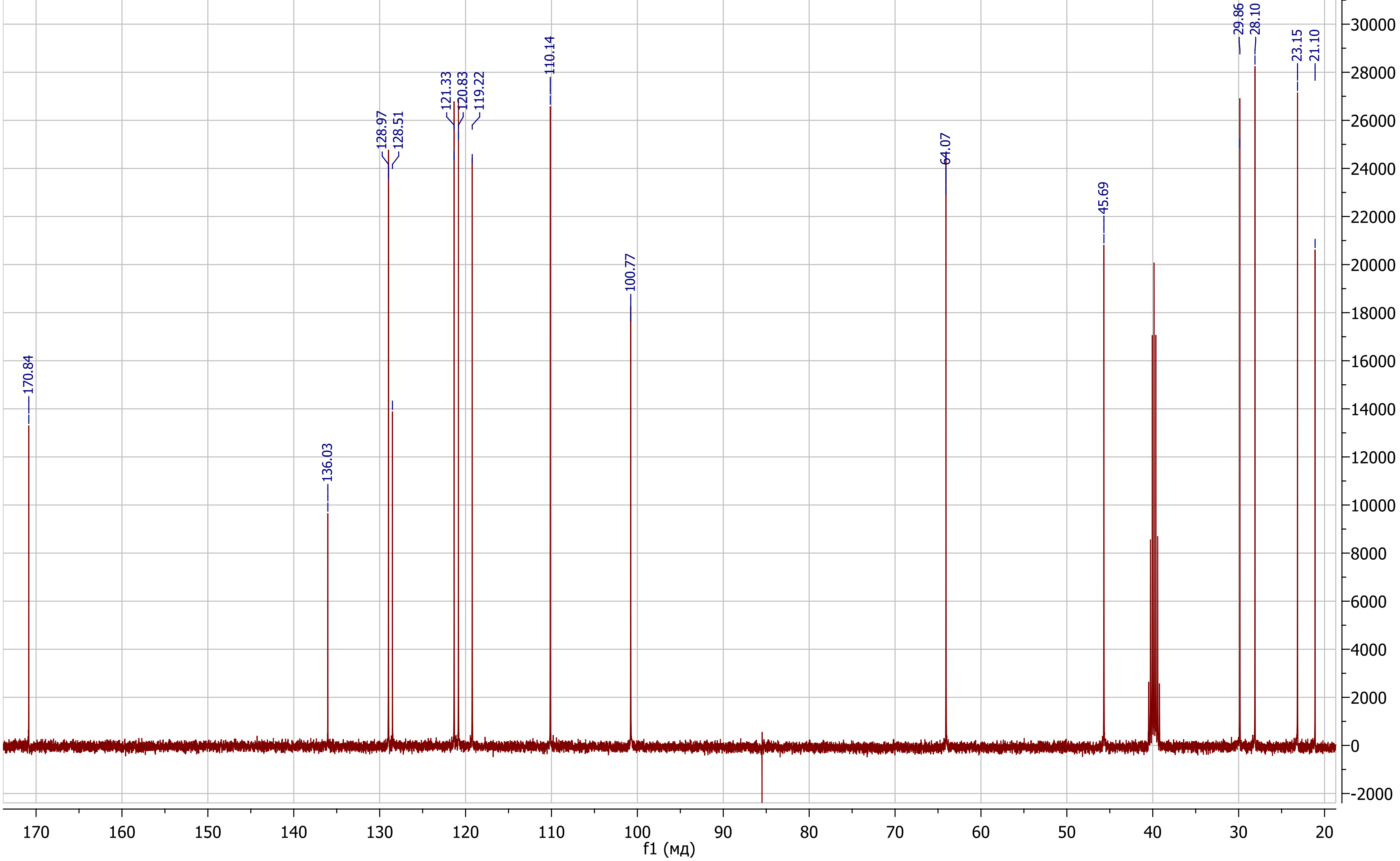

6-(1H-Indol-1-yl)hexyl acetate (4e)

^{1}H NMR (400 MHz, dmso) δ 7.51 (d, J = 7.8 Hz, 2H), 7.42 (d, J = 8.2 Hz, 2H), 7.31 (d, J = 3.0 Hz, 2H), 7.09 (d, J = 7.3 Hz, 2H), 6.99 (d, J = 7.3 Hz, 2H), 6.39 (d, J = 2.7 Hz, 2H), 4.12 (t, J = 7.0 Hz, 4H), 3.92 (t, J = 6.6 Hz, 4H), 1.95 (s, 6H), 1.72 (d, J = 7.2 Hz, 3H), 1.69 – 1.25 (m, 10H), 1.25 – 0.98 (m, 5H).

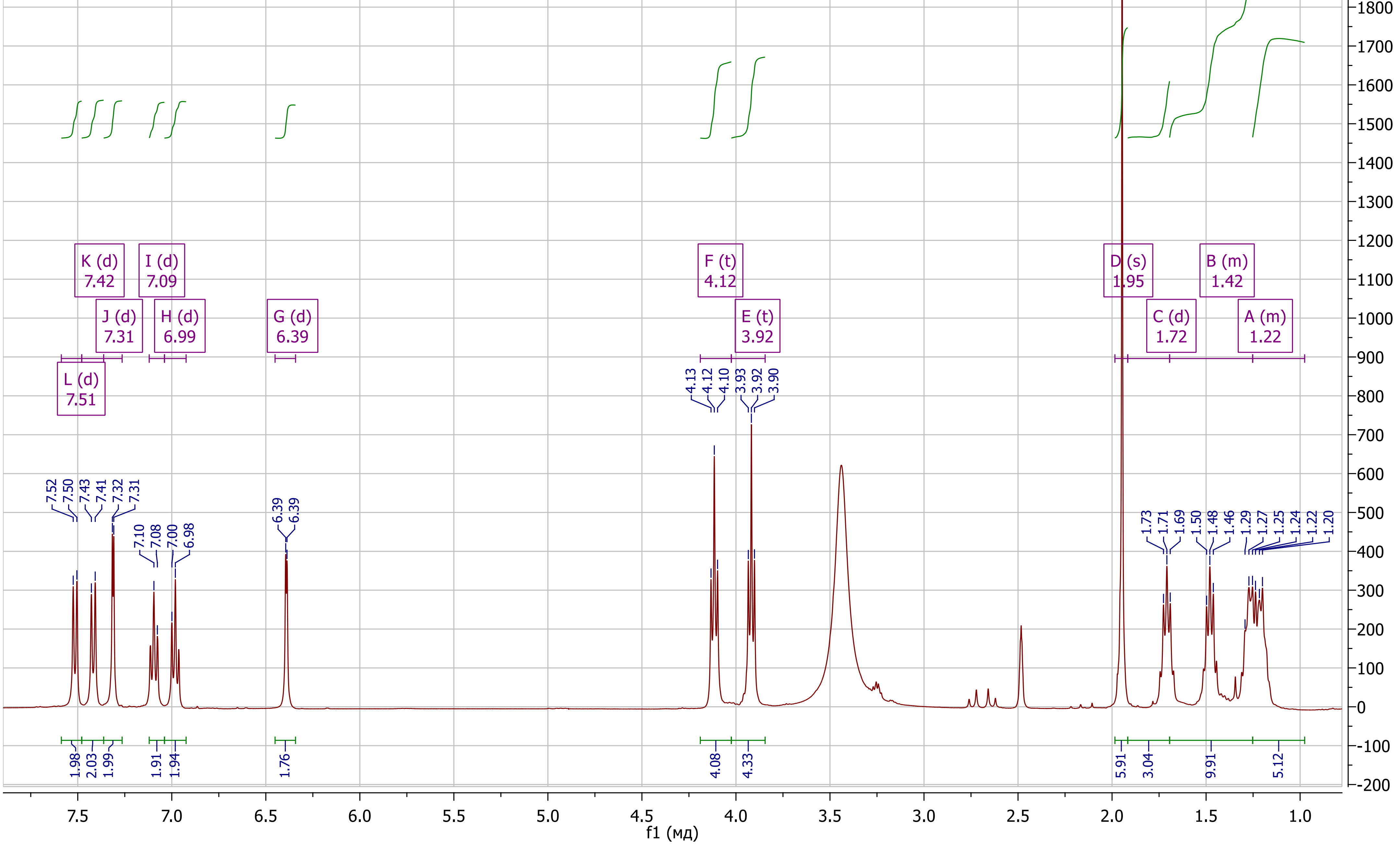

6-(1H-Indol-1-yl)hexyl acetate (4e)

^{13}C NMR (101 MHz, dmso) δ = 170.86, 136.05, 128.95, 128.51, 121.31, 120.82, 119.20, 110.11, 100.75, 64.16, 45.76, 30.15, 28.44, 26.34, 25.45, 21.11.

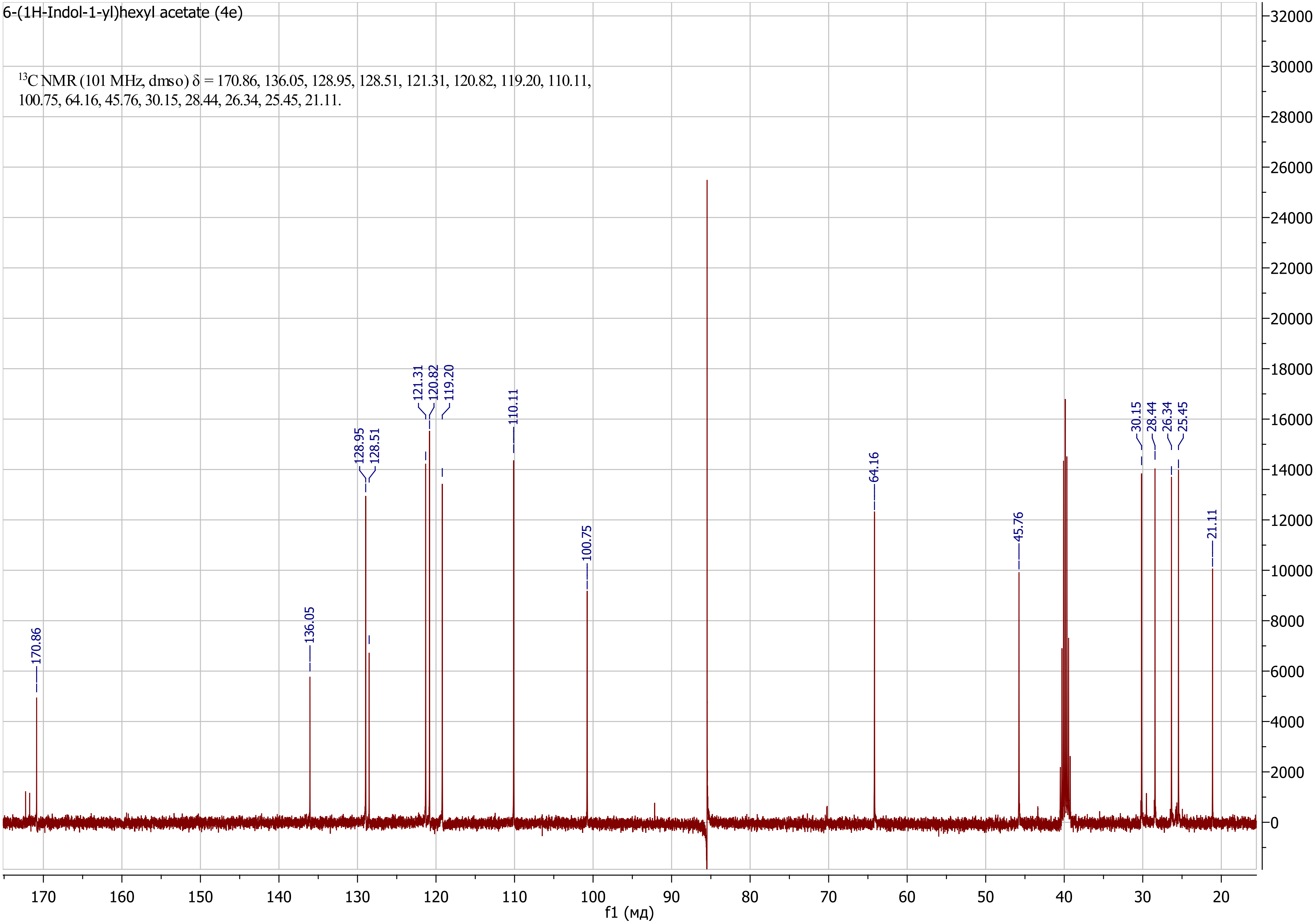

2-(3-Formyl-1H-indol-1-yl)ethyl acetate (5a)

^{1}H NMR (400 MHz, dmso) δ 9.94 (s, 1H), 8.27 (s, 1H), 8.16 (d, *J* = 7.3 Hz, 1H), 7.61 (d, *J* = 7.9 Hz, 1H), 7.28 (ddd, *J* = 11.3, 7.6, 0.8 Hz, 2H), 4.51 (t, *J* = 5.0 Hz, 2H), 4.38 (t, *J* = 5.1 Hz, 2H), 3.57 (s, 2H), 1.88 (s, 3H).

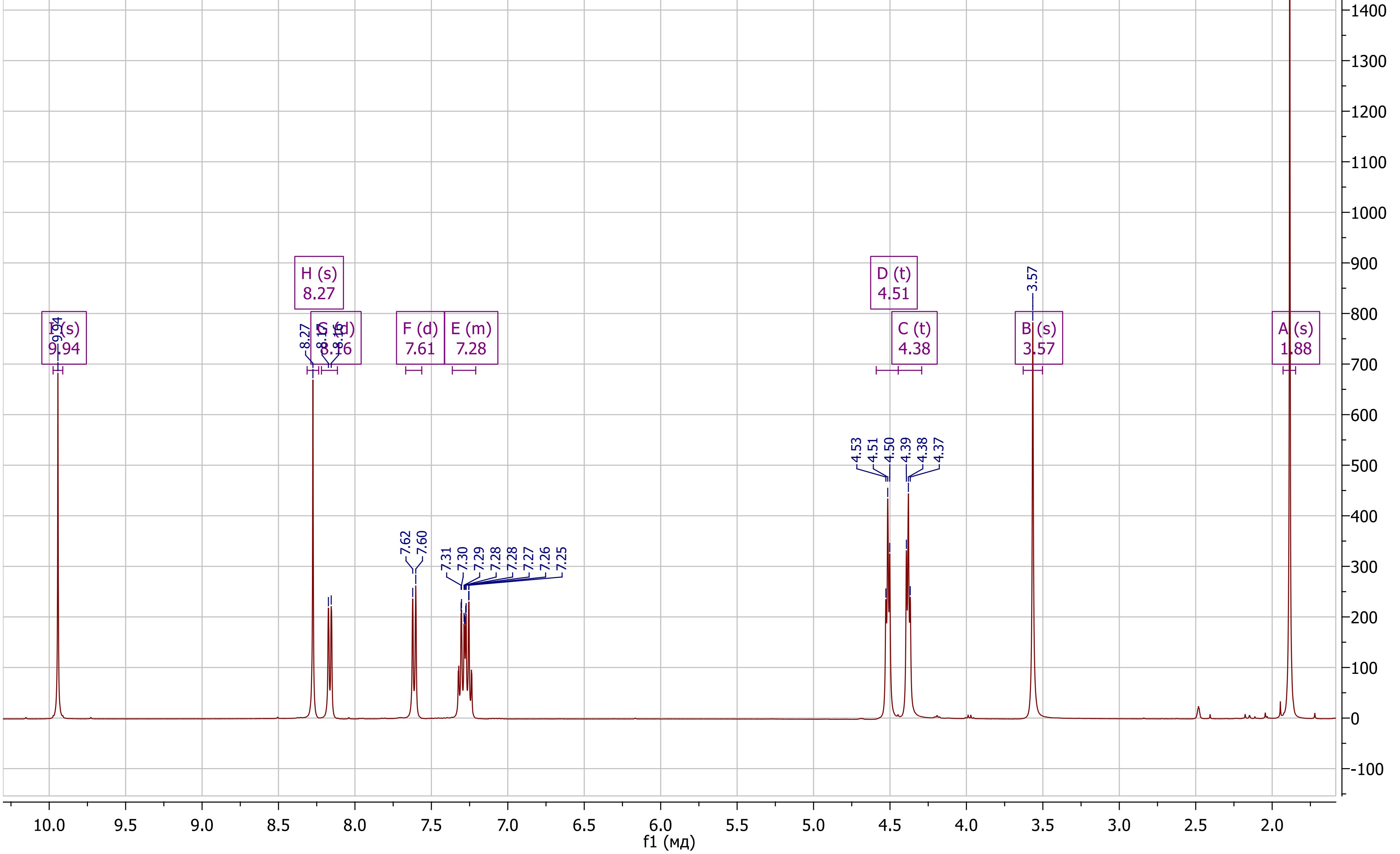

2-(3-Formyl-1H-indol-1-yl)ethyl acetate (5a)

^{13}C NMR (101 MHz, dmso) δ = 185.18, 170.48, 141.46, 137.60, 125.09, 124.04, 122.98, 121.56, 117.98, 111.35, 62.73, 45.81, 20.84.

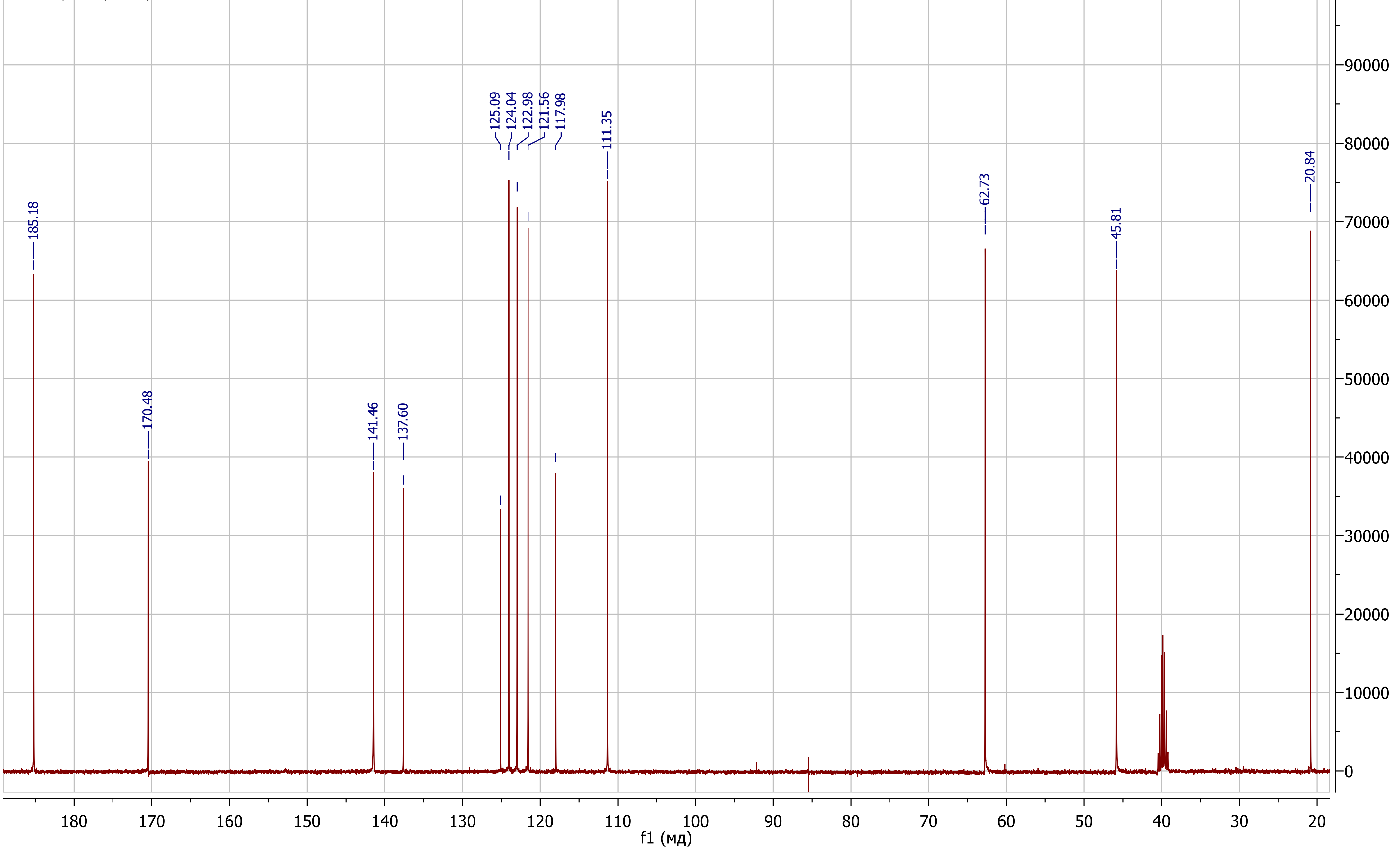

3-(3-Formyl-1H-indol-1-yl)propyl acetate (5b)

^{13}C NMR (101 MHz, dmso) δ = 184.98, 170.77, 170.76, 141.11, 137.43, 125.13, 124.00, 122.92, 121.56, 117.73, 111.29, 61.61, 43.80, 28.75, 20.91.

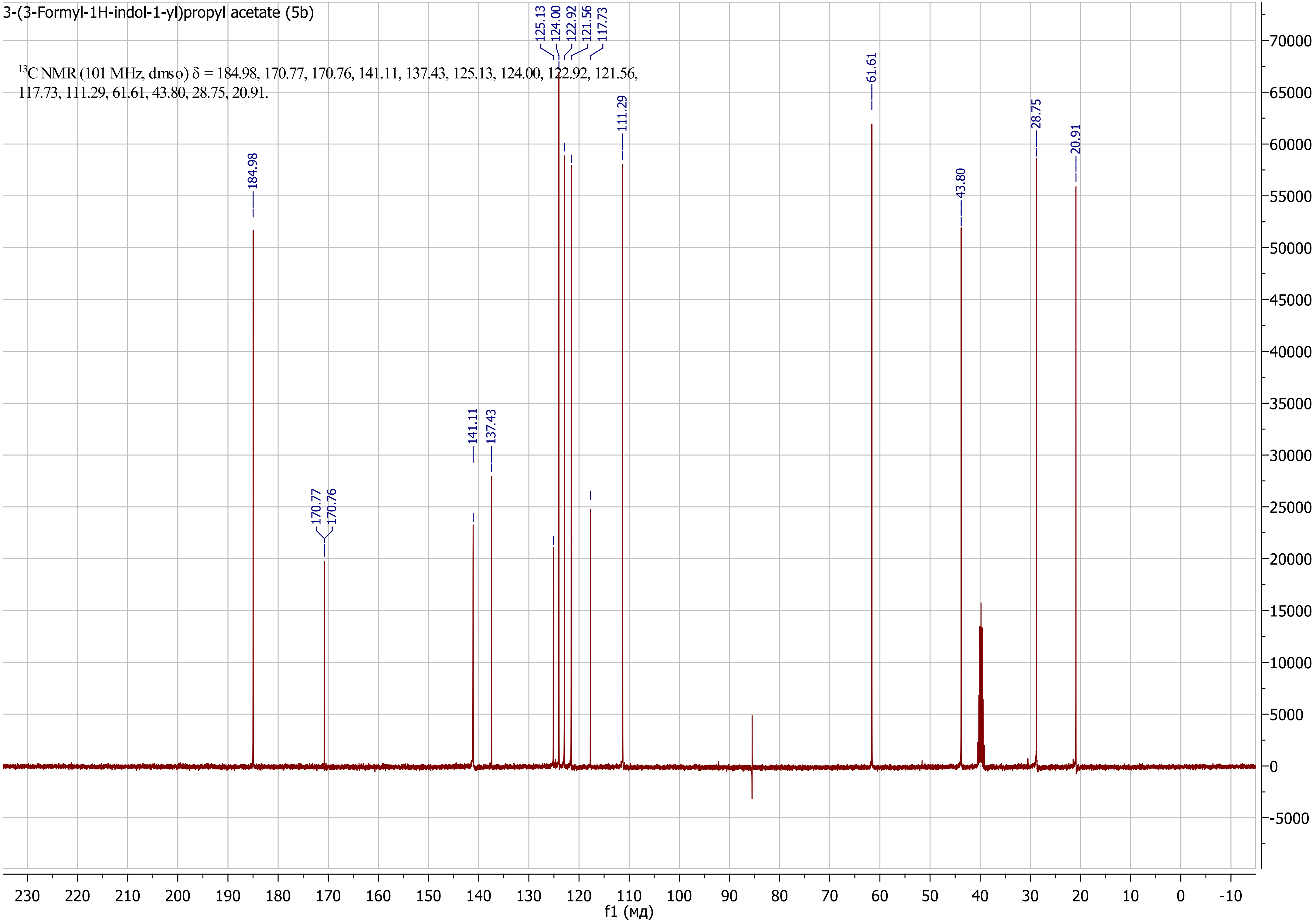

3-(3-Formyl-1H-indol-1-yl)propyl acetate (5b)

^{1}H NMR (400 MHz, dmso) δ 9.91 (s, 1H), 8.27 (s, 1H), 8.15 (d, J = 7.3 Hz, 1H), 7.58 (d, J = 8.0 Hz, 1H), 7.33 – 7.21 (m, J = 11.2, 7.6, 1.0 Hz, 2H), 4.32 (t, J = 6.9 Hz, 2H), 3.96 (t, J = 6.2 Hz, 2H), 2.18 – 2.02 (m, 2H), 1.91 (s, 3H).

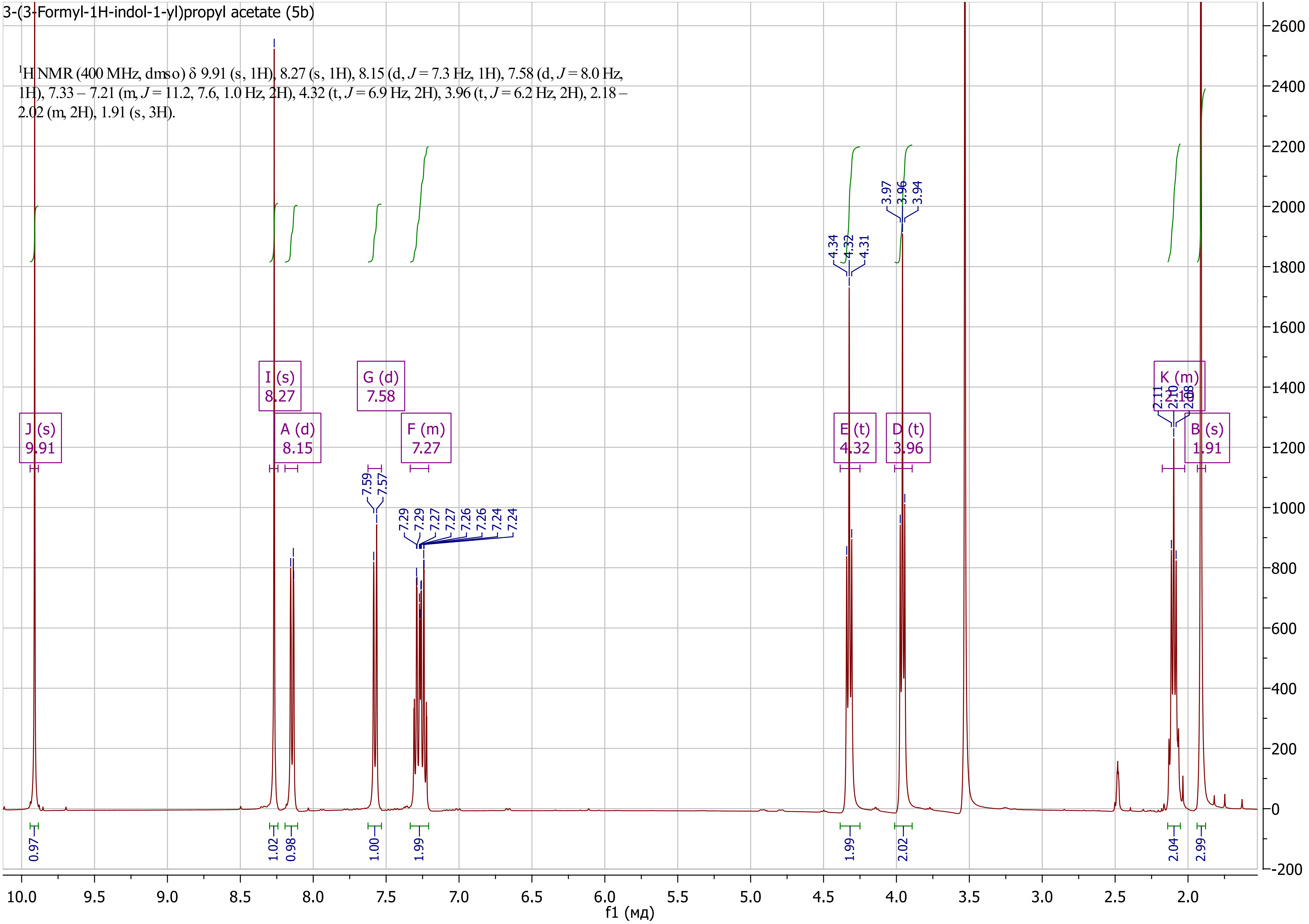

4-(3-Formyl-1H-indol-1-yl)butyl acetate (5c)

^{13}C NMR (101 MHz, dmso) δ = 184.99, 184.96, 170.84, 141.12, 137.41, 125.11, 123.97, 122.91, 121.51, 117.55, 111.46, 85.48, 63.71, 46.32, 26.39, 25.83, 21.08.

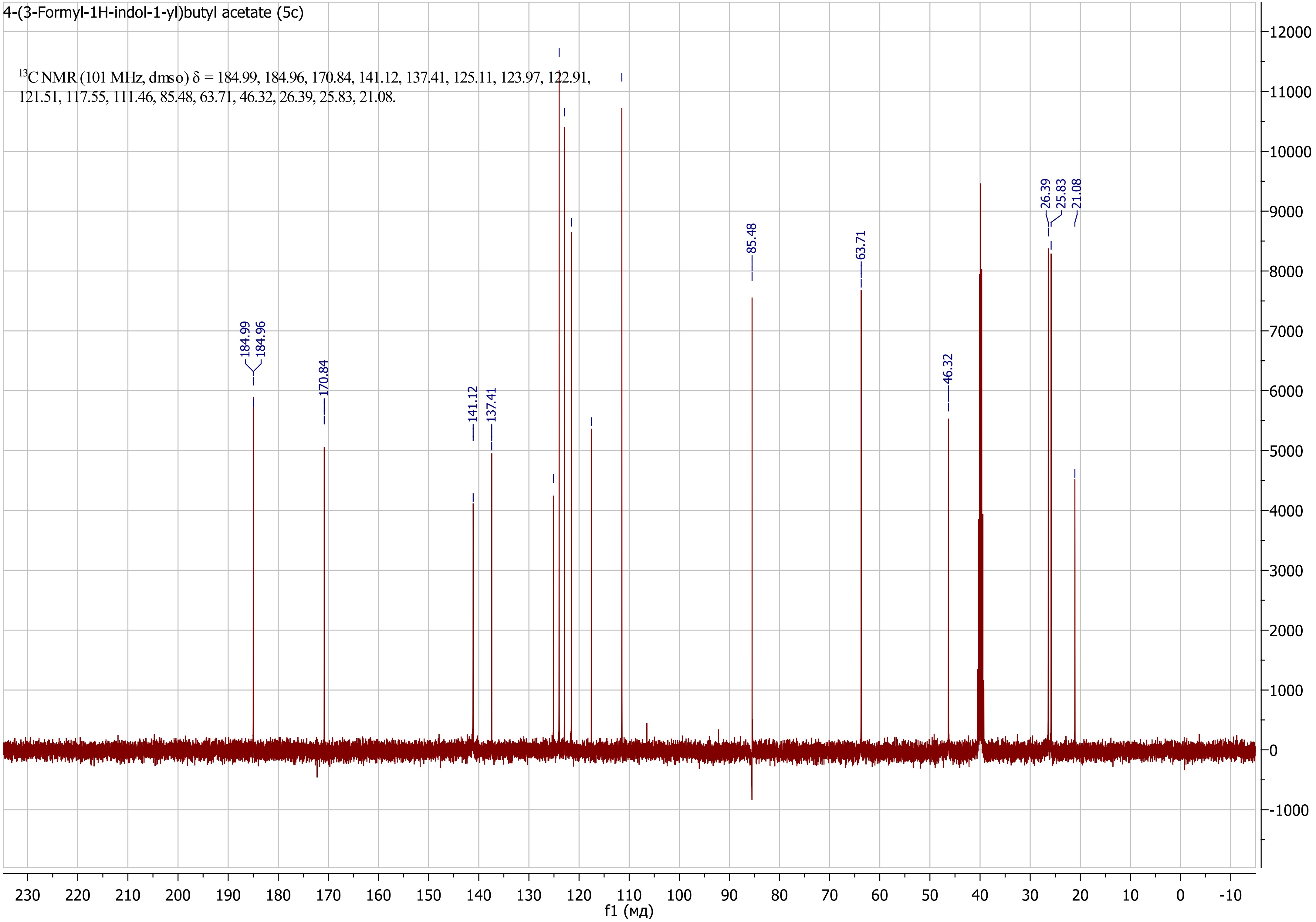

4-(3-Formyl-1H-indol-1-yl)butyl acetate (5c)

^{1}H NMR (400 MHz, dmso) δ 9.89 (s, 1H), 8.30 (s, 1H), 8.10 (d, *J* = 7.5 Hz, 1H), 7.61 (d, *J* = 8.1 Hz, 1H), 7.33 – 7.20 (m, 2H), 4.28 (t, *J* = 7.1 Hz, 2H), 3.98 (t, *J* = 6.6 Hz, 2H), 3.43 (s, 8H), 1.94 (s, 3H), 1.91 – 1.75 (m, 2H), 1.75 – 1.47 (m, 2H).

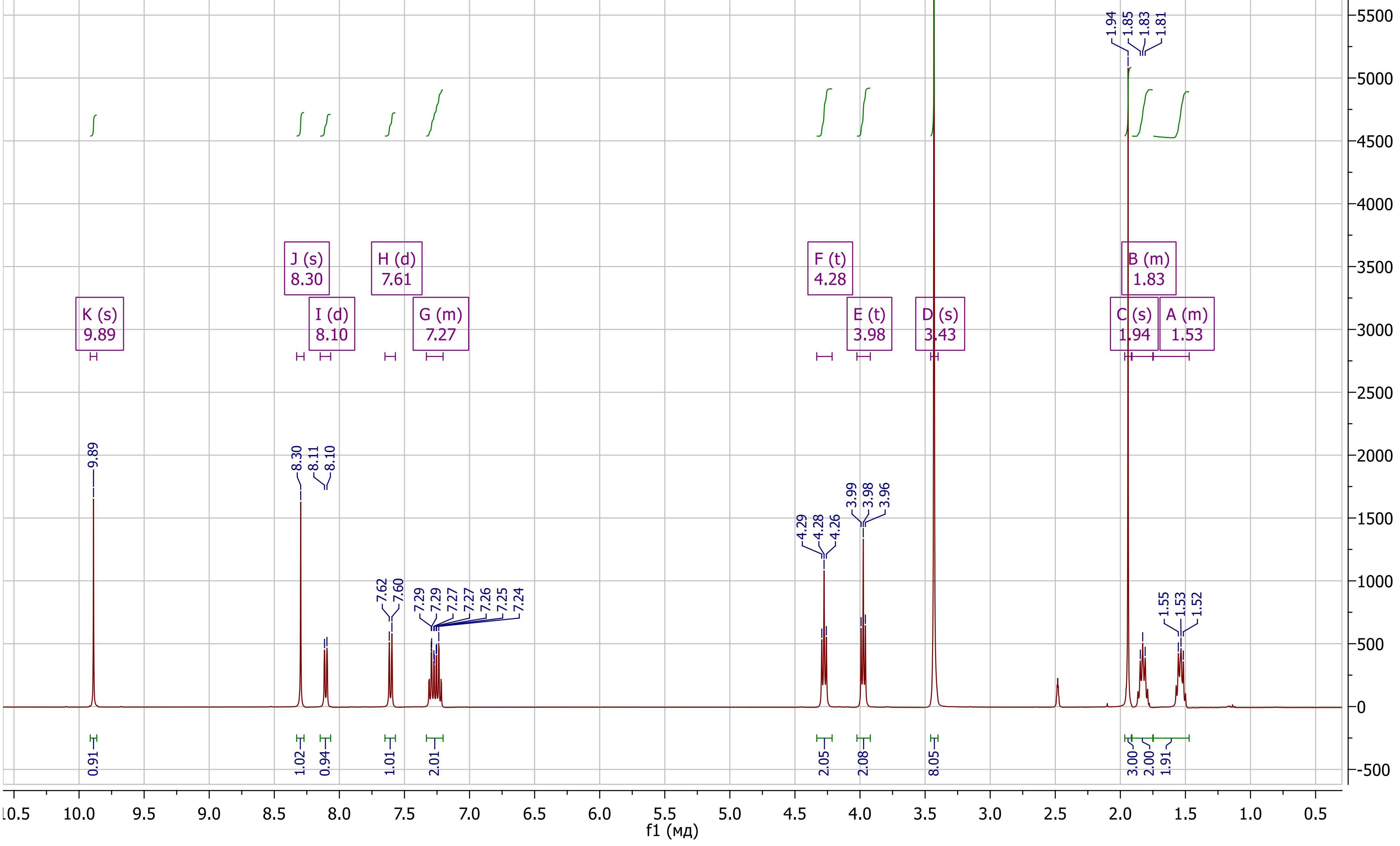

5-(3-Formyl-1H-indol-1-yl)pentyl acetate (5d)

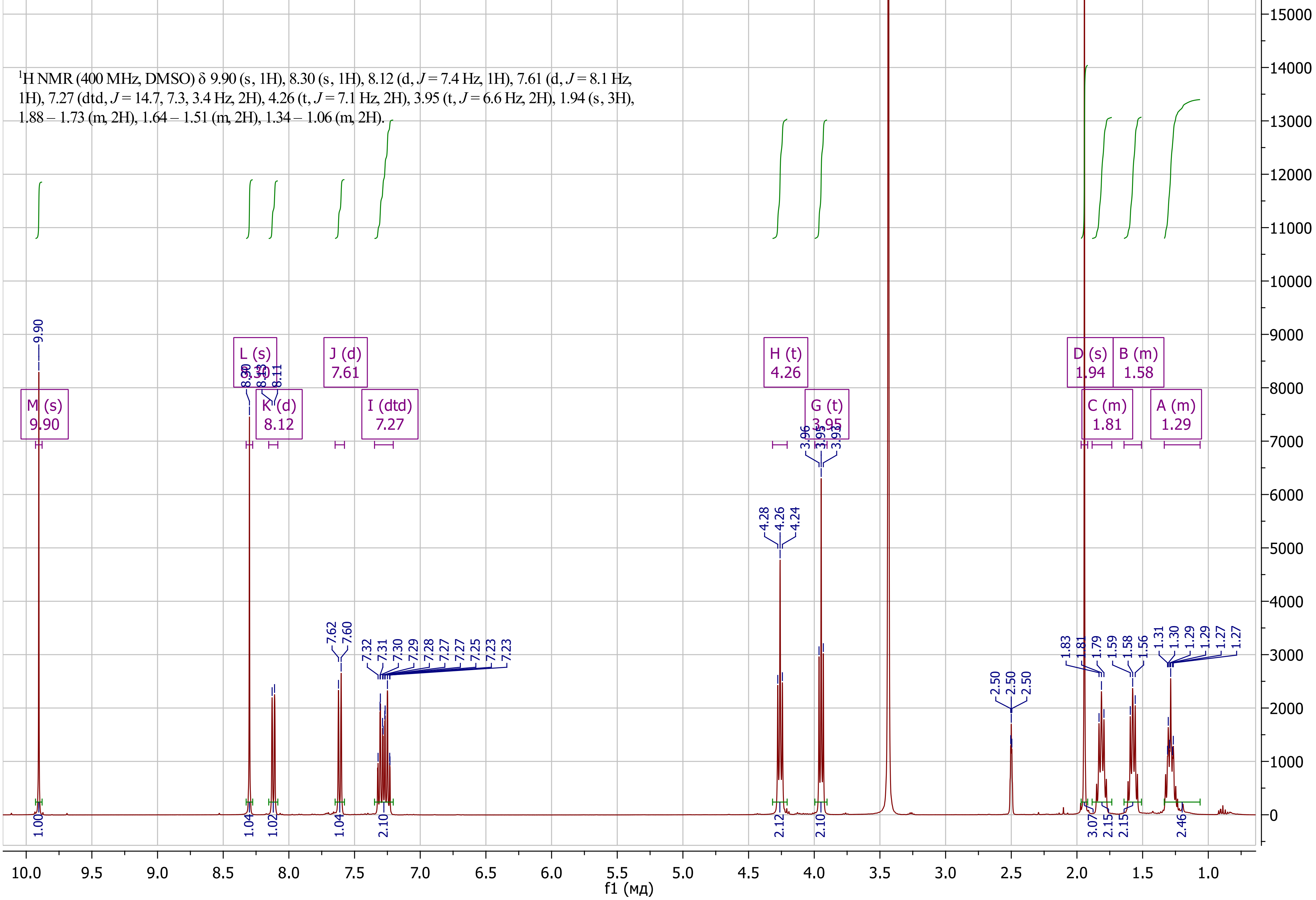

5-(3-Formyl-1H-indol-1-yl)pentyl acetate (5d)

^{13}C NMR (101 MHz, DMSO) δ = 184.57, 170.46, 140.76, 137.06, 124.74, 123.57, 122.50, 121.13, 117.13, 111.09, 63.61, 46.18, 28.94, 27.63, 22.60, 20.70.

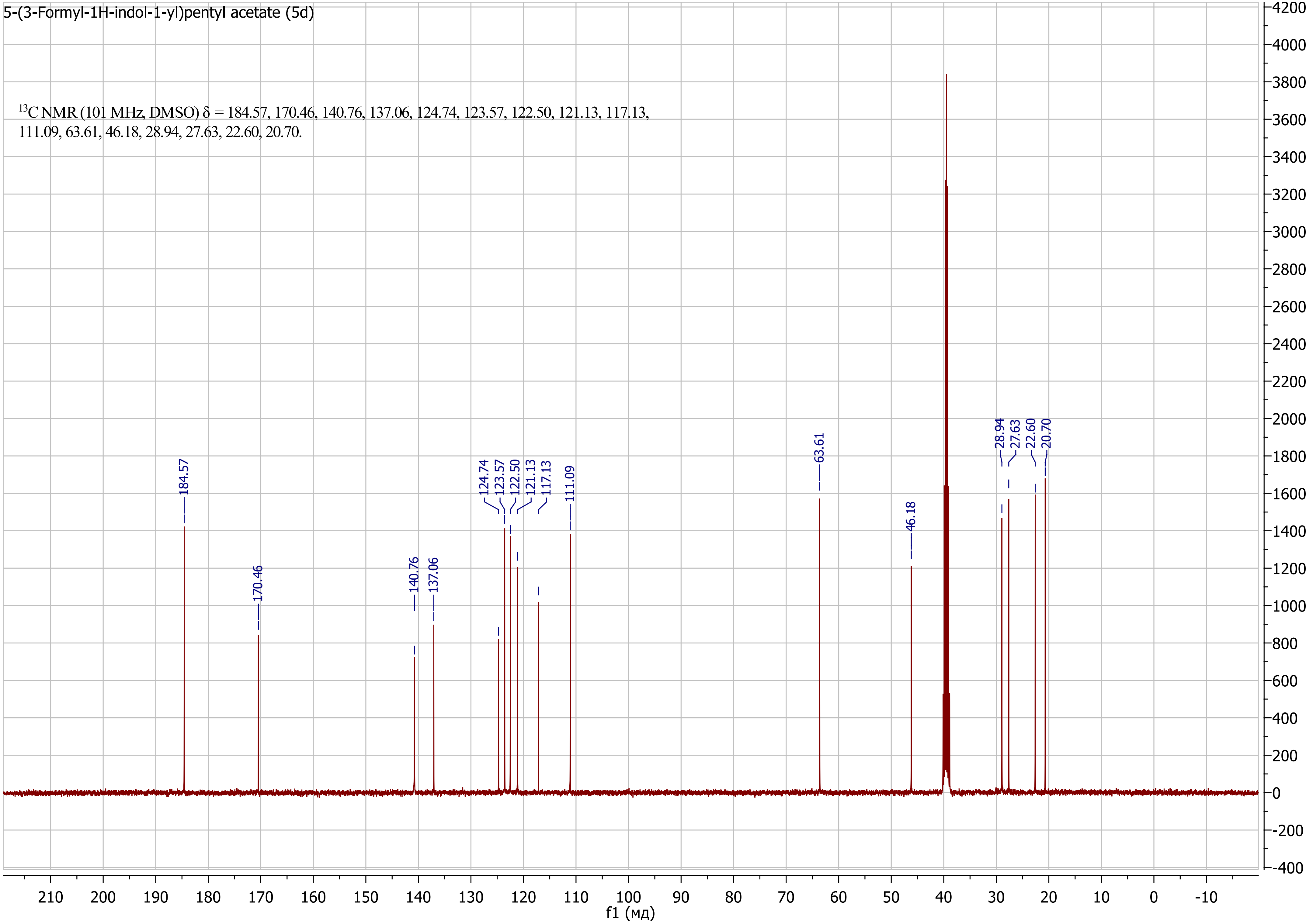

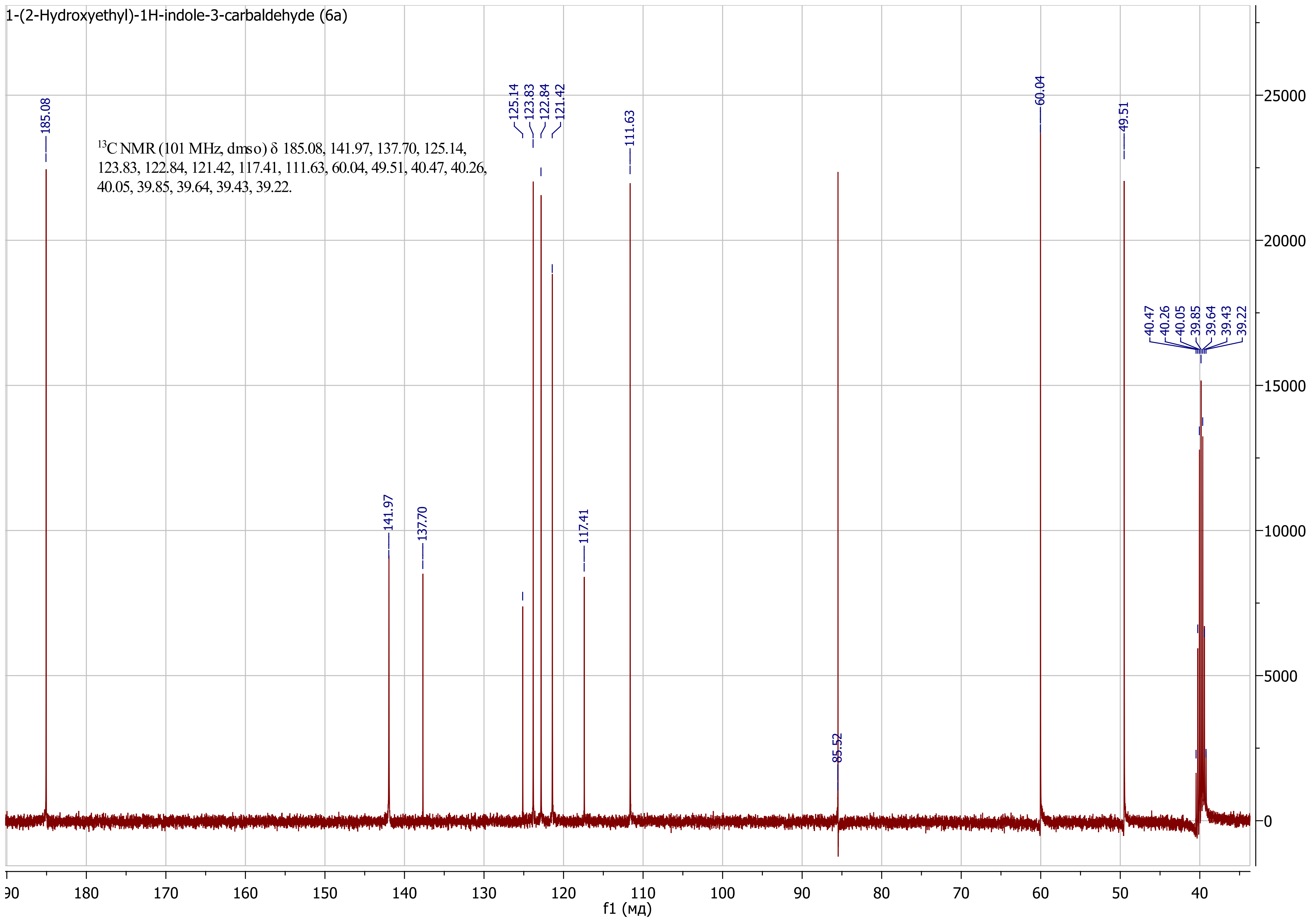
1-(2-Hydroxyethyl)-1H-indole-3-carbaldehyde (6a)
13C NMR (101 MHz, dmso) δ 185.08, 141.97, 137.70, 125.14, 123.83, 122.84, 121.42, 117.41, 111.63, 60.04, 49.51, 40.47, 40.26, 40.05, 39.85, 39.64, 39.43, 39.22.
185.08
141.97
137.70
125.14
123.83
122.84
121.42
117.41
111.63
85.52
60.04
49.51
40.47
40.26
40.05
39.85
39.64
39.43
39.22
f1 (мд)
25000
20000
15000
10000
5000
0

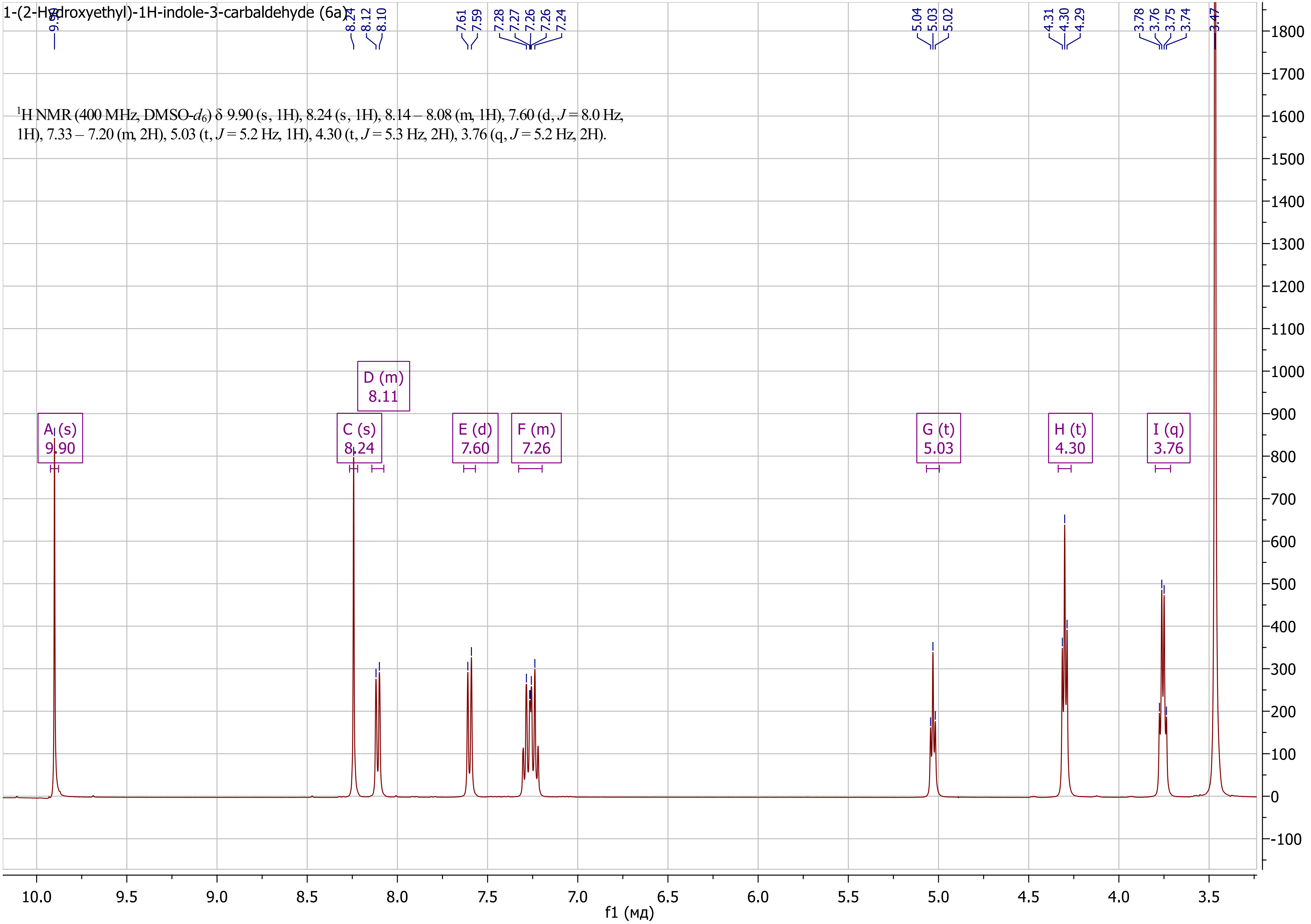
1-(2-Hydroxyethyl)-1H-indole-3-carbaldehyde (6a)
^{1}H NMR (400 MHz, DMSO-d_6) δ 9.90 (s, 1H), 8.24 (s, 1H), 8.14 – 8.08 (m, 1H), 7.60 (d, J = 8.0 Hz, 1H), 7.33 – 7.20 (m, 2H), 5.03 (t, J = 5.2 Hz, 1H), 4.30 (t, J = 5.3 Hz, 2H), 3.76 (q, J = 5.2 Hz, 2H).
9.90
8.24
8.12
8.10
7.61
7.59
7.28
7.27
7.26
7.26
7.24
5.04
5.03
5.02
4.31
4.30
4.29
3.78
3.76
3.75
3.74
3.47
A (s) 9.90
C (s) 8.24
D (m) 8.11
E (d) 7.60
F (m) 7.26
G (t) 5.03
H (t) 4.30
I (q) 3.76
f1 (мд)

1-(3-Hydroxypropyl)-1H-indole-3-carbaldehyde (6b)

^{1}H NMR (400 MHz, dmso) δ 9.89 (s, 1H), 8.26 (s, 1H), 8.10 (d, J = 7.5 Hz, 1H), 7.59 (d, J = 8.1 Hz, 1H), 7.35 – 7.21 (m, 2H), 4.73 (t, J = 5.0 Hz, 1H), 4.32 (t, J = 7.0 Hz, 2H), 3.39 (dd, J = 11.2, 5.9 Hz, 2H), 1.94 (p, J = 6.5 Hz, 2H).

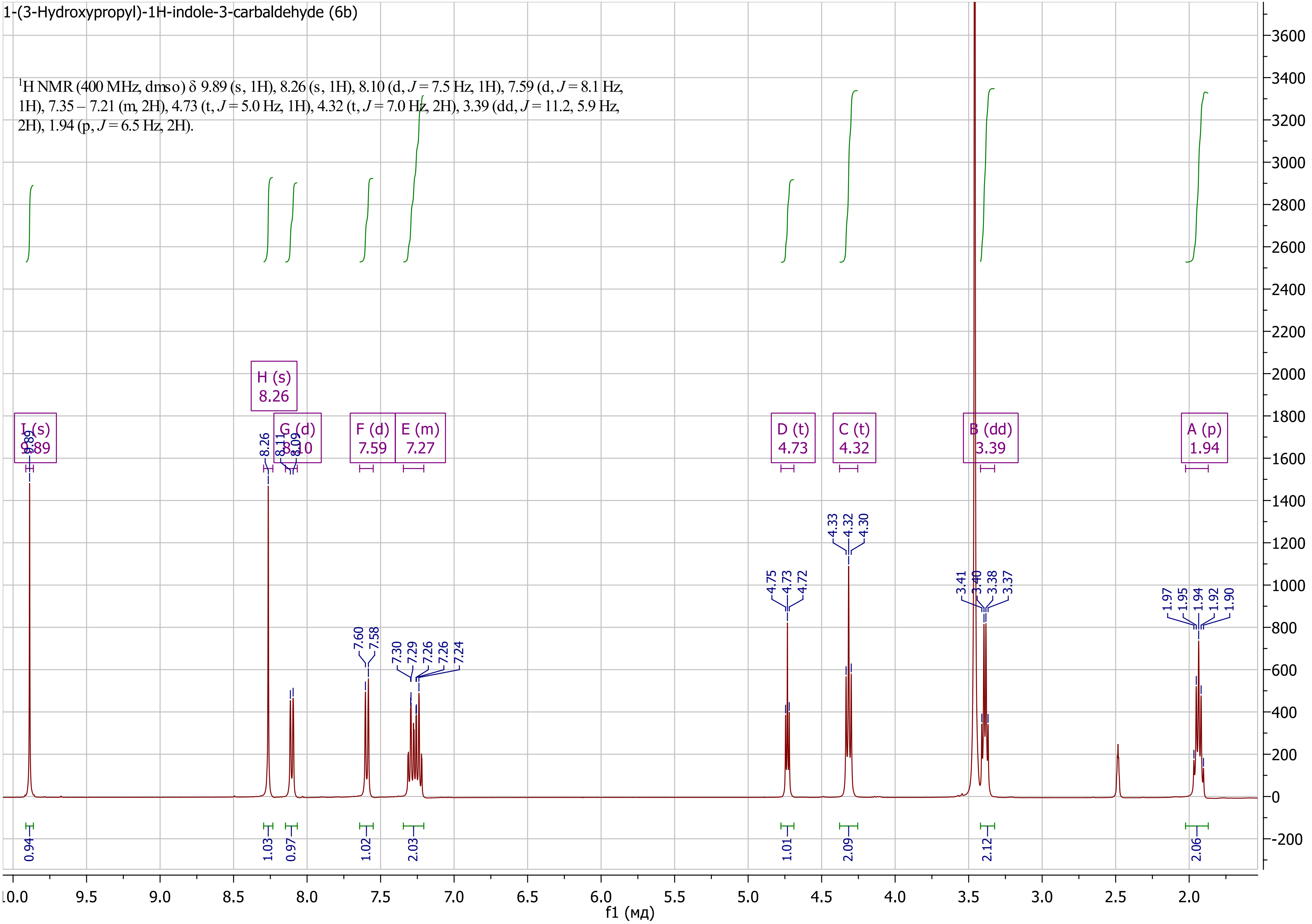

1-(3-Hydroxypropyl)-1H-indole-3-carbaldehyde (6b)

^{13}C NMR (101 MHz, dmso) δ = 184.99, 184.97, 141.29, 137.45, 125.11, 123.96, 122.90, 121.50, 117.48, 111.44, 57.99, 43.82, 32.79.

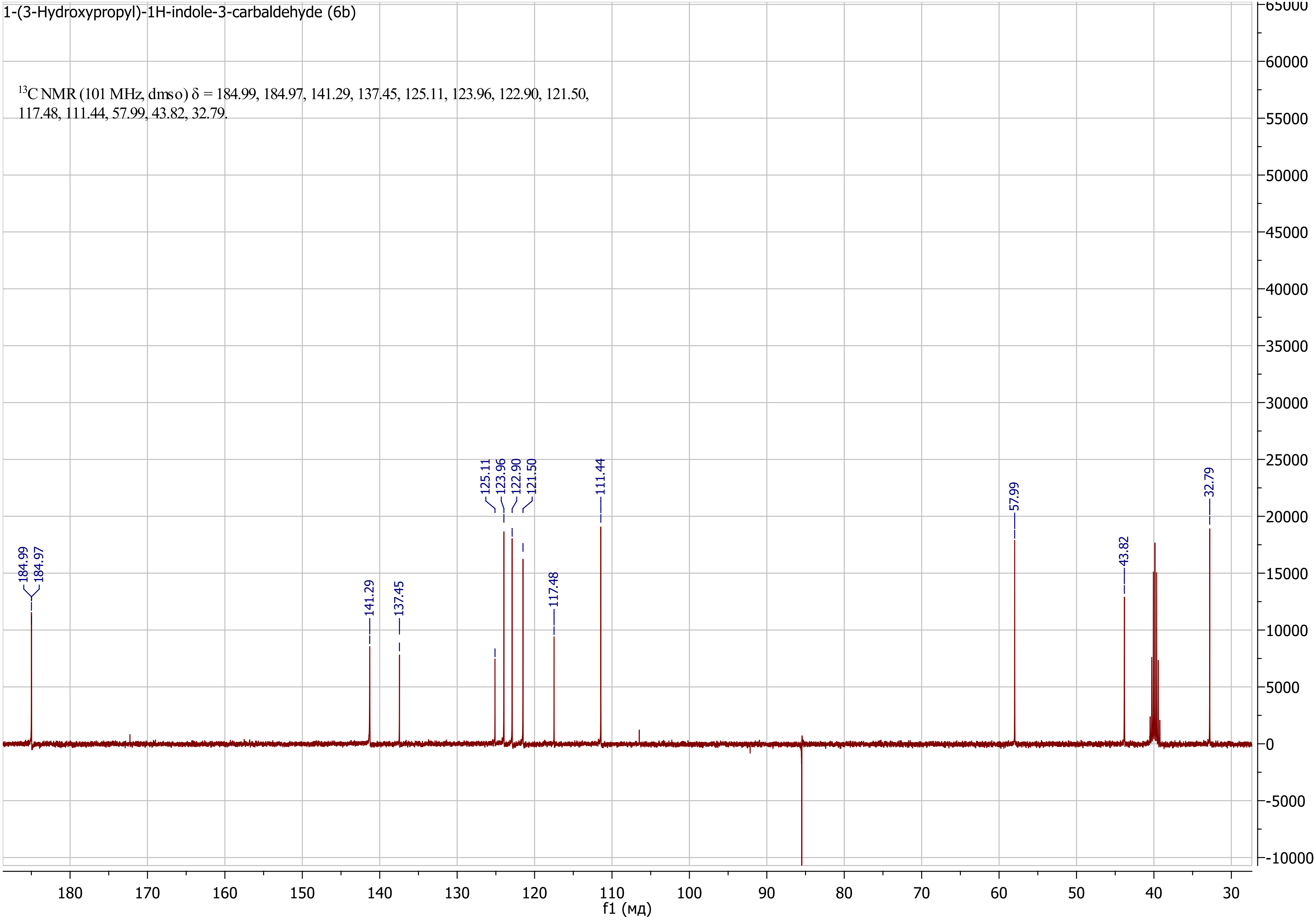

1-(4-Hydroxybutyl)-1H-indole-3-carbaldehyde (6c)

^{1}H NMR (400 MHz, dmso) δ 9.89 (s, 1H), 8.29 (s, 1H), 8.10 (d, *J* = 7.6 Hz, 1H), 7.60 (d, *J* = 8.1 Hz, 1H), 7.33 – 7.21 (m, 2H), 4.47 (t, *J* = 5.1 Hz, 1H), 4.27 (t, *J* = 7.1 Hz, 2H), 1.90 – 1.76 (m, 2H), 1.47 – 1.33 (m, 2H).

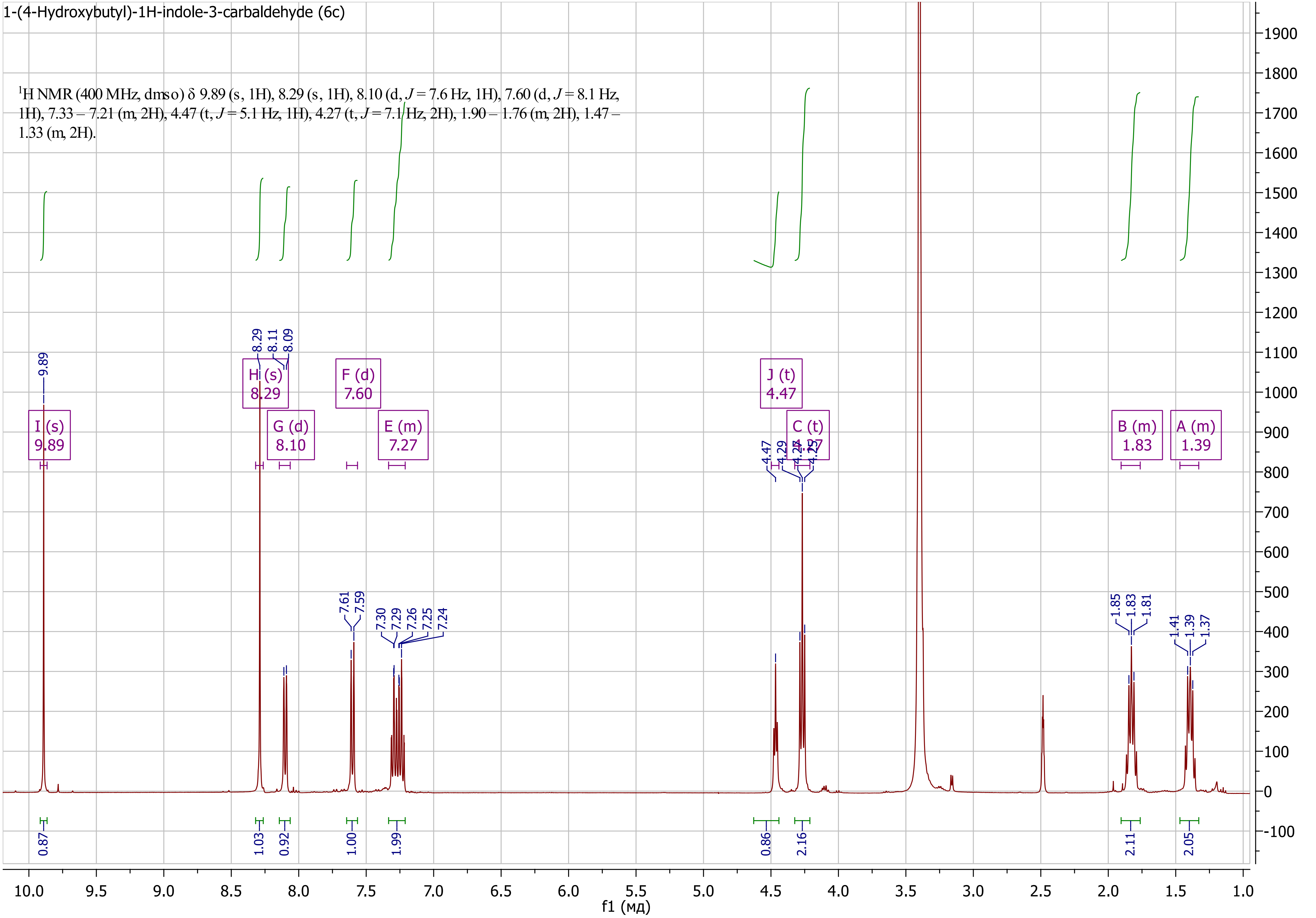

1-(4-Hydroxybutyl)-1H-indole-3-carbaldehyde (6c)

^{13}C NMR (101 MHz, dmso) δ = 184.93, 141.11, 137.46, 125.14, 123.93, 122.86, 121.49, 117.49, 111.49, 60.64, 46.69, 29.94, 26.60.

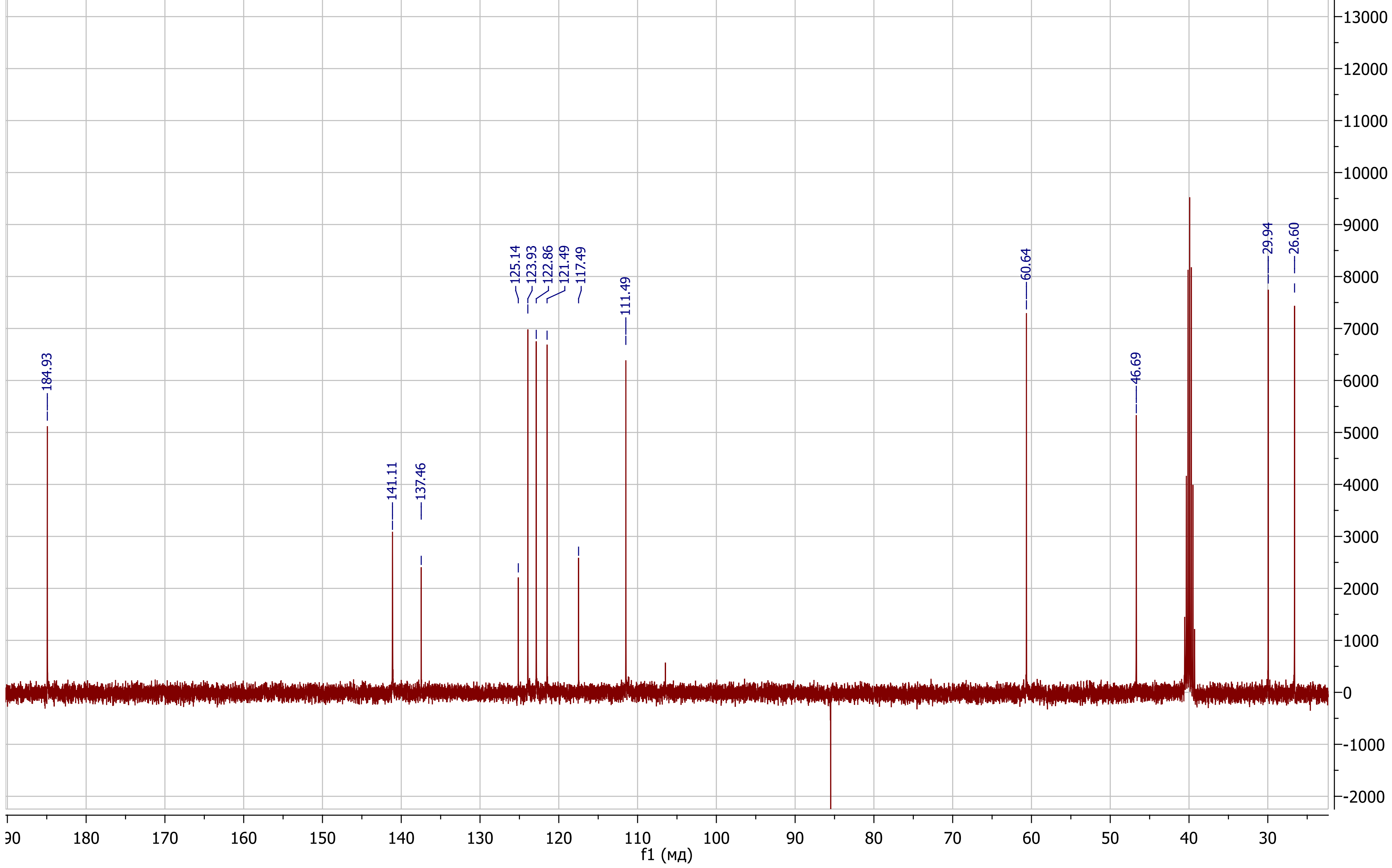

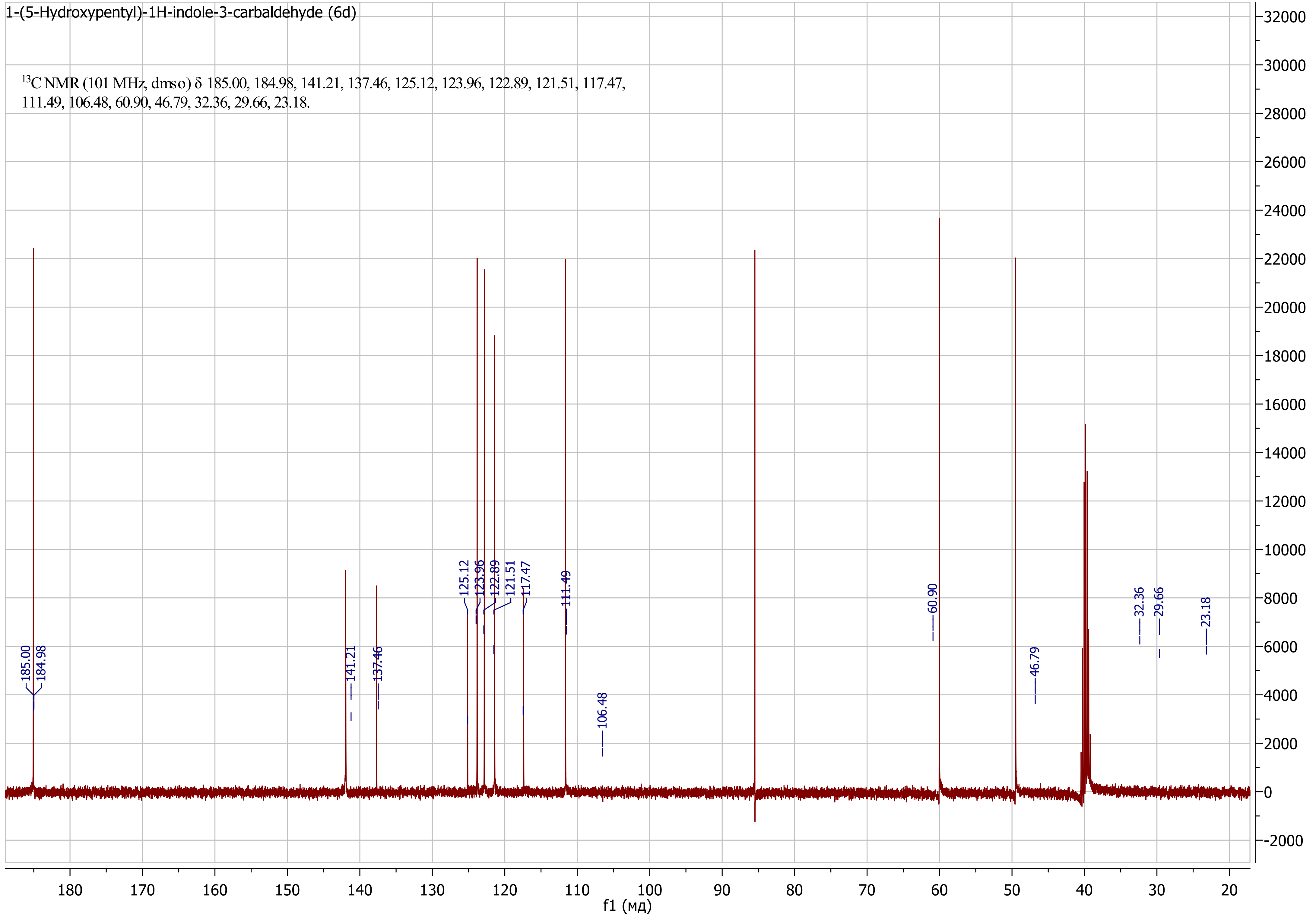

1-(5-Hydroxypentyl)-1H-indole-3-carbaldehyde (6d)
^{13}C NMR (101 MHz, dmso) δ 185.00, 184.98, 141.21, 137.46, 125.12, 123.96, 122.89, 121.51, 117.47, 111.49, 106.48, 60.90, 46.79, 32.36, 29.66, 23.18.
185.00
184.98
141.21
137.46
125.12
123.96
122.89
121.51
117.47
111.49
106.48
60.90
46.79
32.36
29.66
23.18
f1 (мд)

1-(5-Hydroxypentyl)-1H-indole-3-carbaldehyde (6d)

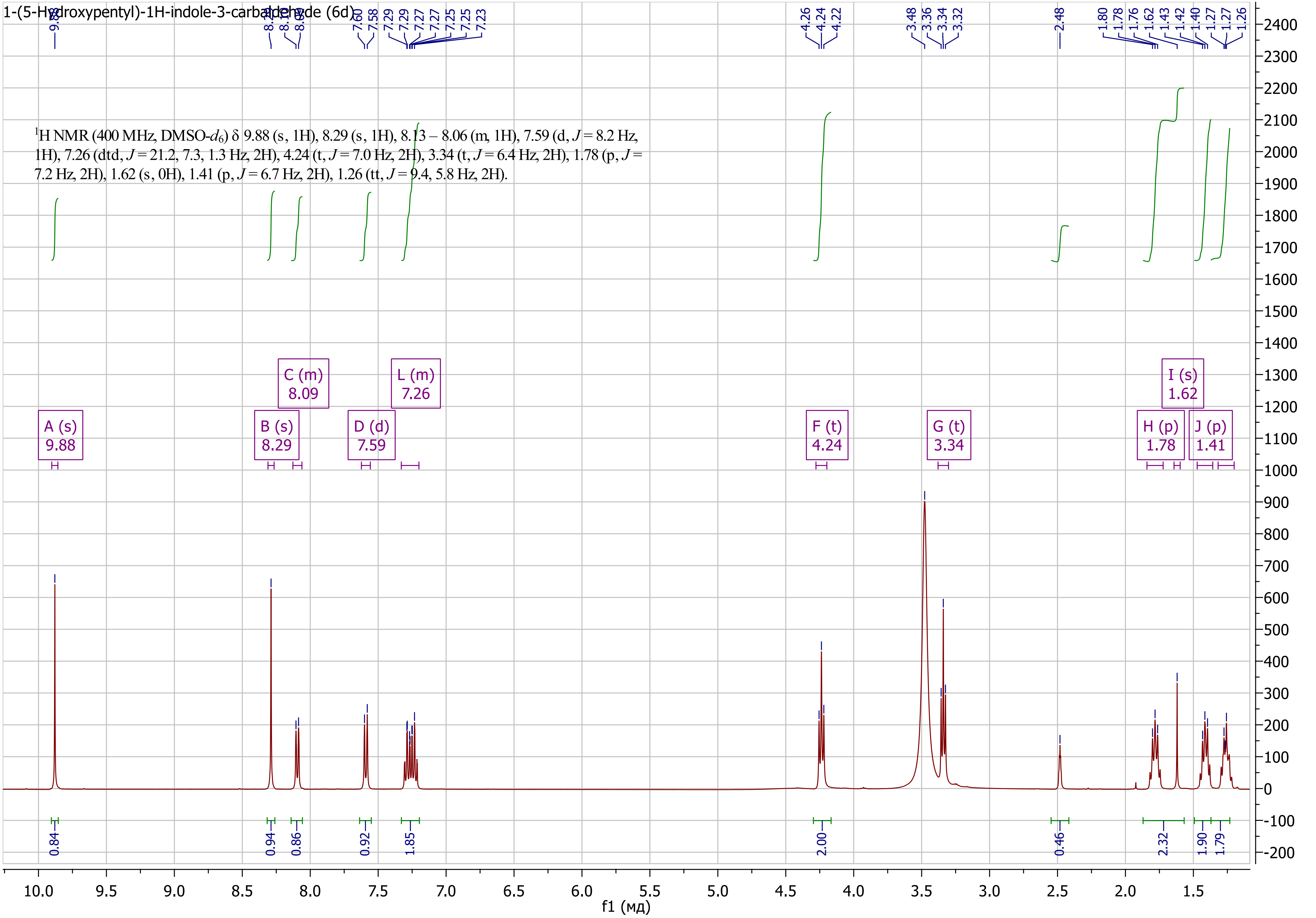

1-(6-Hydroxyhexyl)-1H-indole-3-carbaldehyde (6e)

^{1}H NMR (400 MHz, DMSO-d_6) δ 9.88 (s, 1H), 8.29 (s, 1H), 8.09 (d, J = 7.7 Hz, 1H), 7.59 (d, J = 8.1 Hz, 1H), 7.33 – 7.19 (m, 2H), 4.38 (s, 1H), 4.24 (t, J = 7.1 Hz, 2H), 3.33 (t, J = 6.3 Hz, 2H), 1.77 (p, J = 7.1 Hz, 2H), 1.41 – 1.19 (m, 5H).

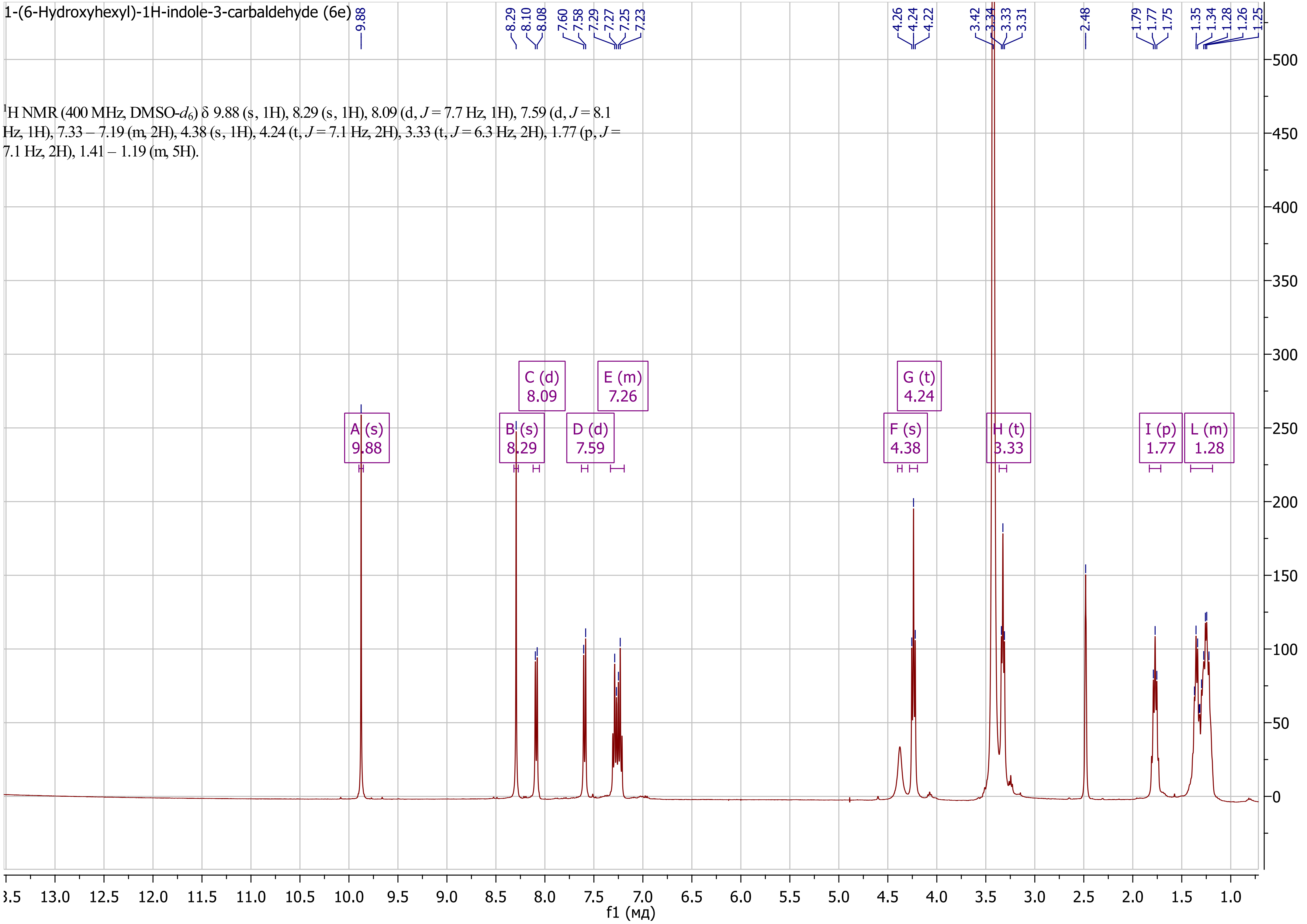

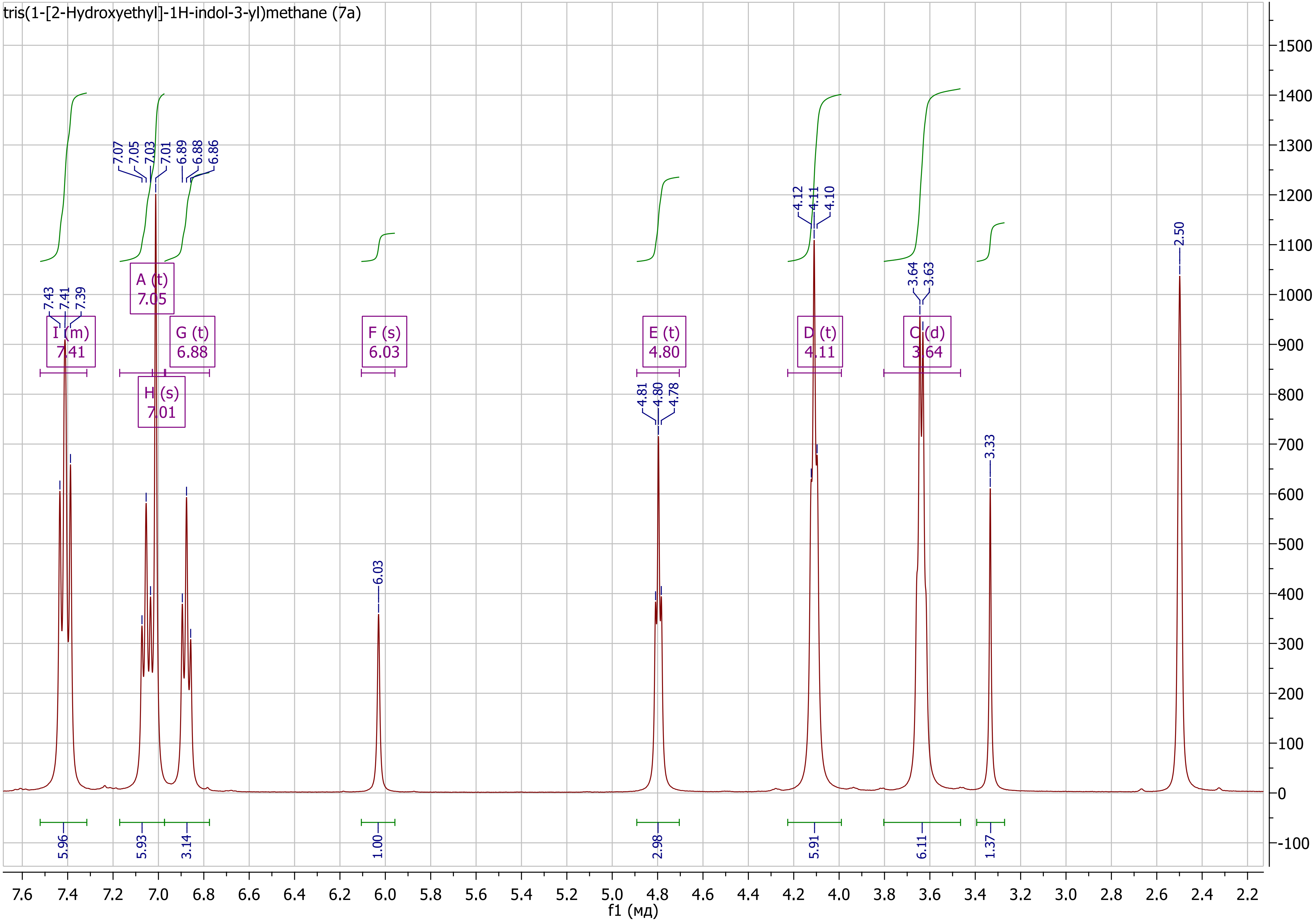
tris(1-[2-Hydroxyethyl]-1H-indol-3-yl)methane (7a)
I (m) 7.41
A (t) 7.05
H (s) 7.01
G (t) 6.88
F (s) 6.03
E (t) 4.80
D (t) 4.11
C (d) 3.64
7.43 7.41 7.39
7.07 7.05 7.03 7.01 6.89 6.88 6.86
6.03
4.81 4.80 4.78
4.12 4.11 4.10
3.64 3.63
3.33
2.50
5.96 5.93 3.14 1.00 2.98 5.91 6.11 1.37
f1 (мд)

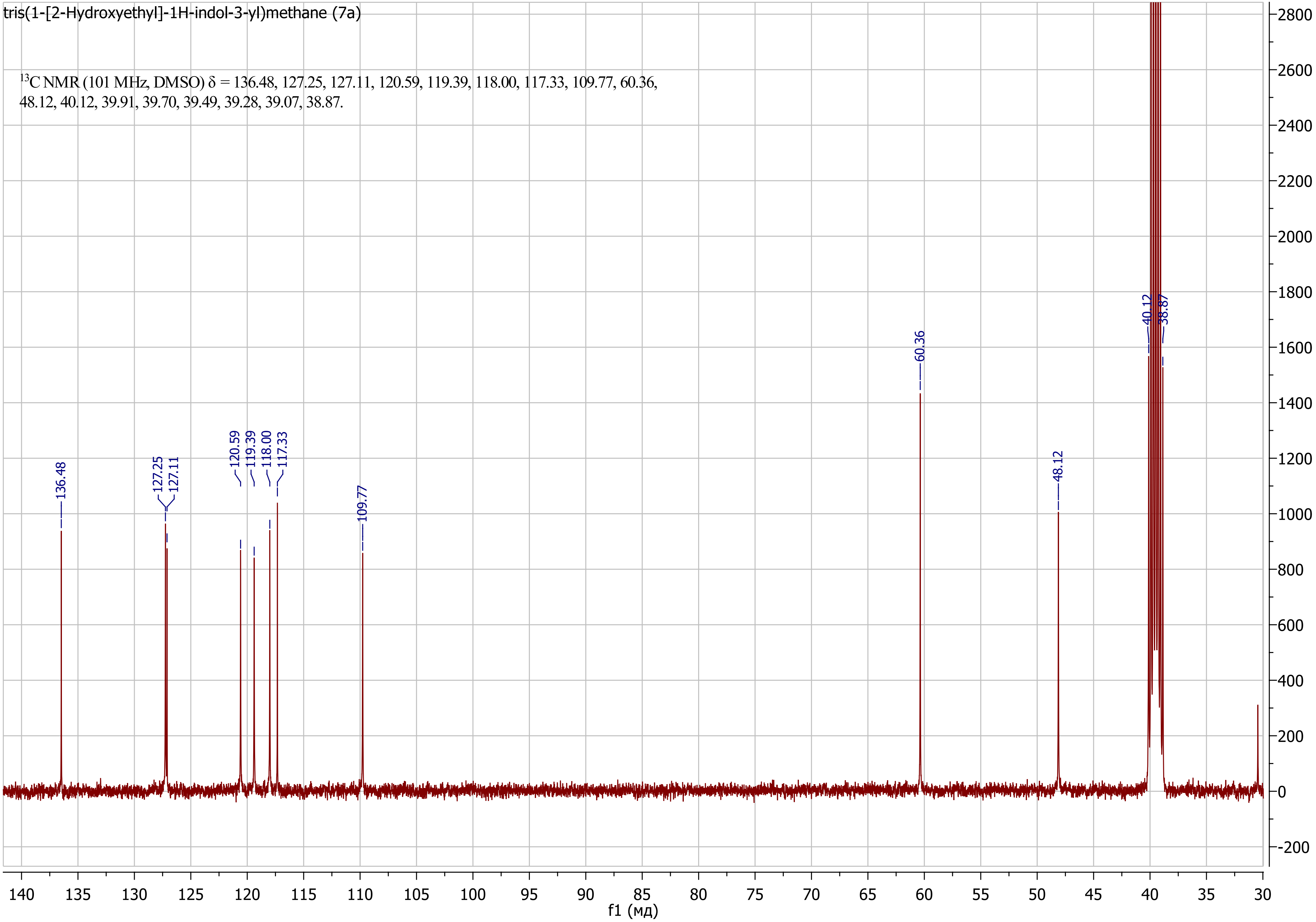

tris(1-[2-Hydroxyethyl]-1H-indol-3-yl)methane (7a)
^{13}C NMR (101 MHz, DMSO) δ = 136.48, 127.25, 127.11, 120.59, 119.39, 118.00, 117.33, 109.77, 60.36, 48.12, 40.12, 39.91, 39.70, 39.49, 39.28, 39.07, 38.87.
136.48
127.25
127.11
120.59
119.39
118.00
117.33
109.77
60.36
48.12
40.12
38.87
f1 (мд)

tris(1-[3-Hydroxypropyl]-1H-indol-3-yl)methane (7b)

^{1}H NMR (400 MHz, DMSO) δ 7.39 (t, J = 7.5 Hz, 6H), 7.05 (d, J = 7.9 Hz, 3H), 6.98 (s, 3H), 6.88 (d, J = 7.7 Hz, 3H), 6.03 (s, 1H), 4.52 (d, J = 5.0 Hz, 3H), 4.13 (t, J = 6.8 Hz, 6H), 3.42 – 3.23 (m, 11H), 1.89 – 1.67 (m, 6H).

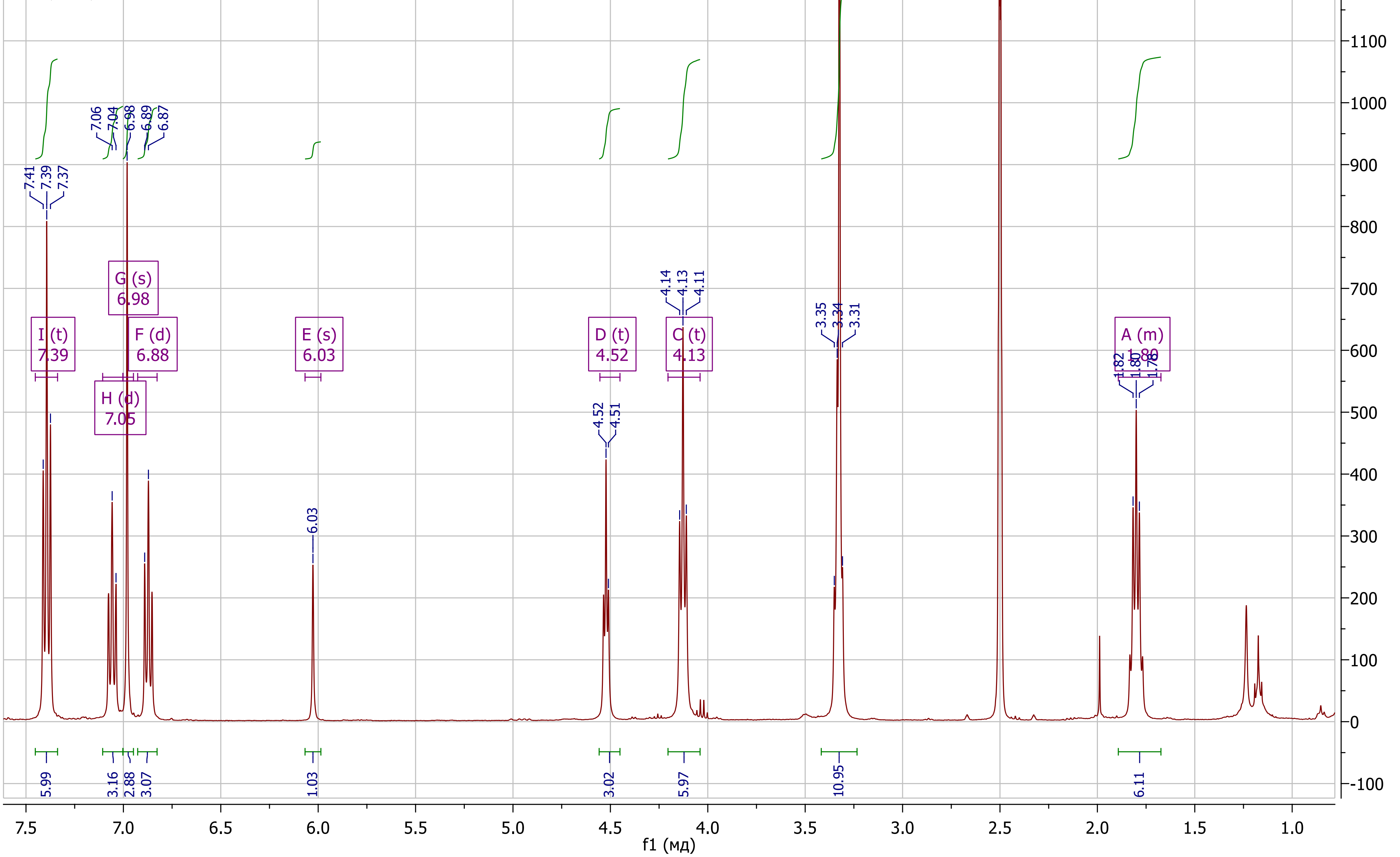

tris(1-[3-Hydroxypropyl]-1H-indol-3-yl)methane (7b)

^{13}C NMR (101 MHz, DMSO) δ = 136.20, 127.05, 126.69, 120.71, 119.55, 117.99, 117.24, 109.63, 57.79, 42.20, 40.12, 39.92, 39.71, 39.50, 39.29, 39.08, 38.87, 33.03.

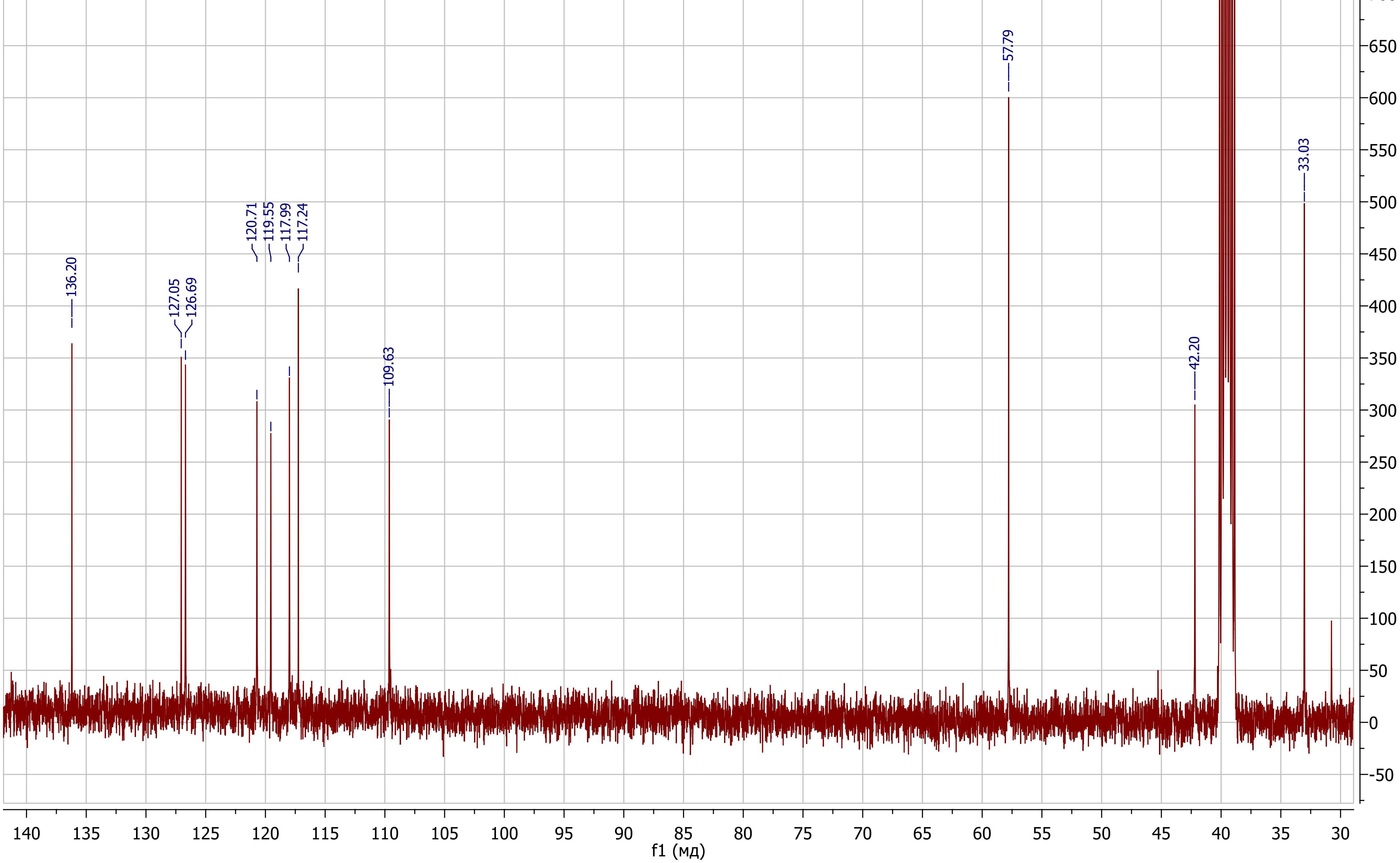

tris(1-[4-Hydroxybutyl]-1H-indol-3-yl)methane (7c)

^{1}H NMR (400 MHz, DMSO) δ 7.40 (d, J = 2.8 Hz, 3H), 7.38 (d, J = 3.3 Hz, 3H), 7.05 (t, J = 7.5 Hz, 3H), 6.96 (s, 3H), 6.86 (t, J = 7.4 Hz, 3H), 6.03 (s, 1H), 4.44 (t, J = 5.0 Hz, 3H), 4.07 (t, J = 6.8 Hz, 6H), 3.35 (dd, J = 11.5, 6.1 Hz, 7H), 1.90 – 1.48 (m, 6H), 1.48 – 1.11 (m, 6H).

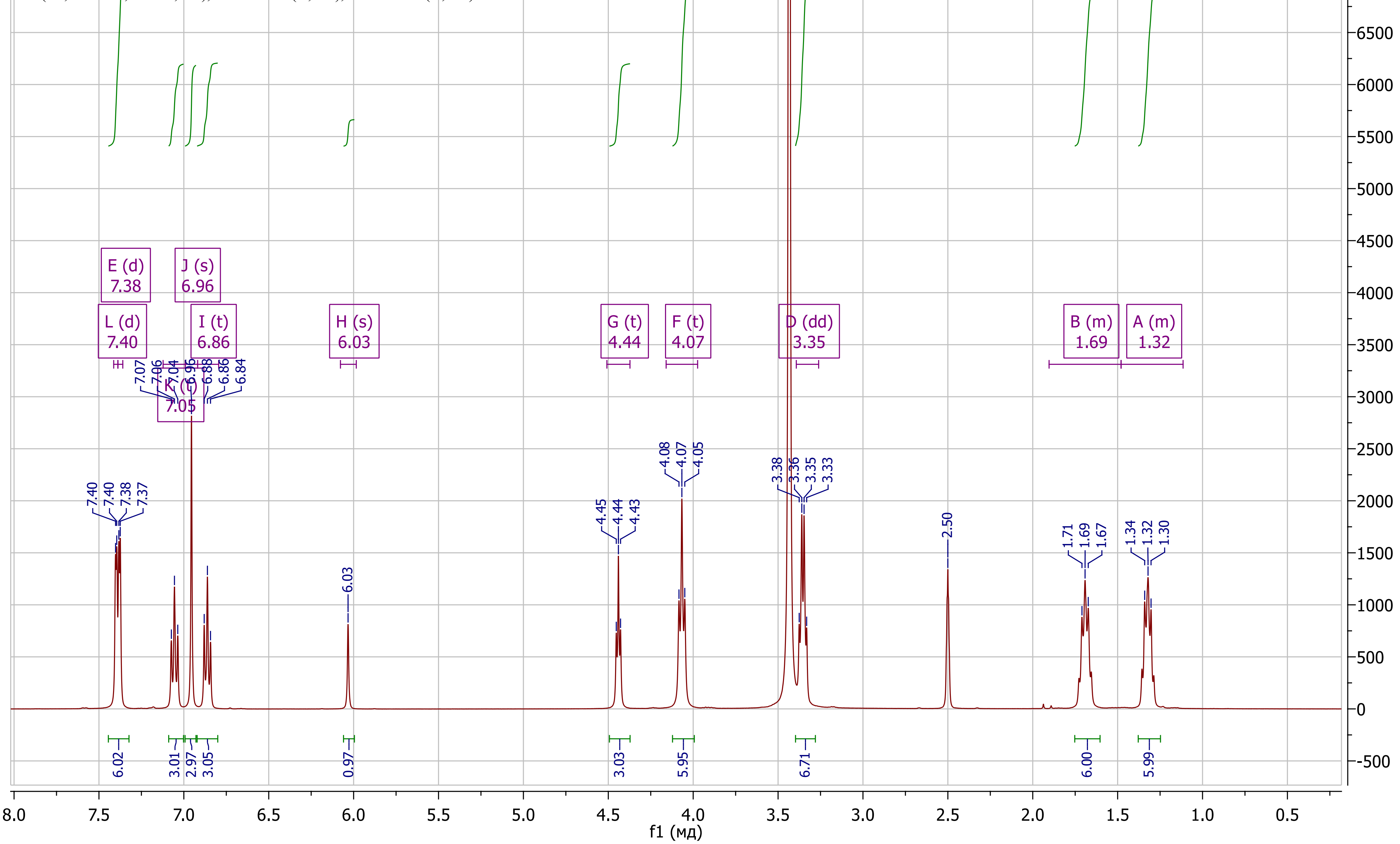

tris(1-[4-Hydroxybutyl]-1H-indol-3-yl)methane (7c)

^{13}C NMR (101 MHz, DMSO) δ = 136.31, 127.15, 126.74, 120.80, 119.68, 118.06, 117.30, 109.77, 60.35, 45.24, 30.87, 29.70, 26.65.

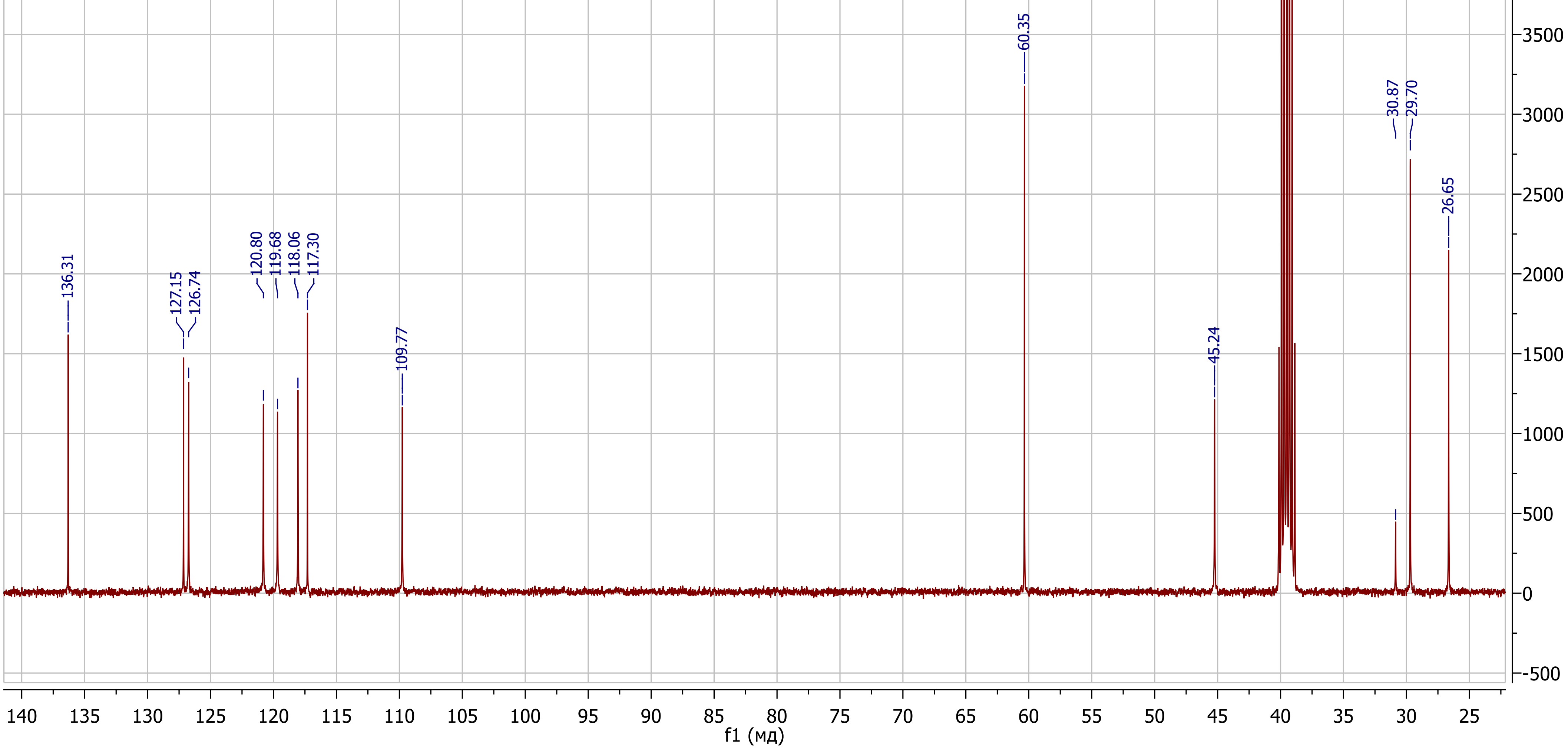

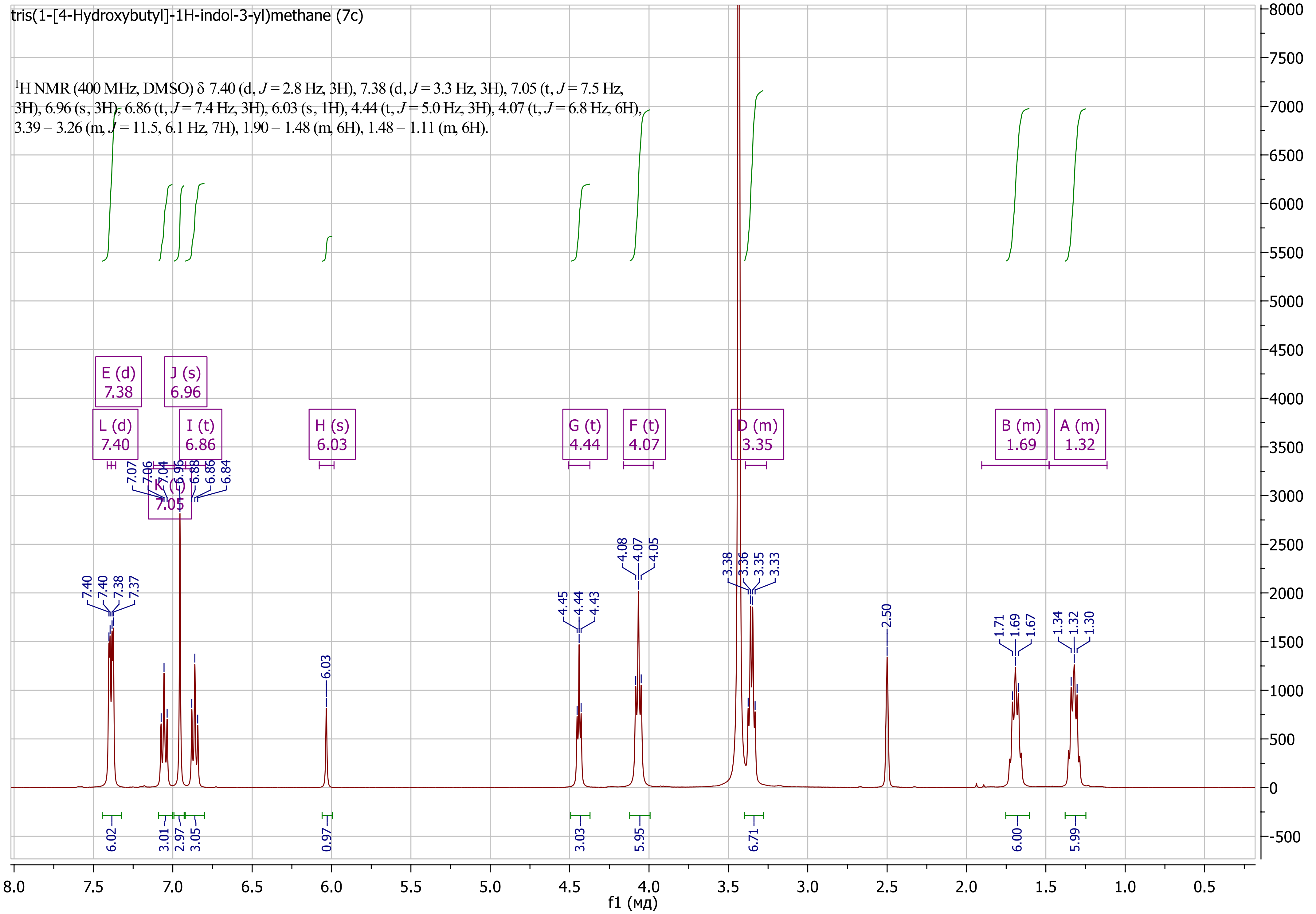
tris(1-[4-Hydroxybutyl]-1H-indol-3-yl)methane (7c)
¹H NMR (400 MHz, DMSO) δ 7.40 (d, J = 2.8 Hz, 3H), 7.38 (d, J = 3.3 Hz, 3H), 7.05 (t, J = 7.5 Hz, 3H), 6.96 (s, 3H), 6.86 (t, J = 7.4 Hz, 3H), 6.03 (s, 1H), 4.44 (t, J = 5.0 Hz, 3H), 4.07 (t, J = 6.8 Hz, 6H), 3.39 – 3.26 (m, J = 11.5, 6.1 Hz, 7H), 1.90 – 1.48 (m, 6H), 1.48 – 1.11 (m, 6H).
E (d) 7.38
J (s) 6.96
L (d) 7.40
I (t) 6.86
K (t) 7.05
H (s) 6.03
G (t) 4.44
F (t) 4.07
D (m) 3.35
B (m) 1.69
A (m) 1.32
6.02
3.01
2.97
3.05
0.97
3.03
5.95
6.71
6.00
5.99
f1 (мд)

tris(1-[4-Hydroxybutyl]-1H-indol-3-yl)methane (7c)

^{13}C NMR (101 MHz, DMSO) δ = 136.31, 127.15, 126.74, 120.80, 119.68, 118.06, 117.30, 109.77, 60.35, 45.24, 30.87, 29.70, 26.65.

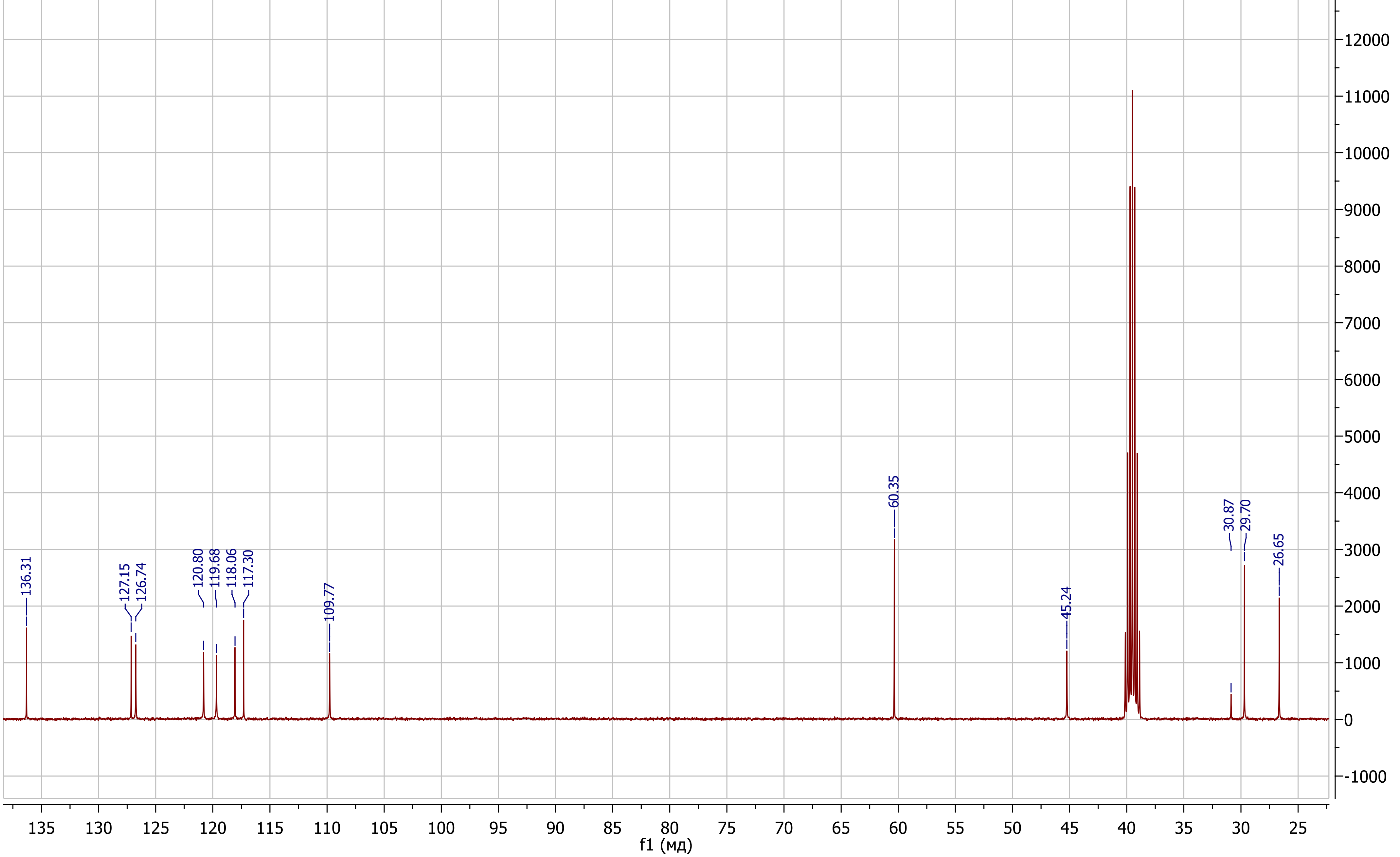

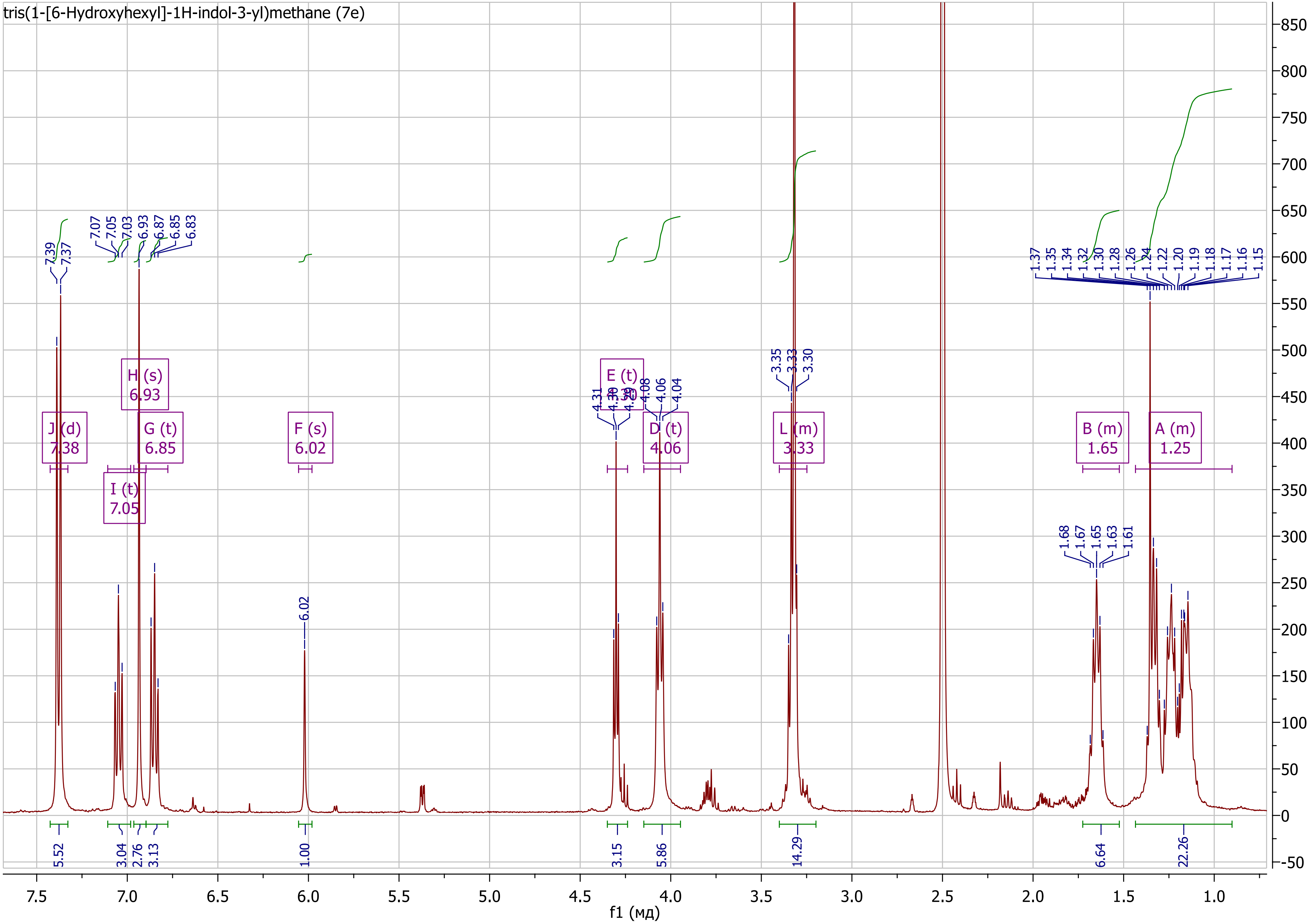

tris(1-[6-Hydroxyhexyl]-1H-indol-3-yl)methane (7e)
J (d) 7.38
I (t) 7.05
H (s) 6.93
G (t) 6.85
F (s) 6.02
E (t)
D (t) 4.06
L (m) 3.33
B (m) 1.65
A (m) 1.25
5.52
3.04
2.76
3.13
1.00
3.15
5.86
14.29
6.64
22.26
f1 (мд)

tris(1-[6-Hydroxyhexyl]-1H-indol-3-yl)methane (7e)

^{13}C NMR (101 MHz, DMSO) δ = 136.18, 127.03, 126.67, 120.67, 119.56, 117.89, 117.09, 109.63, 60.54, 45.15, 40.11, 39.90, 39.69, 39.48, 39.28, 39.07, 38.86, 32.43, 29.80, 26.07, 25.08.

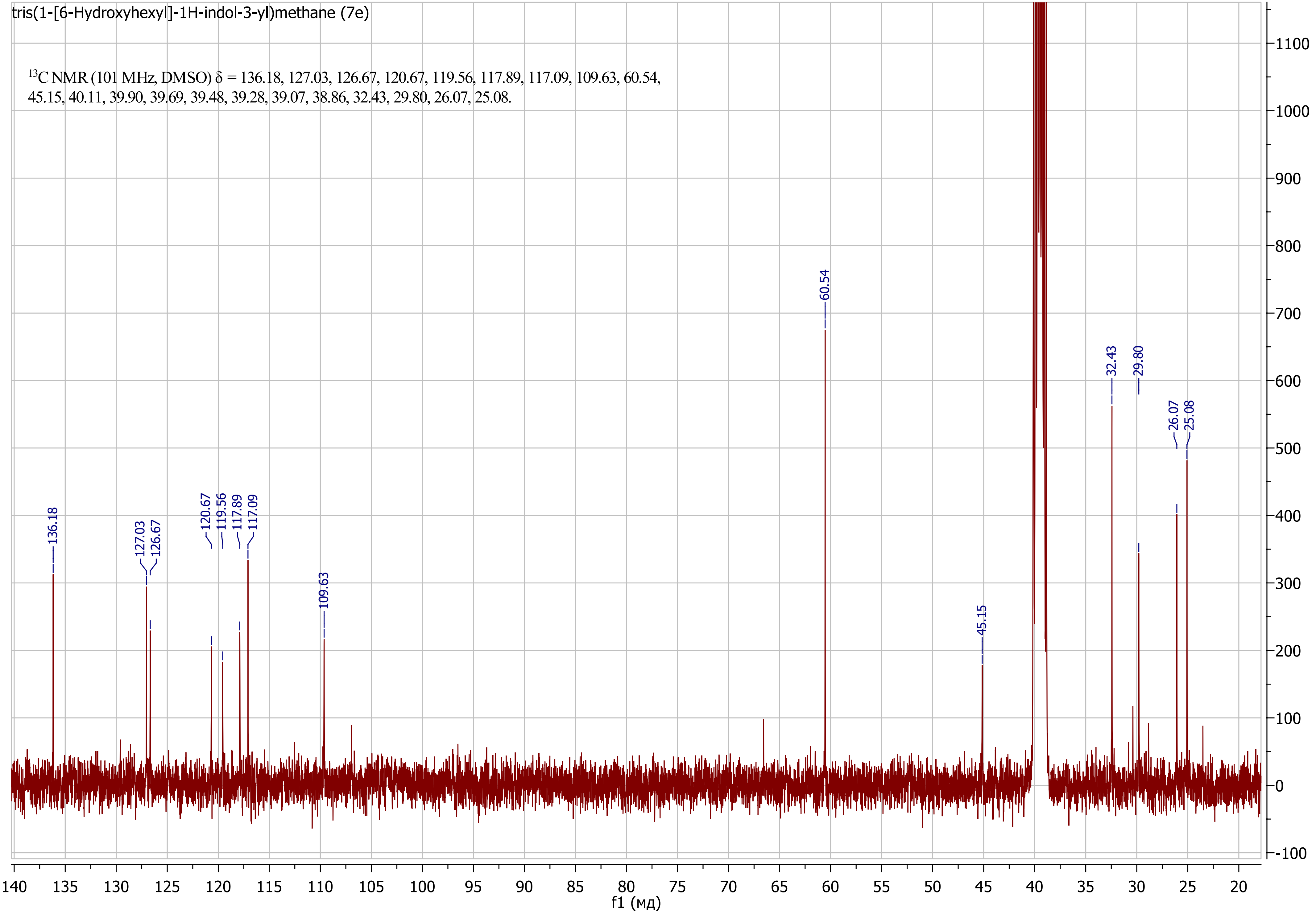

tris(1-[4-Hydroxybutyl]-1H-indol-3-yl)methane (7c)

^{1}H NMR (400 MHz, DMSO) δ 7.38 (d, *J* = 5.3 Hz, 6H), 7.05 (t, *J* = 7.3 Hz, 3H), 6.95 (s, 3H), 6.86 (t, *J* = 7.3 Hz, 3H), 6.02 (s, 1H), 4.33 (t, *J* = 4.5 Hz, 3H), 4.06 (s, 6H), 1.72 – 1.58 (m, 6H), 1.49 – 1.32 (m, 6H), 1.20 (d, *J* = 6.5 Hz, 6H).

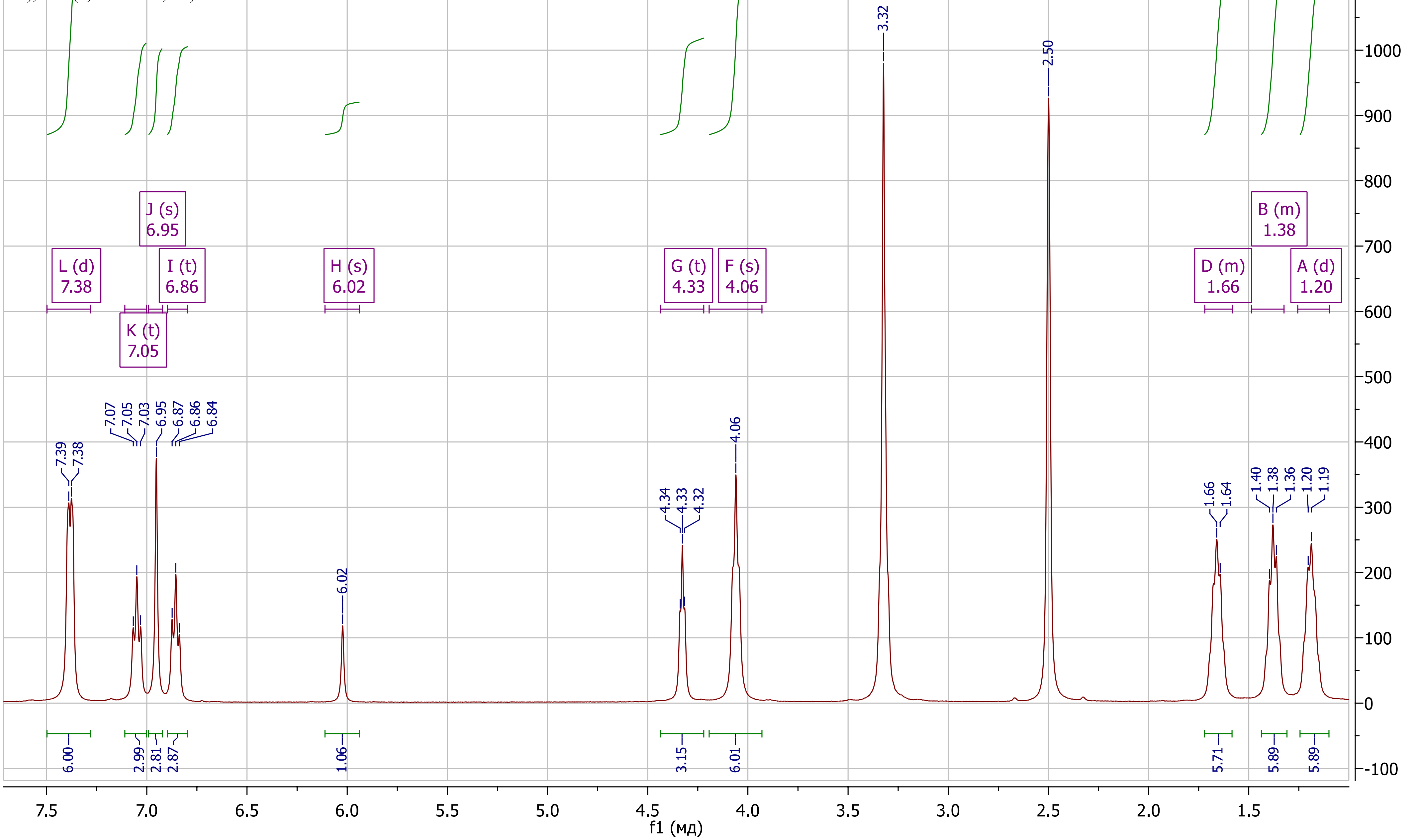

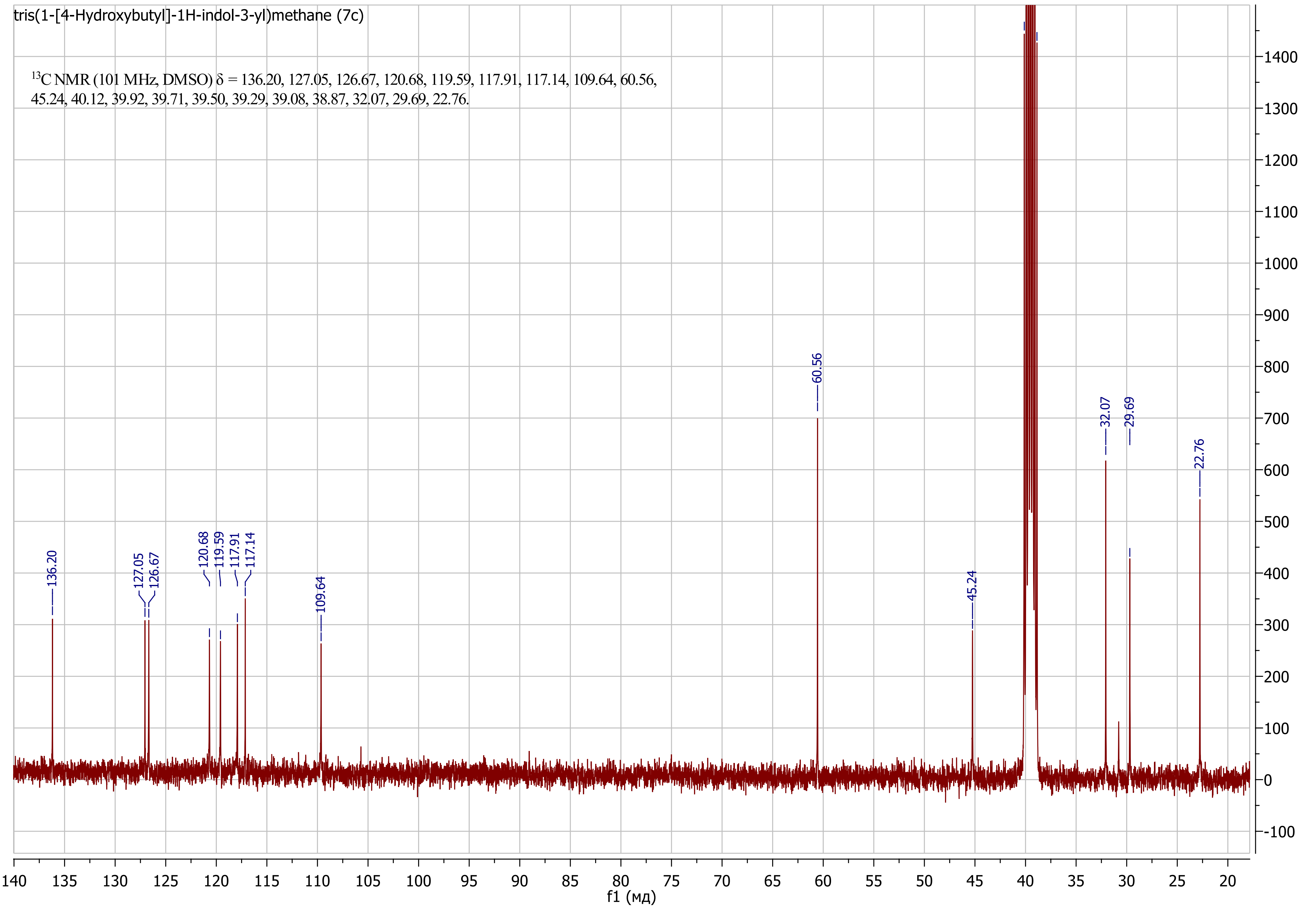
tris(1-[4-Hydroxybutyl]-1H-indol-3-yl)methane (7c)
13C NMR (101 MHz, DMSO) δ = 136.20, 127.05, 126.67, 120.68, 119.59, 117.91, 117.14, 109.64, 60.56, 45.24, 40.12, 39.92, 39.71, 39.50, 39.29, 39.08, 38.87, 32.07, 29.69, 22.76.
136.20
127.05
126.67
120.68
119.59
117.91
117.14
109.64
60.56
45.24
32.07
29.69
22.76
f1 (мд)

tris(1-(2-Hydroxyethyl)-1H-indol-3-yl)methylium chloride (8a)

^{1}H NMR (400 MHz, DMSO) δ 8.35 (s, 1H), 7.84 (d, J = 8.3 Hz, 1H), 7.40 (t, J = 7.9 Hz, 1H), 7.11 (d, J = 6.9 Hz, 2H), 4.92 (s, 1H), 4.51 (t, J = 5.2 Hz, 2H), 3.93 (s, 2H), 3.05 (s, 1H), 2.50 (s, 4H).

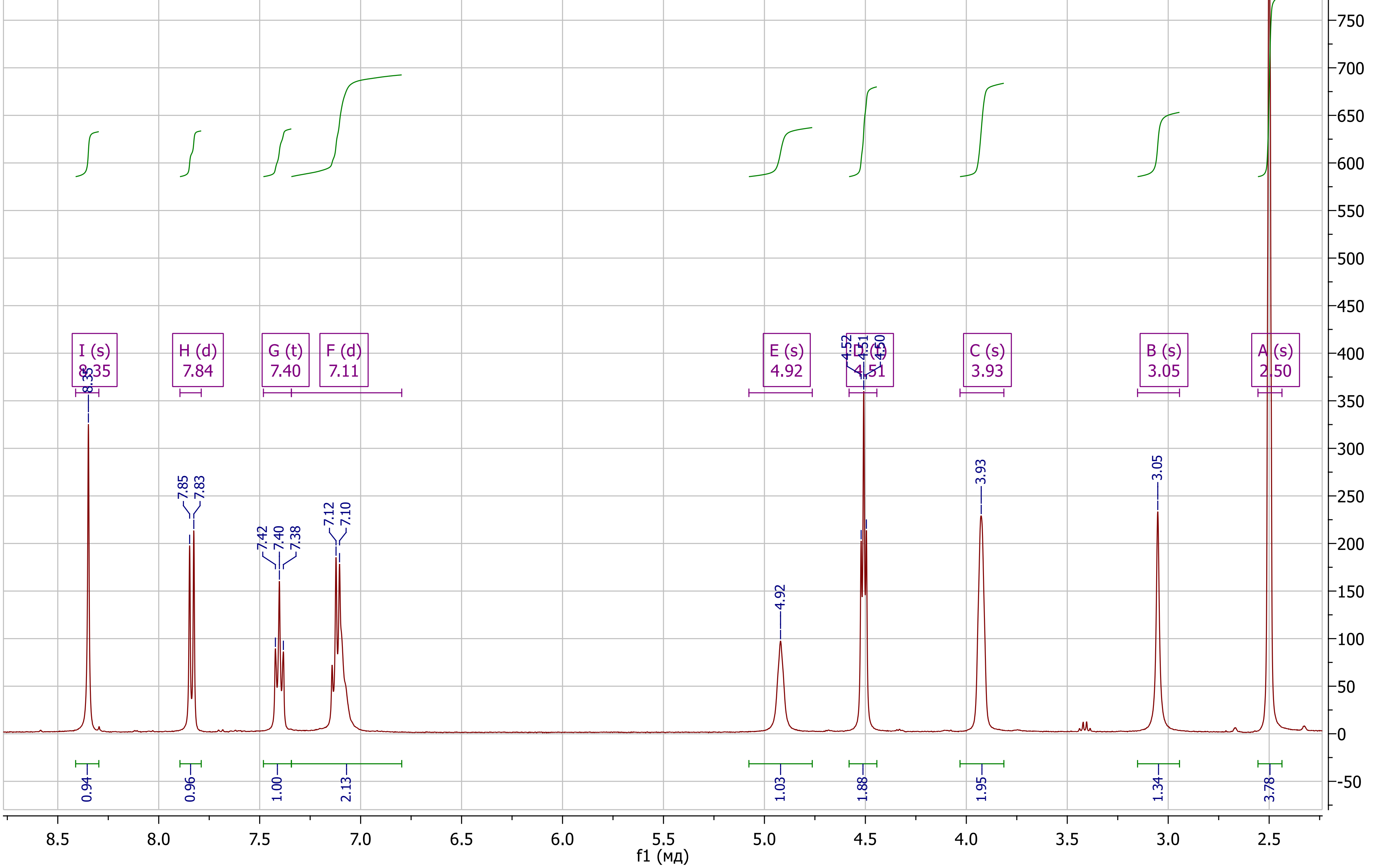

tris(1-(2-Hydroxyethyl)-1H-indol-3-yl)methylium chloride (8a)

^{13}C NMR (101 MHz, DMSO) δ = 138.69, 124.22, 122.98, 120.60, 112.00, 59.12, 49.57.

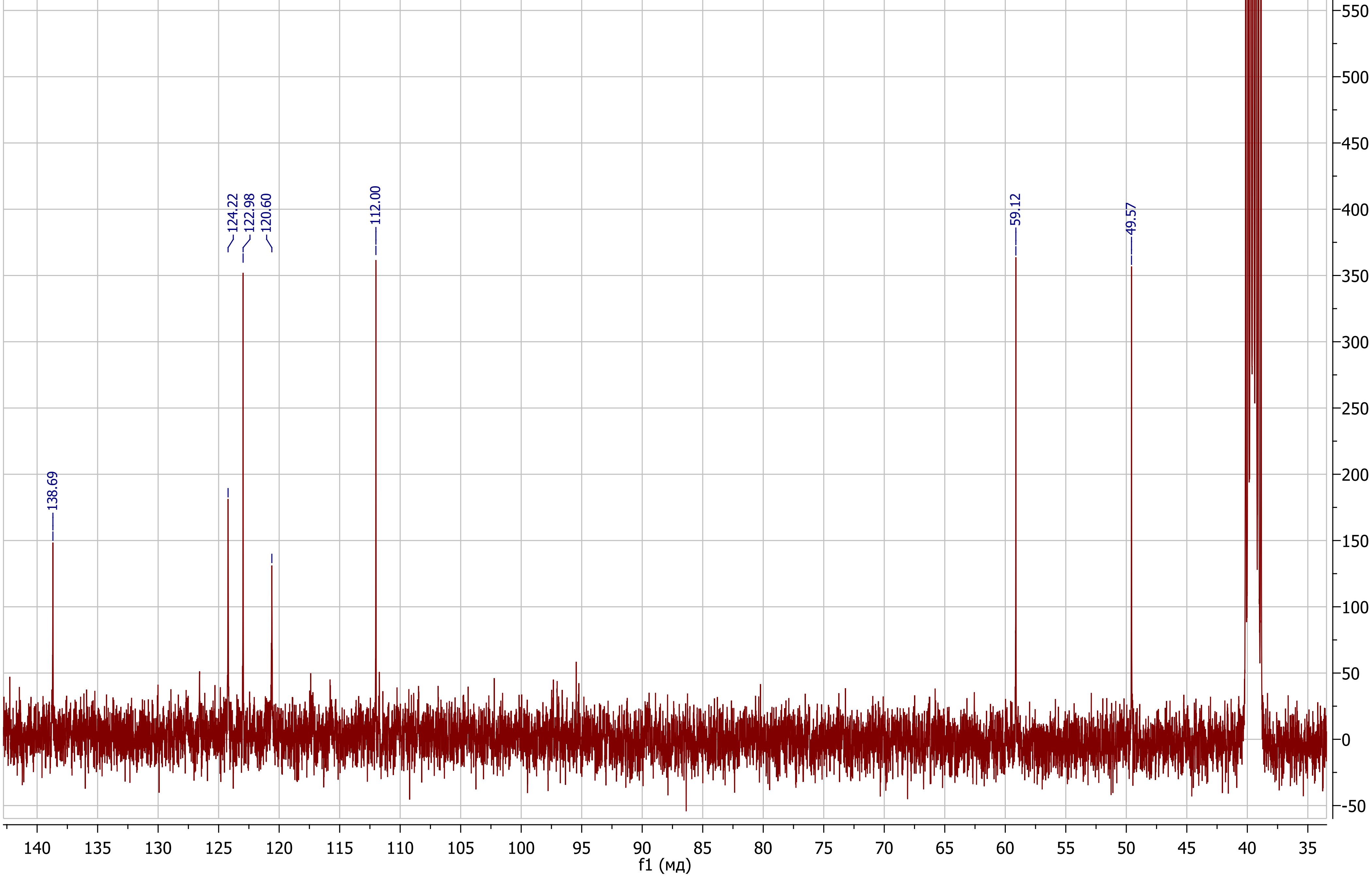

tris(1-(3-Hydroxypropyl)-1H-indol-3-yl)methylium chloride (8b)

^{1}H NMR (400 MHz, DMSO) δ 8.38 (s, 3H), 7.83 (d, *J* = 8.3 Hz, 3H), 7.41 (t, *J* = 7.6 Hz, 3H), 7.13 (t, *J* = 7.6 Hz, 3H), 7.02 (s, 3H), 4.52 (t, *J* = 6.9 Hz, 8H), 3.56 (t, *J* = 5.9 Hz, 6H), 3.04 (s, 16H), 2.32 – 1.92 (m, 7H).

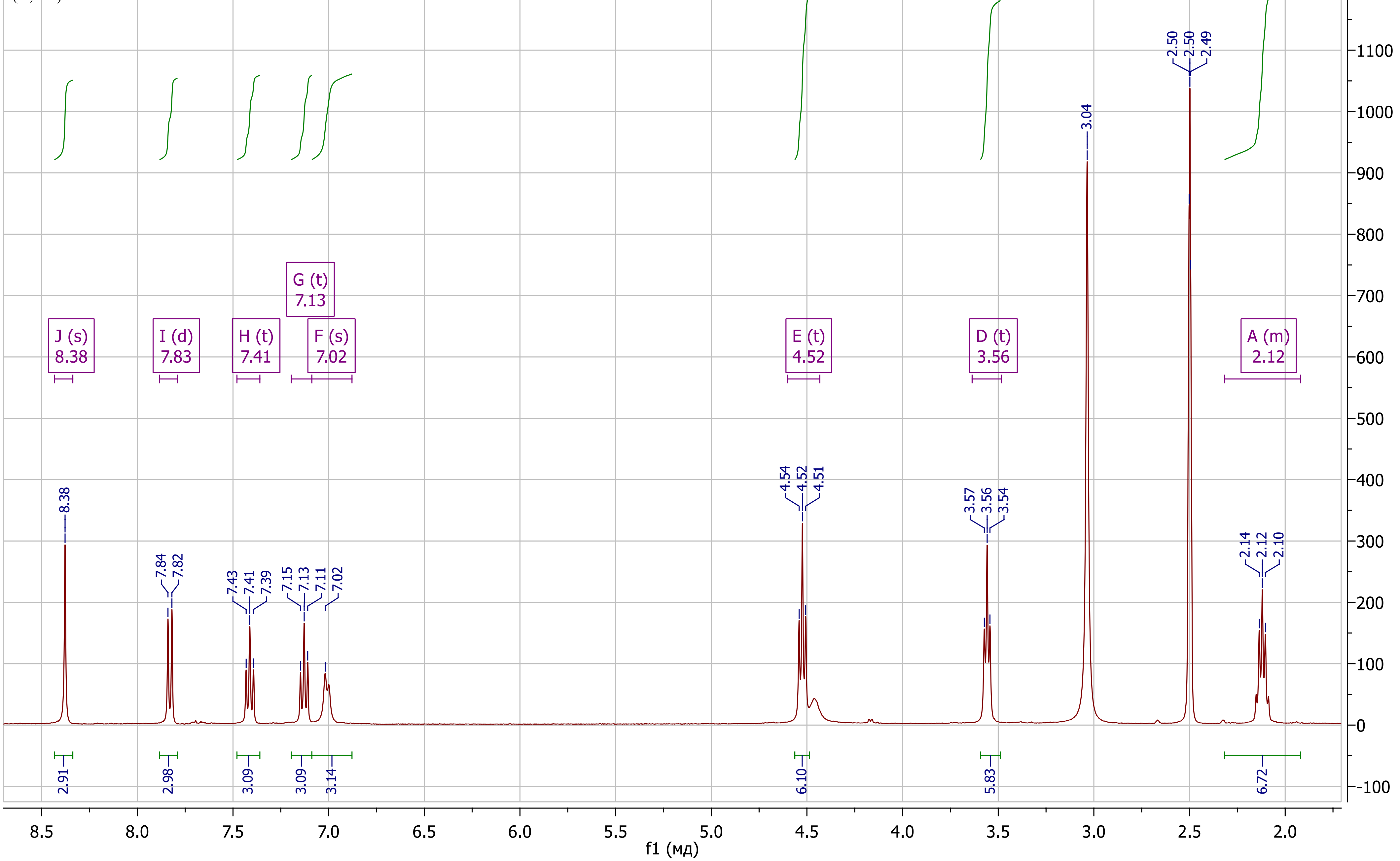

tris(1-(3-Hydroxypropyl)-1H-indol-3-yl)methylium chloride (8b)

^{13}C NMR (101 MHz, DMSO) δ = 157.77, 138.49, 124.35, 123.06, 120.49, 111.89, 57.47, 44.22, 40.13, 39.92, 39.71, 39.51, 39.30, 39.09, 38.88, 31.89.

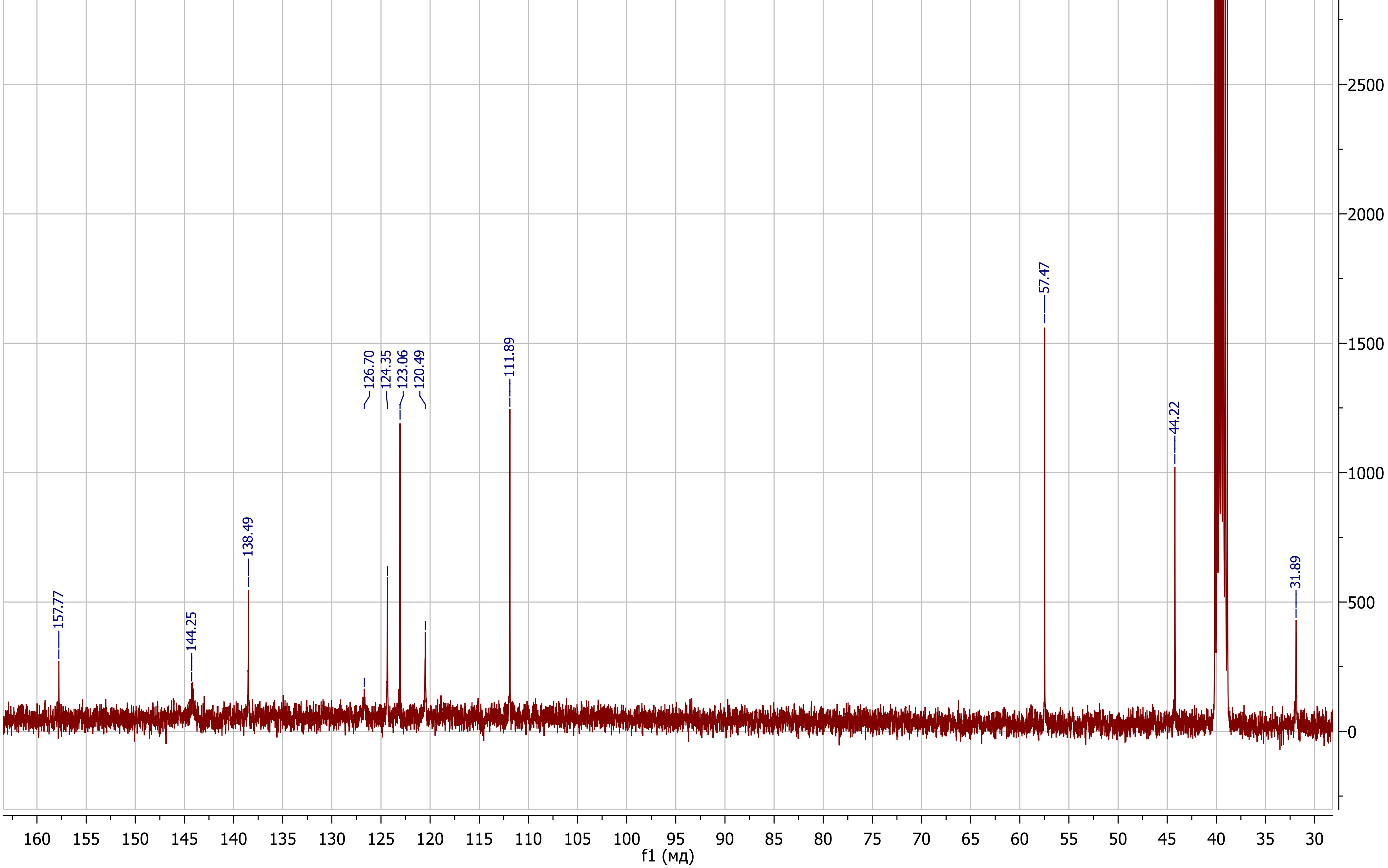

tris(1-(4-Hydroxybutyl)-1H-indol-3-yl)methylium chloride (8c)

^{1}H NMR (400 MHz, DMSO) δ 7.40 (d, J = 2.8 Hz, 3H), 7.38 (d, J = 3.3 Hz, 3H), 7.05 (t, J = 7.5 Hz, 3H), 6.96 (s, 3H), 6.86 (t, J = 7.4 Hz, 3H), 6.03 (s, 1H), 4.44 (t, J = 5.0 Hz, 3H), 4.07 (t, J = 6.8 Hz, 6H), 3.35 (dd, J = 11.5, 6.1 Hz, 7H), 1.90 – 1.48 (m, 6H), 1.48 – 1.11 (m, 6H).

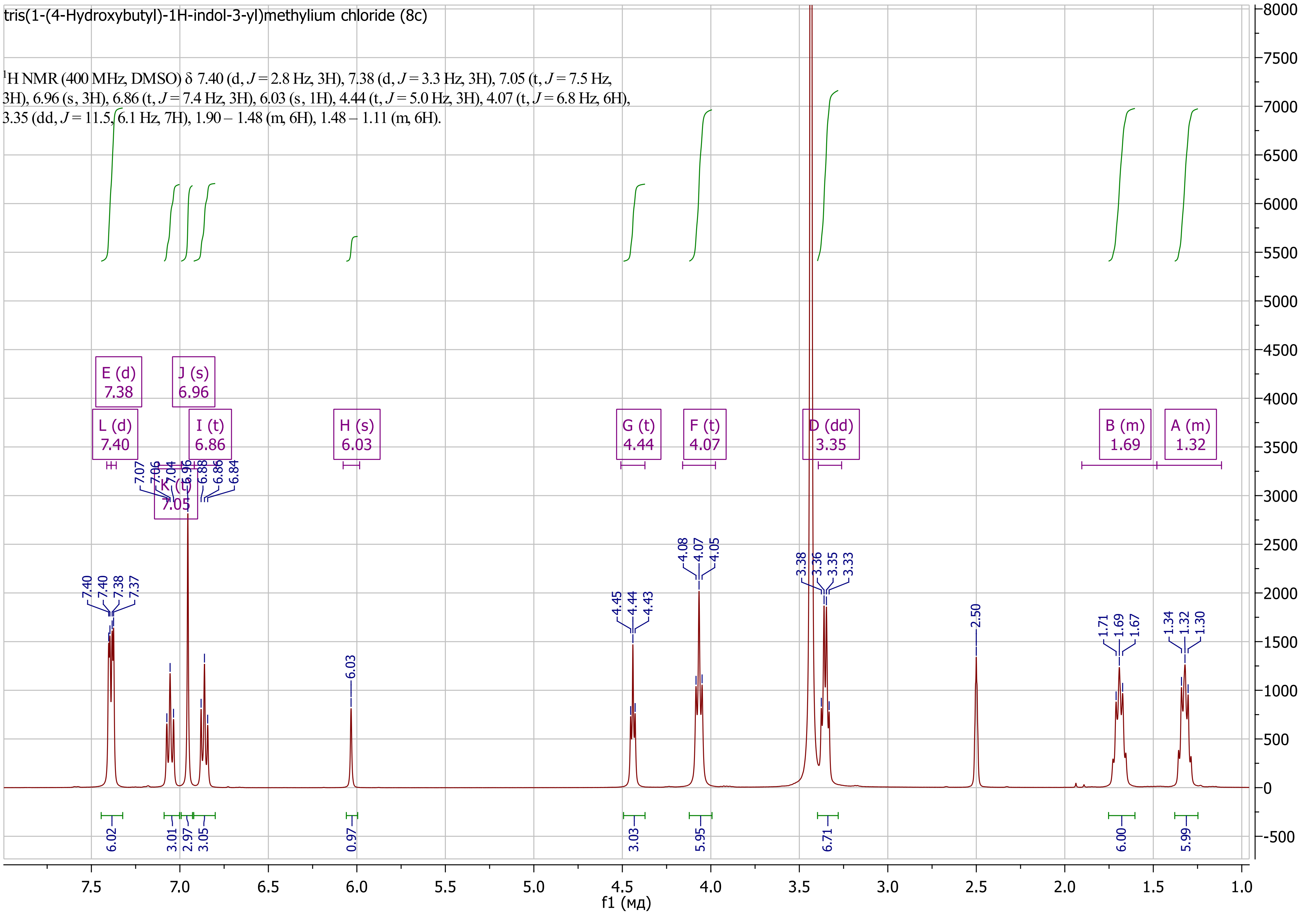

tris(1-(4-Hydroxybutyl)-1H-indol-3-yl)methylium chloride (8c)

^{13}C NMR (101 MHz, DMSO) δ = 136.31, 127.15, 126.74, 120.80, 119.68, 118.06, 117.30, 109.77, 60.35, 45.24, 30.87, 29.70, 26.65.

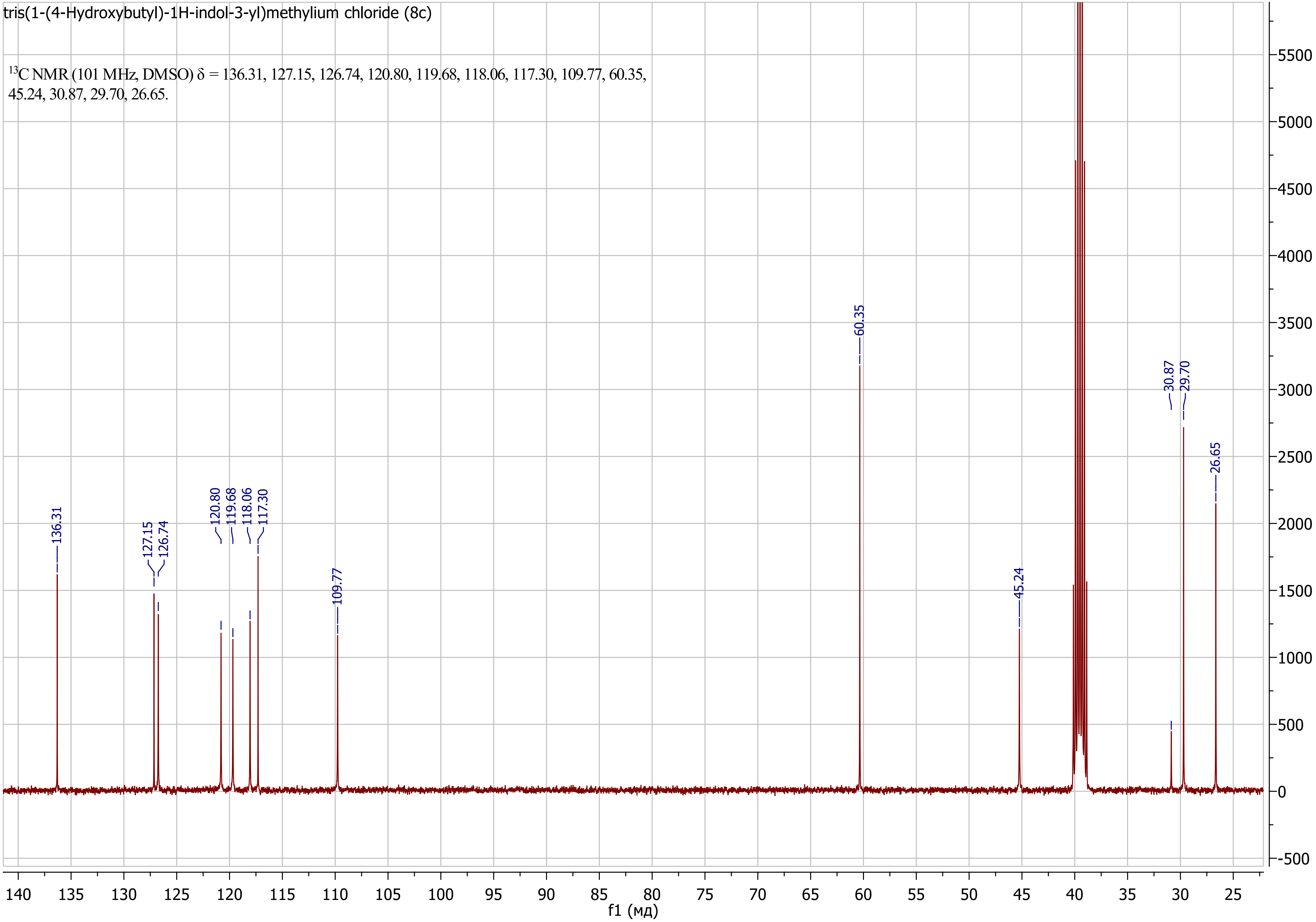

tris(1-(5-Hydroxypentyl)-1H-indol-3-yl)methylium chloride (8d)

^{1}H NMR (400 MHz, DMSO) δ 8.42 (s, 4H), 7.84 (d, *J* = 8.3 Hz, 4H), 7.41 (t, *J* = 7.6 Hz, 4H), 7.13 (t, *J* = 7.5 Hz, 4H), 7.01 (s, 4H), 4.46 (t, *J* = 7.0 Hz, 8H), 4.17 (s, 4H), 3.44 (s, 8H), 3.06 (s, 8H), 2.20 – 1.76 (m, 9H), 1.47 (ddd, *J* = 86.5, 46.3, 39.5 Hz, 19H), 1.23 – 1.08 (m, 1H).

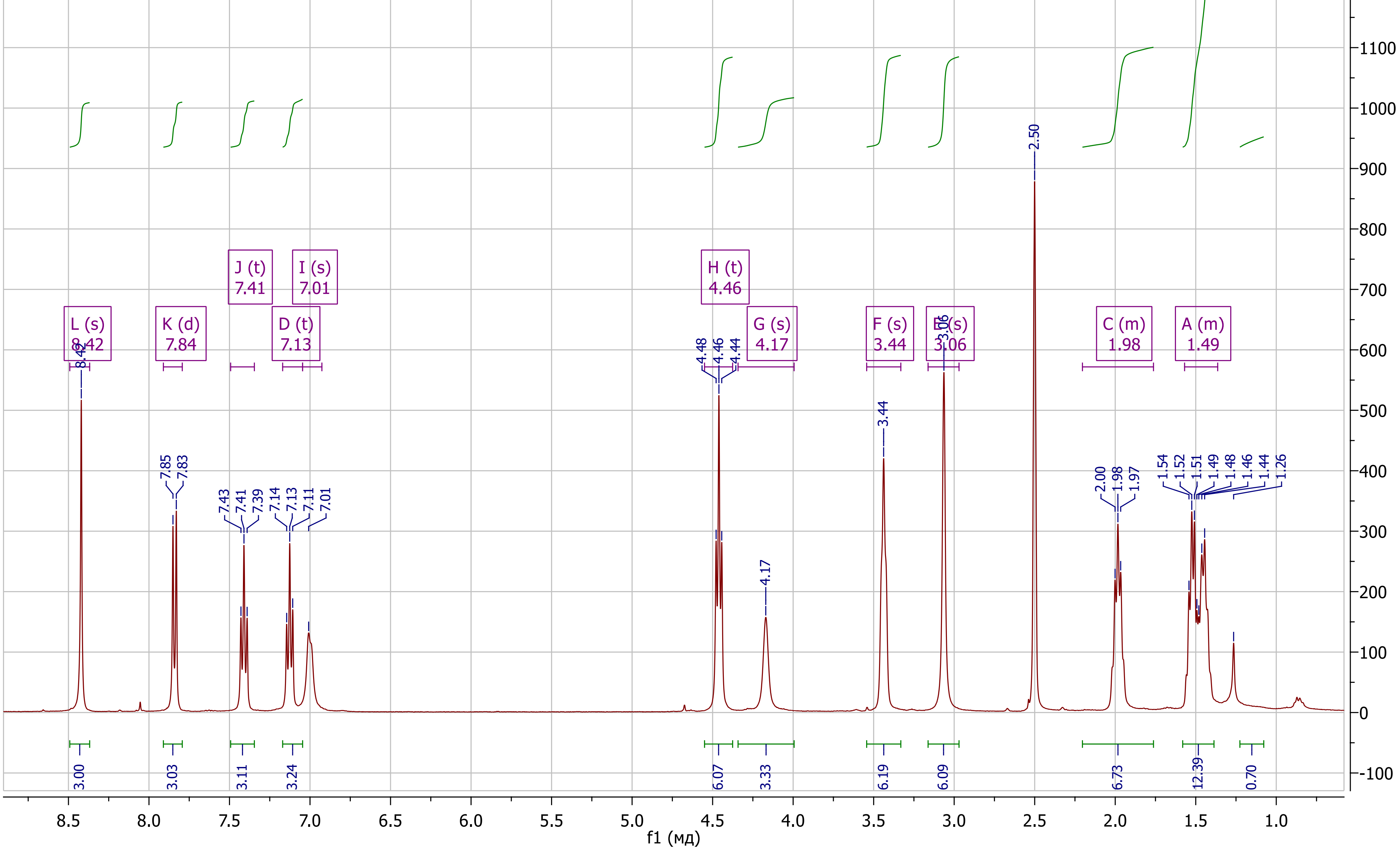

tris(1-(5-Hydroxypentyl)-1H-indol-3-yl)methylium chloride (8d)

^{13}C NMR (101 MHz, DMSO) δ = 138.47, 124.35, 123.06, 120.48, 111.94, 60.12, 46.93, 40.12, 39.91, 39.71, 39.50, 39.29, 39.08, 38.87, 31.52, 28.73, 22.38.

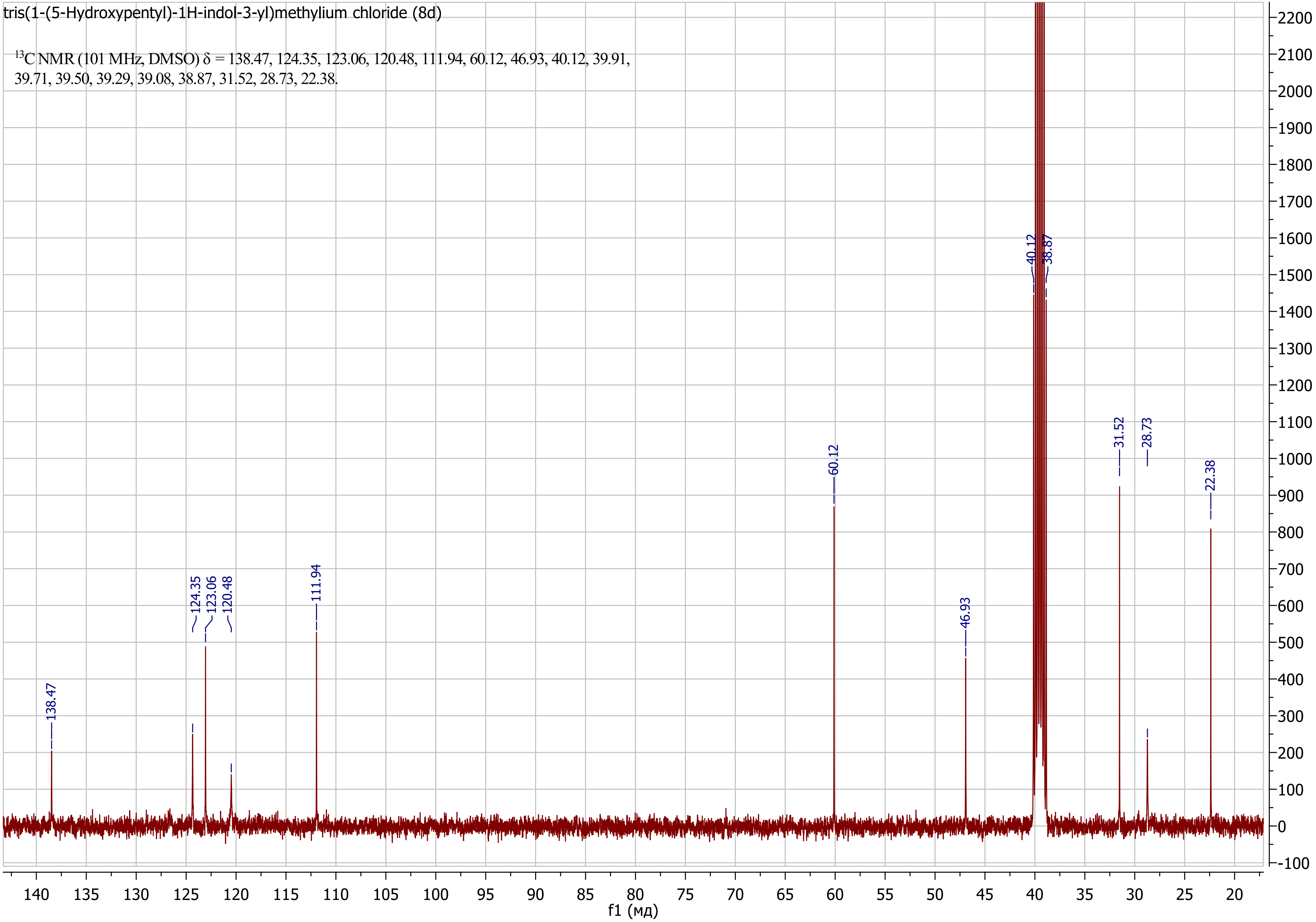

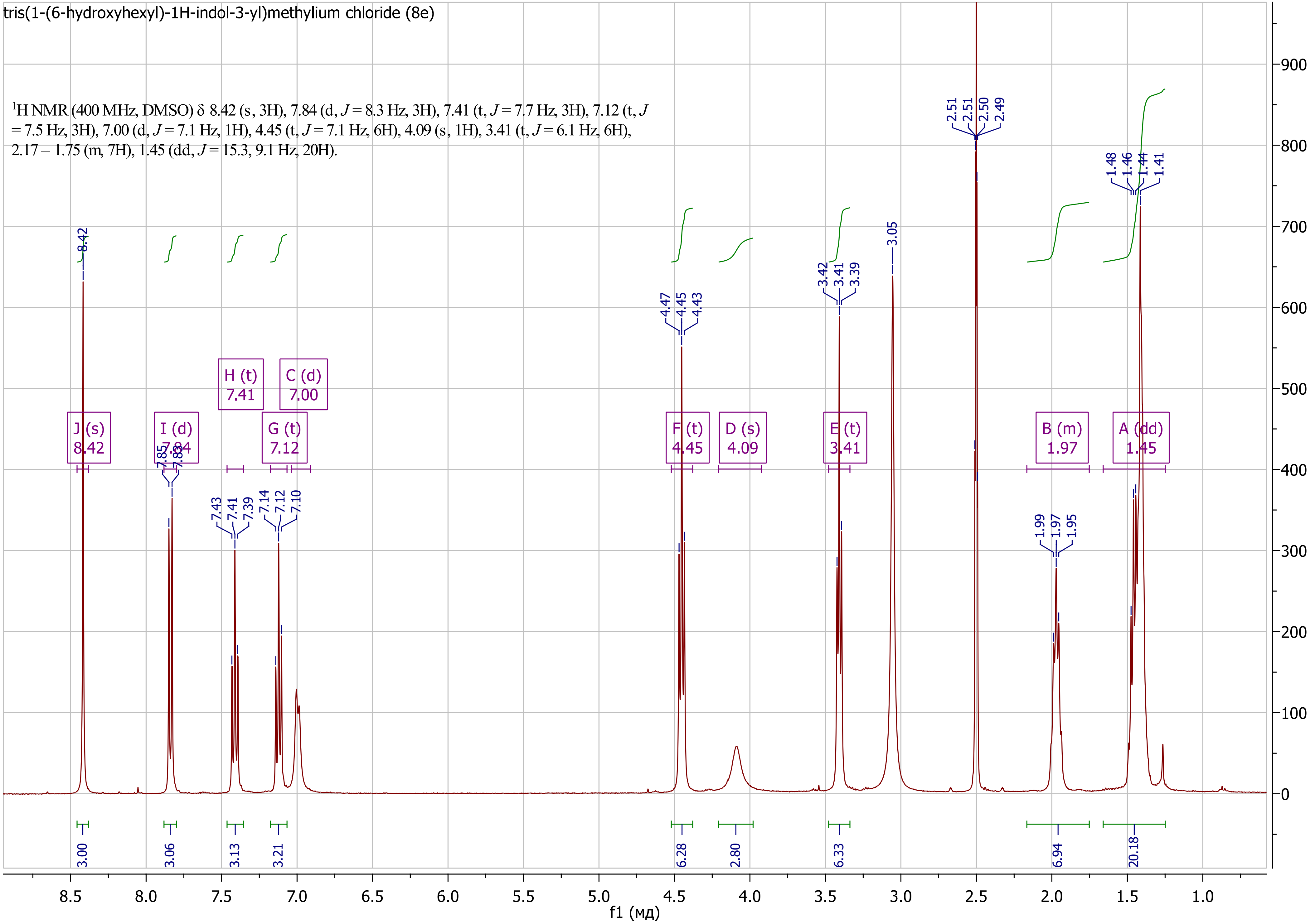
tris(1-(6-hydroxyhexyl)-1H-indol-3-yl)methylium chloride (8e)
1H NMR (400 MHz, DMSO) δ 8.42 (s, 3H), 7.84 (d, J = 8.3 Hz, 3H), 7.41 (t, J = 7.7 Hz, 3H), 7.12 (t, J = 7.5 Hz, 3H), 7.00 (d, J = 7.1 Hz, 1H), 4.45 (t, J = 7.1 Hz, 6H), 4.09 (s, 1H), 3.41 (t, J = 6.1 Hz, 6H), 2.17 – 1.75 (m, 7H), 1.45 (dd, J = 15.3, 9.1 Hz, 20H).
J (s) 8.42
I (d) 7.84
H (t) 7.41
G (t) 7.12
C (d) 7.00
F (t) 4.45
D (s) 4.09
E (t) 3.41
B (m) 1.97
A (dd) 1.45
3.00
3.06
3.13
3.21
6.28
2.80
6.33
6.94
20.18
f1 (мд)

tris(1-(6-hydroxyhexyl)-1H-indol-3-yl)methylium chloride (8e)

^{13}C NMR (101 MHz, DMSO) δ = 157.82, 138.49, 124.36, 123.03, 120.44, 111.93, 60.24, 46.89, 40.14, 39.93, 39.72, 39.51, 39.30, 39.09, 38.88, 31.87, 28.87, 25.61, 24.68.

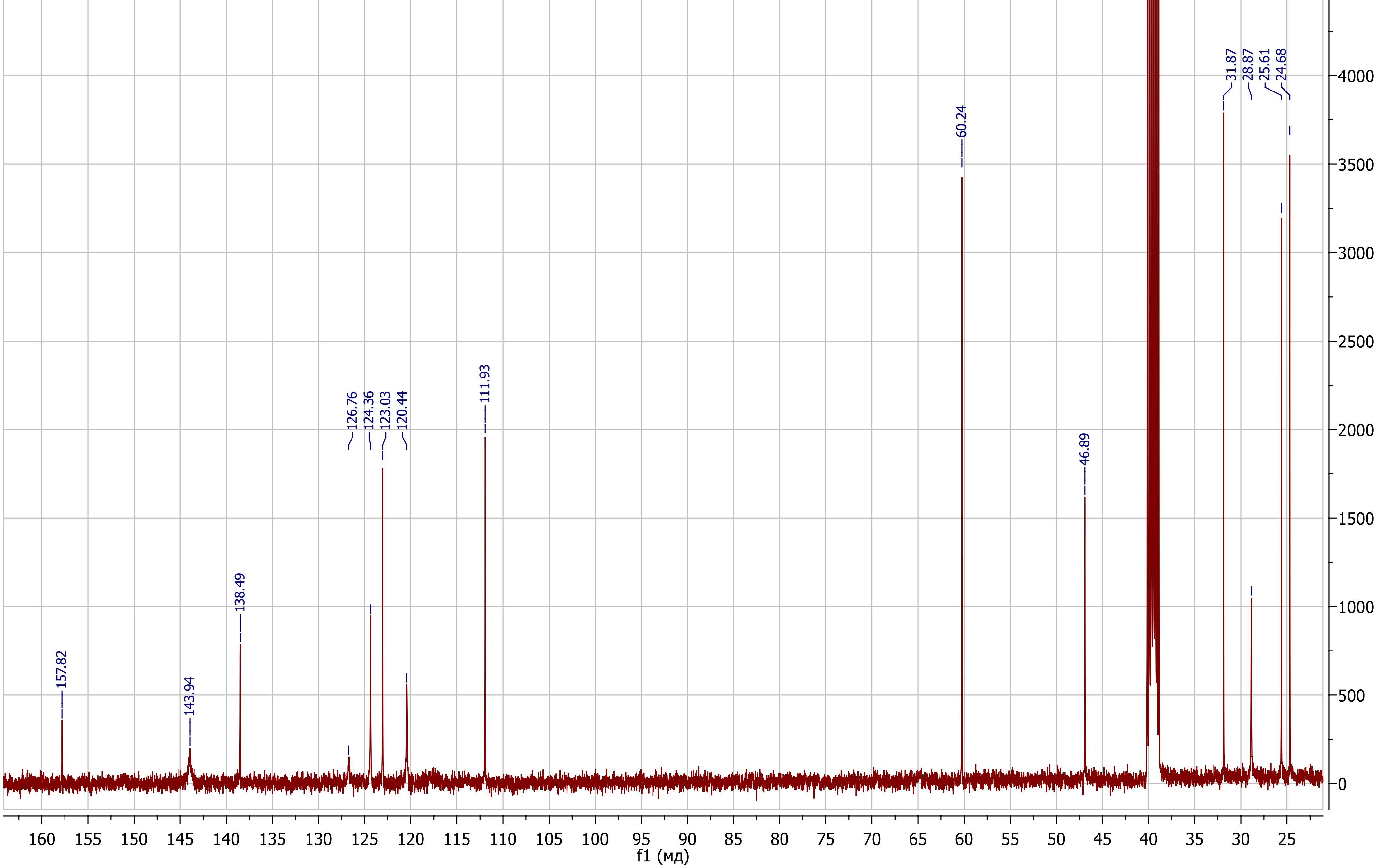

Supplement: Supplementary file 1 [file pharmaceuticals-13-00469-s001.pdf]
